# Supplementary material for: Mechanistic exploration of polytetrafluoroethylene thermal plasma gasification through multiscale simulation coupled with experimental validation
Source: Nat Commun. 2024 Feb 23;15:1654. doi: 10.1038/s41467-024-45077-6 (PMC10891128; doi:10.1038/s41467-024-45077-6)
Supplement: Supplementary file 1 — Supplementary Information [file 41467_2024_45077_MOESM1_ESM.pdf]

SUPPLEMENTARY INFORMATION

Mechanistic Investigation of Thermal Plasma Gasification of Polytetrafluoroethylene via Multiscale Simulation Coupled with Experimental Confirmation

Chu Chu[1], Long Long Ma[2], Hyder Alawi [1], Wenchao Ma[1*], YiFei Zhu[3], Junhao Sun[4], Yao Lu[5], Yixian Xue[1], Guanyi Chen[1,6,7]

[1] School of Environmental Science and Engineering, Tianjin University/Tianjin Key Lab of Biomass/Wastes Utilization, Tianjin 300072, China

[2]School of Energy &Environment, Key Lab Energy Thermal Conversion & Control, Southeast University, Nanjing 210096, China

[3] School of Electrical Engineering, Xi'an Jiaotong University, Xi'an 710049 , China

[4] Postdoctoral Programme, Guosen Securities, Shenzhen 518001, China

[5] School of Chemical Engineering and Technology, Hebei University of Technology, Tianjin 300401, China

[6] School of Ecology and Environment, Tibet University, Lhasa 850012, Tibet, China

[7] School of Mechanical Engineering, Tianjin University of Commerce, Tianjin 300314, China

Supplementary Table 1. The experimental fluorine distribution in the product streams identified by IC

Chemical state of fluorine	Mass percentage (wt.%)				
Mixed atmosphere (O_2/C=2.7, H_2O/C=2.7) under different temperature					
	3300K	3400K	3600K	3800K	4000K
Inorganic F in gas	74.04	77.35	73.72	76.69	75.38
Organic F in gas	0.98361	0.4918	1.72131	0.98361	1.22951
Total F in solid	1.235	0.68241	0.52353	0.38464	0.19962
Oxygen atmosphere (3300K) under different O_2/C ratio					
	0.93	1.86	2.79	3.72	4.65
Inorganic F in gas	56.89	52.98	51.26	58.44	59.84
Organic F in gas	19.61	23.4	25.24	18.06	16.65
Total F in solid	1.01	1.16	0.44	0.31	0.68
Steam atmosphere (3300K) under different H_2O/C ratio					
	0.93	1.86	2.79	3.72	4.65
Inorganic F in gas	66.87	70.43	72.07	74.96	75.09
Organic F in gas	8.64	8.01	1.27	3.57	1.19
Total F in solid	2.01	0.96	0.84	1.01	0.68

Supplementary Table 2. Main compounds in the gaseous products from PTFE thermal plasma degradation identified by GC-MS

Rt (min)	Compounds	Molecular formular	Characteristic peak (m/z)	Peak area (%)				
Mixed atmosphere (O_2/C=2.7, H_2O/C=2.7) under different temperature								
				3300K	3400K	3600K	3800K	4000K
3.888	Carbon dioxide	CO_2	44	80.918	78.204	78.616	74.256	74.451
14.580	Carbon monoxide	CO	28	9.283	11.090	8.368	14.883	14.323
3.577	Carbonic difluoride	COF_2	28,47,66	1.936	1.939	4.215	4.013	3.931
6.317	Formic acid	HCOOH	17,18,28,29,44,45,46	0.000	1.351	0.000	1.399	0.000
4.254	Methane, trifluoro-	CHF_3	31,50,51,69	2.053	0.000	2.096	0.000	0.000
3.037	Ethane, hexafluoro-	C_2F_6	31,50,69,119	1.228	1.005	1.557	0.461	1.463
11.031	Acetamide, 2-fluoro-	C_2H_4FNO	33,44,77	0.202	0.409	0.382	0.549	0.259
3.782	Propane, octafluoro-	C_3F_8	31,69,169	1.746	1.119	1.497	0.663	2.145
5.014	Perfluorocyclobutane	C_4F_8	31,50,69,100,131	0.468	0.855	1.313	1.472	0.226
6.017	Cyclopentanone	C_5H_8O	26,27,28,29,39,41,42,55,56,84	1.463	2.025	0.754	0.851	1.876
8.336	Benzene, 1,2,3,4-tetrafluoro-	$C_6H_2F_4$	31,75,81,99,119,150	0.505	0.369	0.787	0.904	0.934
Oxygen atmosphere (3300K) under different O_2/C ratio								
				0.93	1.86	2.79	3.72	4.65
3.888	Carbon dioxide	CO_2	44	21.000	21.860	24.260	29.340	31.660
14.580	Carbon monoxide	CO	28	5.790	4.910	4.470	4.790	4.480
3.577	Carbonic difluoride	COF_2	28,47,66	51.830	54.980	46.090	44.580	49.400
2.782	Methane, tetrafluoro-	CF_4	50,69	15.120	16.760	13.170	20.580	13.170
3.155	Ethene, tetrafluoro-	C_2F_4	31,50,81,100	2.450	0.000	4.990	0.000	0.000
3.037	Ethane, hexafluoro-	C_2F_6	31,50,69,119	3.390	0.000	6.880	0.000	0.000
3.782	Propane, octafluoro-	C_3F_8	31,69,169	0.430	1.490	0.150	0.710	1.280
Steam atmosphere (3300K) under different H_2O/C ratio								
				0.93	1.86	2.79	3.72	4.65
3.888	Carbon dioxide	CO_2	44	27.845	46.961	36.227	46.650	56.092
6.317	Formic acid	HCOOH	17,18,28,29,44,45,46	2.026	0.000	8.928	15.603	6.594
12.497	Methane	CH_4	14,15,16	0.000	0.000	0.000	0.135	0.000
14.580	Carbon monoxide	CO	28	16.662	13.661	15.200	16.310	18.285
3.577	Carbonic difluoride	COF_2	28,47,66	9.379	2.718	13.436	0.000	0.000
2.9375	Methane, fluoro-	CFH_3	15,33,34	0.000	5.755	3.461	1.145	7.638
3.282	Ethene, 1,1-difluoro-	$C_2F_2H_2$	14,31,33,44,45,63,64	9.407	0.000	0.000	0.000	0.000
3.155	Ethene, tetrafluoro-	C_2F_4	31,50,81,100	7.105	0.000	13.273	16.089	0.000
5.244	Ethene	C_2H_4	26,27,28	1.902	8.073	4.010	4.069	6.709
4.264	1-Propene, 1,1,2,3,3,3-hexafluoro-	C_3F_6	28,31,69,81,100,131,150	0.000	6.997	0.000	0.000	0.000
8.493	Propan-1-ol	C_3H_8O	27,29,31	0.000	0.000	7.466	0.000	3.182
4.367	Perfluorobuta-1,3-diene	C_4F_6	31,93,112,162	16.629	0.000	0.000	0.000	0.000
10.191	4-methylpent-3-enoic acid	$C_6H_{10}O_2$	43,58	10.044	12.835	0.000	0.000	0.000

Supplementary Table 3. Quantitative Analysis of PFAS in methanol absorption solution using LC/MS

Rt (min)	Compounds	Molecular formular	Concentration (ppb)				
Mixed atmosphere (O_2/C=2.7, H_2O/C=2.7) under different temperature							
			3300K	3400K	3600K	3800K	4000K
6.72	PFUNA	$C_{11}HF_{21}O_2$	0.6	0.6	0.0	0.6	0.6
6.3	PFNA	$C_9HF_{17}O_2$	0.9	0.0	0.0	0.0	0.9
5.98	PFOA	$C_8HF_{15}O_2$	0.7	0.7	0.7	0.7	0.7
5.67	PFHPA	$C_7HF_{13}O_2$	0.7	0.7	0.7	0.7	0.8
5.36	PFHxA	$C_6HF_{11}O_2$	0.7	0.7	0.7	0.7	0.7
5.01	PFPA	$C_6F_{10}O_3$	0.7	0.7	0.5	0.5	0.6
6.55	PFDA	$C_{10}HF_{19}O_2$	0.9	0.0	0.0	0.0	0.9
6.35	PFHpS	$C_7F_{15}SO_3H$	0.0	1.0	0.0	0.0	0.9
5.32	HPFHpA	$C_7H_2F_{12}O_2$	0.0	0.7	0.6	0.7	0.7
Oxygen atmosphere (3300K) under different O_2/C ratio							
			0.93	1.86	2.79	3.72	4.65
6.83	PFUNA	$C_{11}HF_{21}O_2$	0.6	0.0	0.9	0.0	0.1
7.32	PFOS	$C_8HF_{17}O_3S$	0.0	0.0	0.2	1.8	0.0
5.98	PFOA	$C_8HF_{15}O_2$	0.7	0.7	0.0	0.0	0.0
5.69	PFHPA	$C_7HF_{13}O_2$	0.7	0.7	0.0	0.1	0.0
5.36	PFHxA	$C_6HF_{11}O_2$	0.7	0.7	0.0	0.0	0.1
5.02	PFPA	$C_6F_{10}O_3$	0.6	0.7	0.3	0.0	0.0
5.32	HPFHpA	$C_7H_2F_{12}O_2$	0.7	0.7	0.0	0.0	0.0
5.46	PFBS	$C_4HF_9O_3S$	0.9	0.0	0.0	0.1	0.0
Steam atmosphere (3300K) under different H_2O/C ratio							
			0.93	1.86	0.0	3.72	4.65
5.36	PFBA	$C_4HF_7O_2$	0.1	0.0	0.1	0.0	0.0
7.10	PFDoDA	$C_{11}F_{23}COOH$	0.5	0.0	0.0	0.0	0.0
7.53	PFUNA	$C_{11}HF_{21}O_2$	0.1	0.4	0.1	0.0	0.1
7.32	PFOS	$C_8HF_{17}O_3S$	0.0	0.5	0.4	0.5	0.8
6.41	PFHPA	$C_7HF_{13}O_2$	0.0	0.1	0.0	0.0	0.0
6.15	PFHxA	$C_6HF_{11}O_2$	0.1	0.0	0.0	0.0	0.0
5.75	PFPA	$C_6F_{10}O_3$	0.6	0.3	0.4	0.6	0.4

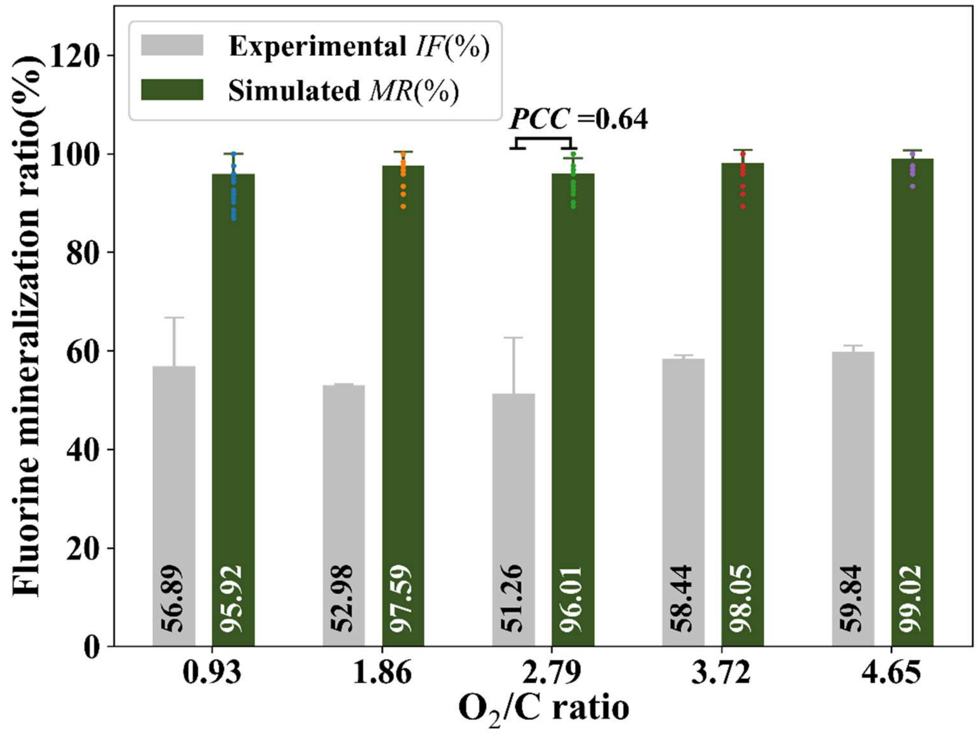

Supplementary Figure 1. The comparison of experimental inorganic fluorine ratio and ReaxFF MD simulated mineralized fluorine ratio. The *PCC* represents the Pearson correlation coefficients. The mineralized fluorine includes inorganic fluorides (HF, HOF, F-, F2) and fluorinated C1 compounds (such as COF_2, CF_3OH, FCOOH). Error bars represent standard deviations (SD) calculated from three measurements.

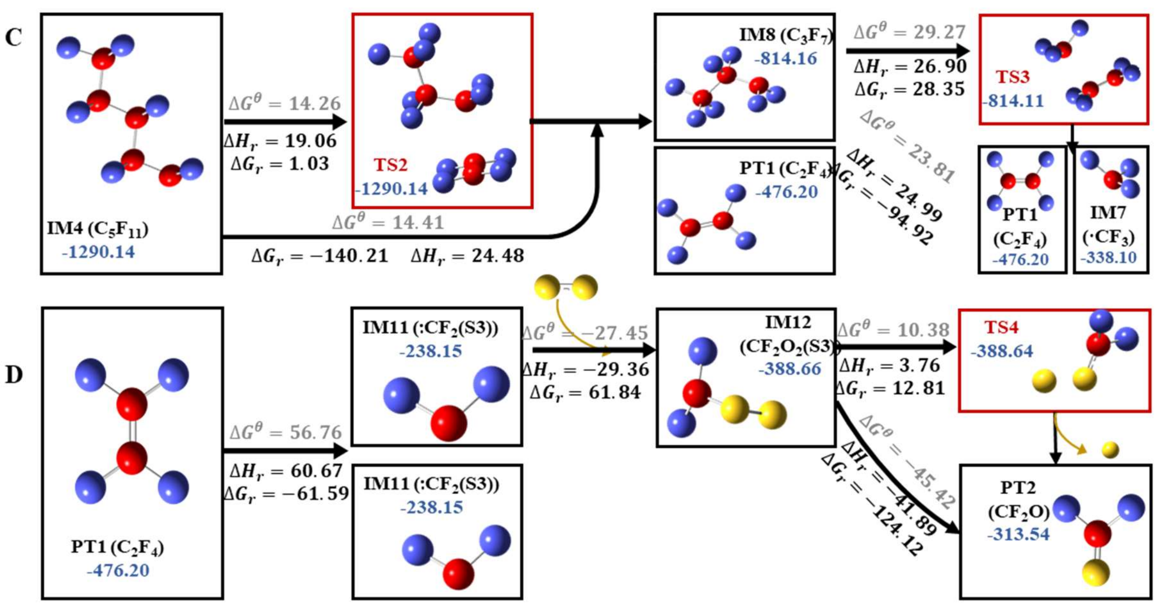

Supplementary Figure 2. DFT calculations on relative energy and structure changes, and energy barriers for the pyrolysis of $\cdot C_5F_{11}$ and $\cdot C_3F_7$, and the main reactions in chain termination stage under oxygen atmosphere. The color codes for the atoms are red: C, violet: F, yellow: O. θ represents the energy barrier in standard state (298.15K, 1atm). * indicates the transition state. r represents the energy and enthalpic barrier in reaction state (3300K, 1 atm). The energies are expressed in units of kilocalories per mole.

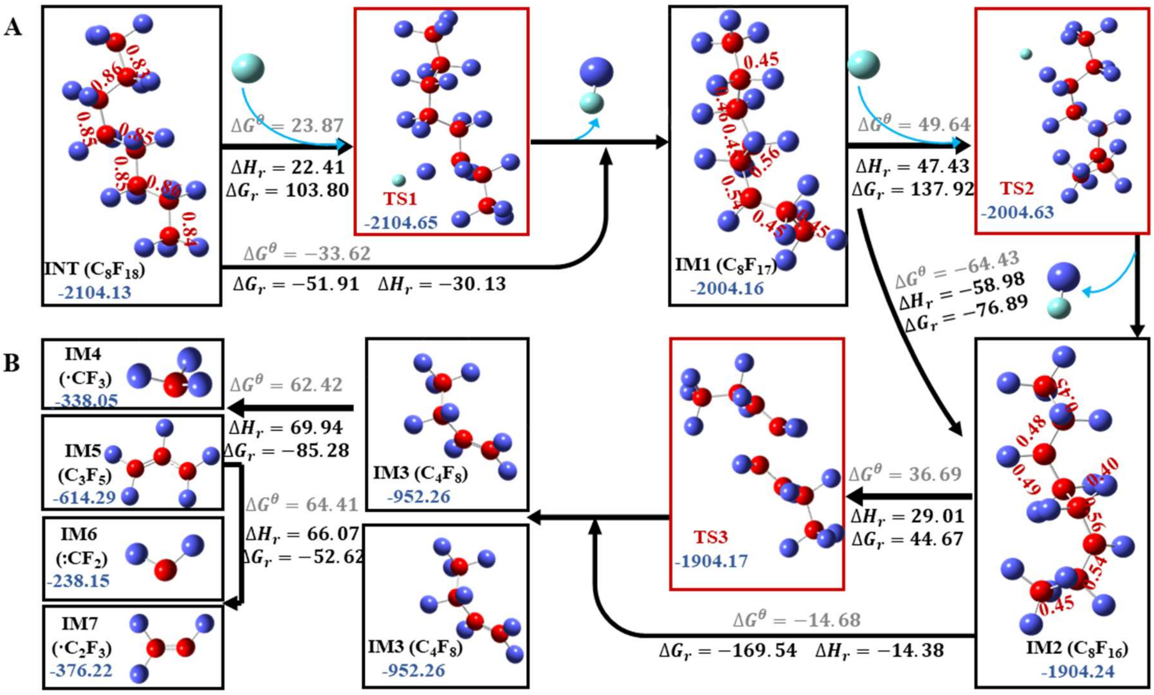

Supplementary Figure 3. DFT calculations on relative energy and structure changes, and energy barriers for the main reactions in chain initiation and transfer stage under steam atmosphere. The color codes for the atoms are red: C, violet: F, yellow: O, cyan: H. θ represents the energy barrier in standard state (298.15K, 1atm). * indicates the transition state. r represents the energy and enthalpic barrier in reaction state (3300K, 1 atm). The energies are expressed in units of kilocalories per mole.

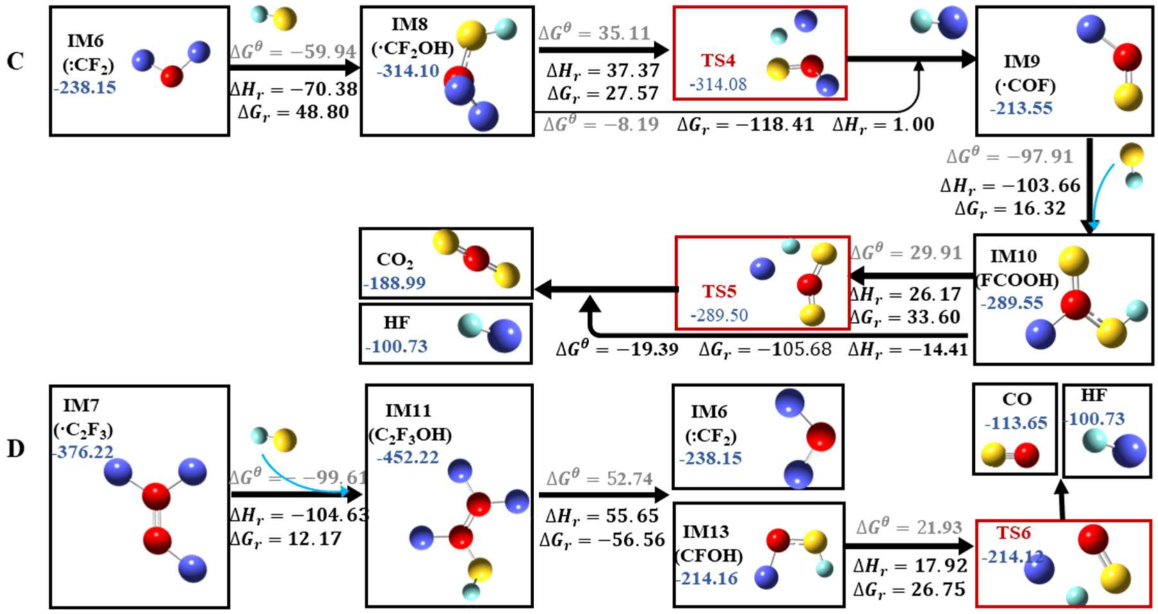

Supplementary Figure 4. DFT calculations on relative energy and structure changes, and energy barriers for the main reactions in chain termination stage under steam atmosphere. The color codes for the atoms are red: C, violet: F, yellow: O, cyan: H. θ represents the energy barrier in standard state (298.15K, 1atm). * indicates the transition state. r represents the energy and enthalpic barrier in reaction state (3300K, 1 atm). The energies are expressed in units of kilocalories per mole.

Supplementary Discussion 1. XYZ Coordinates of Optimized DFT Structures and Corresponding Energies (θ represents the energy barrier in standard state (298.15K, 1atm). * indicates the transition state. r represents the energy and enthalpic barrier in reaction state (3300K, 1 atm)

The oxygen atmosphere (Fig. 4 and Supplementary Fig.1)

1. INT (C_8F_{18})

EE[B3LYP/6−311G**] = -2102.55315 Ha

$Hcorr^{\theta}$ [B3LYP/6−31+G(d,p)]= 0.12786 Ha

$Gcorr^{\theta}$[B3LYP/6−31+G(d,p)]= 0.04819 Ha

$H^{\theta}= EE+ Hcorr^{\theta}$= -2102.4252 Ha

$G^{\theta}= EE+$ Gcorr^{θ}= -2102.50495 Ha

$Hcorr^{r}$[B3LYP/6−31+G(d,p)]= 0.79927 Ha

$Gcorr^{r}$[B3LYP/6−31+G(d,p)]= -1.58057 Ha

$H^{r}= EE+ Hcorr^{r}$= -2101.75387 Ha

$G^{r}= EE+$ Gcorr^{r}= -2104.13372 Ha

C	-2.93993000	-0.68497300	-0.10424800
C	-4.41141100	-0.23476400	0.10573100
F	-2.85949400	-1.28229000	-1.31298900
F	-2.64414800	-1.58652700	0.86198900
F	-5.22309800	-1.25839400	-0.18341800
F	-4.70351200	0.79324400	-0.70282700
F	-4.61492900	0.13234800	1.37486100
C	-0.43258200	-0.01104500	0.11845200
C	-1.90479300	0.47624000	-0.05588500
F	-0.23097400	-1.08780500	-0.68009400
F	-0.26118900	-0.37360900	1.40944800
F	-2.20787400	1.28530100	0.98584400
F	-2.00802200	1.17802000	-1.20800800
C	2.08823200	0.81116400	0.24661700
C	0.62691800	1.06601200	-0.25212000
F	2.10357000	0.90523600	1.59359200

F	2.83407400	1.81927100	-0.27127500
F	0.24781400	2.24395700	0.30265100
F	0.65730100	1.20062200	-1.59478100
F	4.68916500	-1.85717600	-0.12070300
C	4.27876600	-0.59588400	0.05636000
C	2.74243200	-0.53450200	-0.16324400
F	4.59006700	-0.20841500	1.30022300
F	4.91052900	0.19096200	-0.82018400
F	2.50675700	-0.76704800	-1.47522900
F	2.18221000	-1.52252600	0.57312400

2. IM1 ($C_6F_{13}\cdot$)

EE[B3LYP/6−311G**] = -1526.91161 Ha

$Hcorr^{\theta}$ [B3LYP/6−31+G(d,p)]= 0.09114 Ha

$Gcorr^{\theta}$[B3LYP/6−31+G(d,p)]= 0.02535 Ha

H^{θ}= *EE*+ $Hcorr^{\theta}$= -1526.82047 Ha

G^{θ}= *EE*+ $Gcorr^{\theta}$= -1526.88627 Ha

$Hcorr^{r}$[B3LYP/6−31+G(d,p)]= 0.56795 Ha

$Gcorr^{r}$[B3LYP/6−31+G(d,p)]= -1.23274 Ha

H^{r}= *EE*+ $Hcorr^{r}$= -1526.34367 Ha

G^{r}= *EE*+ $Gcorr^{r}$= -1528.14435 Ha

C	2.16213900	0.66431700	-0.00006400
C	3.50398800	-0.04670000	-0.00001700
F	2.10232600	1.44105600	-1.10831100
F	2.10235400	1.44125700	1.10804500
F	3.77300300	-0.73548000	-1.10097700
F	3.77305200	-0.73524900	1.10107500
C	-0.45971800	0.39937600	0.00001500
C	0.93328900	-0.29650300	0.00003700
F	-0.54759800	1.17879200	-1.10366200
F	-0.54762600	1.17880600	1.10368100

F	1.02954900	-1.07771800	1.10335600
F	1.02949900	-1.07788300	-1.10317000
F	-3.21251700	0.87772700	-1.09010400
C	-3.06250200	0.11642200	0.00000000
C	-1.67172400	-0.57596100	0.00000200
F	-3.21256600	0.87763900	1.09015900
F	-4.00875600	-0.83051700	-0.00005900
F	-1.60885700	-1.35621100	1.10330300
F	-1.60884400	-1.35618600	-1.10331700

3. IM2 (C_2F_5·)

EE[B3LYP/6−311G**] = -575.51509 Ha

$Hcorr^{\theta}$ [B3LYP/6−31+G(d,p)]= 0.03170 Ha

$Gcorr^{\theta}$[B3LYP/6−31+G(d,p)]= -0.00731 Ha

H^{θ}= *EE*+ $Hcorr^{\theta}$= -575.48339 Ha

G^{θ}= *EE*+ Gcorr^{θ}= -575.52239 Ha

$Hcorr^{r}$[B3LYP/6−31+G(d,p)]= 0.19982 Ha

$Gcorr^{r}$[B3LYP/6−31+G(d,p)]= -0.62748 Ha

H^{r}= *EE*+ $Hcorr^{r}$= -575.31527 Ha

G^{r}= *EE*+ Gcorr^{r}= -576.14257 Ha

F	1.18955400	-1.09295000	-0.53133100
C	0.60731200	0.00000000	-0.01991100
C	-0.86761300	0.00000000	-0.36505700
F	0.81410600	0.00000300	1.31369700
F	1.18955500	1.09294700	-0.53133500
F	-1.50984100	1.10200000	0.00280800
F	-1.50984100	-1.10200000	0.00280800

4. IM3 ($C_6F_{13}OO$·)

EE[B3LYP/6−311G**] = -1677.32354 Ha

$Hcorr^{\theta}$ [B3LYP/6−31+G(d,p)]= 0.10191 Ha

$Gcorr^{\theta}$[B3LYP/6−31+G(d,p)]= 0.03133 Ha

H^{θ}= *EE*+ $Hcorr^{\theta}$= -1677.22164 Ha

G^{θ}= *EE*+ $Gcorr^{\theta}$= -1677.29222 Ha

$Hcorr^{r}$[B3LYP/6−31+G(d,p)]= 0.64144 Ha

$Gcorr^{r}$[B3LYP/6−31+G(d,p)]= -1.34625 Ha

H^{r}= *EE*+ $Hcorr^{r}$= -1676.68210 Ha

G^{r}= *EE*+ $Gcorr^{r}$= -1678.66979 Ha

C	1.74362200	-0.33407600	0.26260200
C	3.04852900	0.40040700	-0.16587800
F	1.81662800	-0.56772100	1.59067400
F	1.67789500	-1.50919700	-0.40029900
F	3.04708000	1.65324600	0.30124900
F	3.17621800	0.39614400	-1.49119700
O	4.20662900	-0.19551500	0.43860300
O	4.49515100	-1.36373600	-0.12915100
C	-0.85293400	-0.36594200	0.03235400
C	0.45025300	0.48829500	-0.02453400
F	-0.78608000	-1.19841100	1.09724700
F	-0.92657300	-1.09965900	-1.10232900
F	0.54844800	1.03206600	-1.26031500
F	0.36914100	1.47948400	0.89265000
F	-3.48813500	-1.44398300	0.52775400
C	-3.45582100	-0.29039700	-0.15338100
C	-2.14849400	0.48584500	0.16368500
F	-3.54364100	-0.54937600	-1.46181600
F	-4.50320900	0.45818100	0.21051500
F	-2.07691900	1.53682900	-0.68747400
F	-2.23587200	0.94231000	1.43170700

5. TS1

EE[B3LYP/6−311G**] = -1677.22176 Ha

$Hcorr^{\theta}$ [B3LYP/6−31+G(d,p)]= 0.09956 Ha

$Gcorr^{\theta}$[B3LYP/6−31+G(d,p)]= 0.02369 Ha

H^{θ}= EE+ $Hcorr^{\theta}$= -1677.12221 Ha

$\boldsymbol{G}^{\theta}$= EE+ Gcorr$^{\theta}$= -1677.19807 Ha

$Hcorr^{\mathrm{r}}$[B3LYP/6−31+G(d,p)]= 0.63100 Ha

$\boldsymbol{Gcorr}^{\mathrm{r}}$[B3LYP/6−31+G(d,p)]= -1.35270 Ha

H^{r}= EE+ $Hcorr^{\mathrm{r}}$= -1676.59077 Ha

$\boldsymbol{G}^{\mathrm{r}}$= EE+ Gcorr^{r}= -1678.57446 Ha

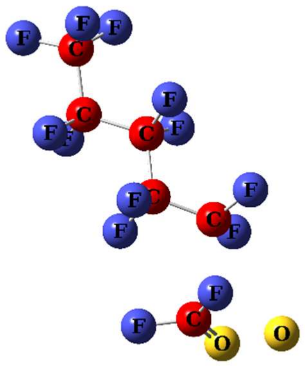

C	1.54022400	0.90190000	-0.00098900
C	3.61563300	-0.86783500	0.11590400
F	1.68217100	1.65583700	-1.07070800
F	1.57400700	1.59121600	1.12695600
F	3.14104800	-2.03396800	-0.20999400
F	3.62505000	-0.62277400	1.38347200
O	4.17312600	-0.16659800	-0.74741300
O	4.65975900	1.08403700	-0.31124200
C	-1.00426000	0.47904100	0.07595000
C	0.42129700	-0.12093500	-0.07637400
F	-1.07796700	1.57813400	-0.71233500
F	-1.15742900	0.85285000	1.36810900
F	0.61497500	-1.02200200	0.92124100
F	0.51162600	-0.74937900	-1.27314300
F	-3.76924800	1.24852500	-0.18477500
C	-3.55583300	-0.02830800	0.16044600
C	-2.15365300	-0.48431200	-0.31843100
F	-3.65821200	-0.14834000	1.48780300

F	-4.48518900	-0.79693900	-0.41763000
F	-1.91030200	-1.70456700	0.21563600
F	-2.18425600	-0.58379400	-1.66460900

6. **IM4 (C_5F_{11})**

EE[B3LYP/6−311G**] = -1289.06381 Ha

$Hcorr^{\theta}$ [B3LYP/6−31+G(d,p)]= 0.07607 Ha

$\boldsymbol{G}corr^{\theta}$[B3LYP/6−31+G(d,p)]= 0.01897 Ha

$H^{\theta}= EE+ Hcorr^{\theta}$= -1288.98774 Ha

$\boldsymbol{G}^{\theta}= EE+ \mathrm{Gcorr}^{\theta}$= -1289.04484 Ha

$Hcorr^{\mathrm{r}}$[B3LYP/6−31+G(d,p)]= 0.47331 Ha

$\boldsymbol{G}corr^{\mathrm{r}}$[B3LYP/6−31+G(d,p)]= -1.07249 Ha

$H^{\mathrm{r}}= EE+ Hcorr^{\mathrm{r}}$= -1288.59050 Ha

$\boldsymbol{G}^{\mathrm{r}}= EE+ \mathrm{Gcorr}^{\mathrm{r}}$= -1290.13630 Ha

C	-2.83284400	-0.15781400	-0.00002200
F	-3.05810900	-0.86223300	-1.10104700
F	-3.05815200	-0.86223900	1.10099200
C	-0.25186200	-0.24863000	0.00003100
C	-1.53646600	0.63283300	0.00000600
F	-0.29594100	-1.03636800	-1.10310200
F	-0.29592400	-1.03628300	1.10322500
F	-1.52233900	1.41196900	1.10820000
F	-1.52229200	1.41196800	-1.10818800
F	2.39164300	-1.14961100	-1.09011700
C	2.36231800	-0.37377700	0.00000000
C	1.09488600	0.52326300	-0.00001400
F	2.39171800	-1.14947700	1.09021000
F	3.44460600	0.41391600	-0.00008500
F	1.15040200	1.30393000	1.10345100
F	1.15036800	1.30384500	-1.10354100

7. **IM5 (CF_2O_2(S1))**

EE[B3LYP/6−311G**] = -388.15706 Ha

$Hcorr^{\theta}$ [B3LYP/6−31+G(d,p)]= 0.02175 Ha

$Gcorr^{\theta}$[B3LYP/6−31+G(d,p)]= -0.01134 Ha

H^{θ}= *EE*+ $Hcorr^{\theta}$= -388.13531 Ha

G^{θ}= *EE*+ Gcorr^{θ}= -388.16840 Ha

$Hcorr^{r}$[B3LYP/6−31+G(d,p)]= 0.13683 Ha

$Gcorr^{r}$[B3LYP/6−31+G(d,p)]= -0.51380 Ha

H^{r}= *EE*+ $Hcorr^{r}$= -388.02024 Ha

G^{r}= *EE*+ Gcorr^{r}= -388.67086 Ha

C	-0.38866400	-0.02861400	0.00003200
F	-1.55065300	-0.61217900	-0.00000800
F	-0.43399200	1.25985600	-0.00000600
O	0.64742600	-0.70447500	-0.00000500
O	1.87679800	-0.00270100	-0.0000030

8. IM6 ($:CF_2$(S1))

EE[B3LYP/6−311G**] = -237.76499 Ha

$Hcorr^{\theta}$ [B3LYP/6−31+G(d,p)]= 0.01082 Ha

$Gcorr^{\theta}$[B3LYP/6−31+G(d,p)]= -0.01769 Ha

H^{θ}= *EE*+ $Hcorr^{\theta}$= -237.75418 Ha

G^{θ}= *EE*+ Gcorr^{θ}= -237.78269 Ha

$Hcorr^{r}$[B3LYP/6−31+G(d,p)]= 0.07356 Ha

$Gcorr^{r}$[B3LYP/6−31+G(d,p)]= -0.38575 Ha

H^{r}= *EE*+ $Hcorr^{r}$= -237.69143 Ha

G^{r}= *EE*+ Gcorr^{r}= -238.15075 Ha

F	0.00000000	1.03551600	-0.20198300
C	0.00000000	0.00000000	0.60594800
F	0.00000000	-1.03551600	-0.20198300

9. IM7 ($\cdot CF_3$)

EE[B3LYP/6−311G**] = -337.65773 Ha

$Hcorr^{\theta}$ [B3LYP/6−31+G(d,p)]= 0.01656 Ha

$Gcorr^{\theta}$[B3LYP/6−31+G(d,p)]= -0.01457 Ha

H^{θ}= EE+ $Hcorr^{\theta}$= -337.64117 Ha

G^{θ}= EE+ Gcorr^{θ}= -337.67229 Ha

$Hcorr^{\mathrm{r}}$[B3LYP/6−31+G(d,p)]= 0.10517 Ha

$Gcorr^{\mathrm{r}}$[B3LYP/6−31+G(d,p)]= -0.44658 Ha

H^{r}= EE+ $Hcorr^{\mathrm{r}}$= -337.55256 Ha

G^{r}= EE+ $\mathrm{Gcorr}^{\mathrm{r}}$= -338.10431 Ha

F	-1.13796800	-0.55140800	-0.07309300
C	-0.00008700	0.00005400	0.32888300
F	1.04659100	-0.70972600	-0.07307100
F	0.09143500	1.26109800	-0.07309200

10. **TS2**

EE[B3LYP/6−311G**] = -1289.03346 Ha

$Hcorr^{\theta}$ [B3LYP/6−31+G(d,p)]= 0.07481 Ha

$Gcorr^{\theta}$[B3LYP/6−31+G(d,p)]= - 0.01135 Ha

H^{θ}= EE+ $Hcorr^{\theta}$= -1288.95865 Ha

G^{θ}= EE+ Gcorr^{θ}= -1289.02212 Ha

$Hcorr^{\mathrm{r}}$[B3LYP/6−31+G(d,p)]= 0.47334 Ha

$Gcorr^{\mathrm{r}}$[B3LYP/6−31+G(d,p)]= -1.10448 Ha

H^{r}= EE+ $Hcorr^{\mathrm{r}}$= -1288.56012 Ha

G^{r}= EE+ $\mathrm{Gcorr}^{\mathrm{r}}$= -1290.13794 Ha

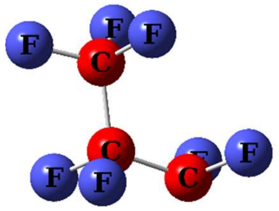

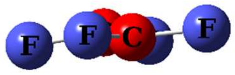

C	-2.52977200	-0.71080400	-0.12840400
F	-2.96846200	-0.73474400	-1.37143300
F	-2.24939200	-1.89900200	0.36686300
C	0.04345400	0.69605900	-0.47074700
C	-2.15000900	0.43451300	0.47494100
F	0.32081000	1.99652600	-0.53340900
F	-0.07528900	0.16829400	-1.69424600
F	-1.78447900	0.45253800	1.74914000
F	-2.53910200	1.61624200	0.01405800
F	2.94863600	0.96634400	-0.37405500
C	2.42998500	-0.24475100	-0.12016500
C	0.98834200	-0.08707300	0.41898000
F	2.42001600	-0.95713600	-1.25462600
F	3.19988000	-0.86833900	0.77845900
F	0.47585700	-1.33439200	0.58437500
F	1.06352400	0.53504100	1.61847000

11. **IM8 (C_3F_7)**

EE[B3LYP/6−311G**] = -813.36641 Ha

$Hcorr^{\theta}$ [B3LYP/6−31+G(d,p)]= 0.04685 Ha

$Gcorr^{\theta}$[B3LYP/6−31+G(d,p)]= 0.00035 Ha

H^{θ}= *EE*+ $Hcorr^{\theta}$= -813.31956 Ha

G^{θ}= *EE*+ $Gcorr^{\theta}$= -813.36606 Ha

$Hcorr^{r}$[B3LYP/6−31+G(d,p)]= 0.29448 Ha

$Gcorr^{r}$[B3LYP/6−31+G(d,p)]= -0.78998 Ha

H^{r}= EE+ $Hcorr^{r}$= -813.07193 Ha

G^{r}= EE+ $Gcorr^{r}$= -814.15639 Ha

C	-1.52236200	0.13723600	0.00000000
F	-1.85446300	-0.52621200	-1.10065400
F	-1.85446200	-0.52621500	1.10065400
F	0.89816400	-1.16076500	-1.08959600
C	1.01127100	-0.38762000	-0.00000100
C	-0.10855700	0.68415400	0.00000100
F	0.89816600	-1.16076500	1.08959500
F	2.21654500	0.19204700	-0.00000100
F	0.05457500	1.44636400	1.10791200
F	0.05457400	1.44636600	-1.10790900

12. **PT1 (C_2F_4)**

EE[B3LYP/6−311G**] = -475.64811 Ha

$Hcorr^{\theta}$ [B3LYP/6−31+G(d,p)]= 0.02774 Ha

$Gcorr^{\theta}$[B3LYP/6−31+G(d,p)]= -0.00772 Ha

H^{θ}= EE+ $Hcorr^{\theta}$= -475.62036 Ha

G^{θ}= EE+ $Gcorr^{\theta}$= -475.65582 Ha

$Hcorr^{r}$[B3LYP/6−31+G(d,p)]= 0.16856 Ha

$Gcorr^{r}$[B3LYP/6−31+G(d,p)]= -0.55524 Ha

H^{r}= EE+ $Hcorr^{r}$= -475.47955 Ha

G^{r}= EE+ $Gcorr^{r}$= -476.20334 Ha

C	0.66276500	0.00000000	0.00007700
C	-0.66276600	0.00000000	-0.00000700
F	1.38677900	1.10877800	-0.00004400
F	1.38678100	-1.10877700	0.00000700
F	-1.38678000	1.10877700	0.00002100
F	-1.38677900	-1.10877800	-0.00003000

13. **TS3**

EE[B3LYP/6−311G**] = -813.31310 Ha

$Hcorr^{\theta}$ [B3LYP/6−31+G(d,p)]= 0.04444 Ha

$Gcorr^{\theta}$[B3LYP/6−31+G(d,p)]= -0.00631 Ha

H^{θ}= *EE*+ $Hcorr^{\theta}$= -813.26866 Ha

G^{θ}= *EE*+ $Gcorr^{\theta}$= -813.31941 Ha

$Hcorr^{r}$[B3LYP/6−31+G(d,p)]= 0.28403 Ha

$Gcorr^{r}$[B3LYP/6−31+G(d,p)]= -0.79810 Ha

H^{r}= *EE*+ $Hcorr^{r}$= -813.02907 Ha

G^{r}= *EE*+ $Gcorr^{r}$= - -814.11120 Ha

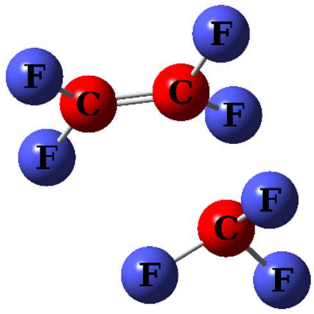

C	-1.55804400	-0.26633100	-0.00003900
F	-1.88235100	-0.90649300	-1.10707100
F	-1.88241300	-0.90646600	1.10699000
F	1.22935100	-1.58493500	0.00012700
C	1.42237900	-0.26514500	0.00004100
C	-0.76030100	0.81638300	-0.00002900
F	2.07764600	0.10909500	1.09456200
F	2.07764200	0.10895400	-1.09453000
F	-0.51131200	1.49496500	1.11068500
F	-0.51125100	1.49494100	-1.11074400

14. **IM12 (CF_2O_2(S3))**

EE[B3LYP/6−311G**] = -388.12994 Ha

$Hcorr^{\theta}$ [B3LYP/6−31+G(d,p)]= 0.02053 Ha

$Gcorr^{\theta}$[B3LYP/6−31+G(d,p)]= -0.01417 Ha

H^{θ}= EE+ $Hcorr^{\theta}$= -388.10941 Ha

G^{θ}= EE+ $Gcorr^{\theta}$= -388.14411 Ha

$Hcorr^{r}$[B3LYP/6−31+G(d,p)]= 0.13663 Ha

$Gcorr^{r}$[B3LYP/6−31+G(d,p)]= -0.52568 Ha

H^{r}= EE+ $Hcorr^{r}$= -387.99331 Ha

G^{r}= EE+ $Gcorr^{r}$= -388.65561 Ha

C	0.38766700	0.00724600	0.27919600
F	0.69500500	1.23936000	-0.09432700
F	1.37735700	-0.83714600	0.05887500
O	-0.76319400	-0.47932100	-0.37761000
O	-1.85896300	0.02139600	0.20809600

15. **TS4**

EE[B3LYP/6−311G**] = - 388.11340 Ha

$Hcorr^{\theta}$ [B3LYP/6−31+G(d,p)]= 0.01860 Ha

$Gcorr^{\theta}$[B3LYP/6−31+G(d,p)]= -0.01605 Ha

H^{θ}= EE+ $Hcorr^{\theta}$= - 388.09481 Ha

G^{θ}= EE+ $Gcorr^{\theta}$= -388.12945 Ha

$Hcorr^{r}$[B3LYP/6−31+G(d,p)]= 0.12608 Ha

$Gcorr^{r}$[B3LYP/6−31+G(d,p)]= -0.52180 Ha

H^{r}= EE+ $Hcorr^{r}$= -387.98732 Ha

G^{r}= EE+ $Gcorr^{r}$= -388.63520 Ha

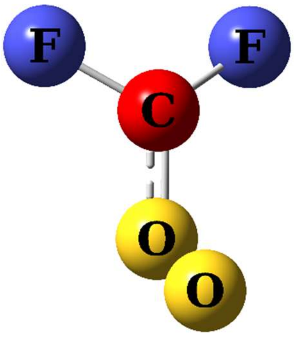

C	-0.34612100	-0.00002900	-0.06520900
F	-1.07727300	1.10220500	-0.08858500
F	-1.07740500	-1.10207700	-0.08870500

O	0.75994200	-0.00018700	0.60973400
O	1.92366200	0.00006500	-0.36137600

16. **PT2 (CF_2O)**

EE[B3LYP/6−311G**] = -313.10633 Ha

$Hcorr^{\theta}$ [B3LYP/6−31+G(d,p)]= 0.01838 Ha

$Gcorr^{\theta}$[B3LYP/6−31+G(d,p)]= -0.01170 Ha

H^{θ}= *EE*+ $Hcorr^{\theta}$= -313.08795 Ha

G^{θ}= *EE*+ $Gcorr^{\theta}$= -313.11803 Ha

$Hcorr^{r}$[B3LYP/6−31+G(d,p)]= 0.10552 Ha

$Gcorr^{r}$[B3LYP/6−31+G(d,p)]= -0.43339 Ha

H^{r}= *EE*+ $Hcorr^{r}$= -313.00081 Ha

G^{r}= *EE*+ $Gcorr^{r}$= - 313.53973 Ha

C	0.14302800	-0.00050000	0.00004200
F	-0.64141100	-1.06552800	-0.00001100
F	-0.63019400	1.07184800	-0.00000800
O	1.32328500	-0.00673500	-0.00001000

The steam atmosphere (Supplementary Fig.2, Fig.S3)

1. INT (C_8F_{18})

EE[B3LYP/6−311G**] = -2102.55315 Ha

$Hcorr^{\theta}$ [B3LYP/6−31+G(d,p)]= 0.12786 Ha

$Gcorr^{\theta}$[B3LYP/6−31+G(d,p)]= 0.04819 Ha

H^{θ}= *EE*+ $Hcorr^{\theta}$= -2102.4252 Ha

G^{θ}= *EE*+ Gcorr$^{\theta}$= -2102.50495 Ha

$Hcorr^{r}$[B3LYP/6−31+G(d,p)]= 0.79927 Ha

$Gcorr^{r}$[B3LYP/6−31+G(d,p)]= -1.58057 Ha

H^{r}= *EE*+ $Hcorr^{r}$= -2101.75387 Ha

G^{r}= *EE*+ Gcorr^{r}= -2104.13372 Ha

C	-2.93993000	-0.68497300	-0.10424800
C	-4.41141100	-0.23476400	0.10573100
F	-2.85949400	-1.28229000	-1.31298900
F	-2.64414800	-1.58652700	0.86198900
F	-5.22309800	-1.25839400	-0.18341800
F	-4.70351200	0.79324400	-0.70282700
F	-4.61492900	0.13234800	1.37486100
C	-0.43258200	-0.01104500	0.11845200
C	-1.90479300	0.47624000	-0.05588500
F	-0.23097400	-1.08780500	-0.68009400
F	-0.26118900	-0.37360900	1.40944800
F	-2.20787400	1.28530100	0.98584400
F	-2.00802200	1.17802000	-1.20800800
C	2.08823200	0.81116400	0.24661700
C	0.62691800	1.06601200	-0.25212000
F	2.10357000	0.90523600	1.59359200
F	2.83407400	1.81927100	-0.27127500
F	0.24781400	2.24395700	0.30265100
F	0.65730100	1.20062200	-1.59478100
F	4.68916500	-1.85717600	-0.12070300
C	4.27876600	-0.59588400	0.05636000

C	2.74243200	-0.53450200	-0.16324400
F	4.59006700	-0.20841500	1.30022300
F	4.91052900	0.19096200	-0.82018400
F	2.50675700	-0.76704800	-1.47522900
F	2.18221000	-1.52252600	0.57312400

2. TS1

EE[B3LYP/6−311G**] = -2103.01432 Ha

$Hcorr^{\theta}$ [B3LYP/6−31+G(d,p)]= 0.12959 Ha

$Gcorr^{\theta}$[B3LYP/6−31+G(d,p)]= 0.04761 Ha

H^{θ}= *EE*+ $Hcorr^{\theta}$= -2102.88473 Ha

G^{θ}= *EE*+ $Gcorr^{\theta}$= -2102.96670 Ha

$Hcorr^{r}$[B3LYP/6−31+G(d,p)]= 0.82013 Ha

$Gcorr^{r}$[B3LYP/6−31+G(d,p)]= -1.63688 Ha

H^{r}= *EE*+ $Hcorr^{r}$= -2102.19419 Ha

G^{r}= *EE*+ $Gcorr^{r}$= -2104.65120 Ha

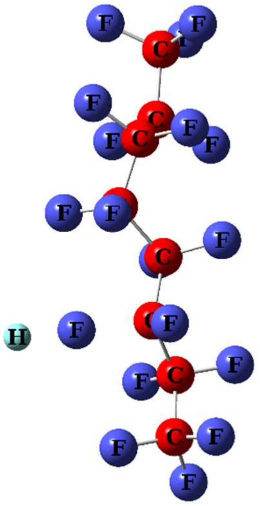

C	-2.91880600	-0.70450300	-0.12953500
C	-4.38172200	-0.24440100	0.10057800
F	-2.81713200	-1.16136500	-1.39497200
F	-2.68737600	-1.74176900	0.72585500
F	-5.20694500	-1.22140000	-0.29198700
F	-4.64782100	0.85943600	-0.60836400
F	-4.59379700	0.00550400	1.39797200

C	-0.41531400	-0.06365800	0.16603500
C	-1.87536700	0.37914700	0.11611300
F	-0.22193200	-1.06777800	-0.72052700
F	-0.17524600	-0.53010200	1.42318700
F	-2.18395900	1.15892400	1.15062000
F	-2.00247700	1.35091400	-1.22965300
C	2.08541700	0.78438500	0.31795100
C	0.61771800	1.05626200	-0.14177600
F	2.12640000	0.79051300	1.66698300
F	2.81654000	1.82948300	-0.14551300
F	0.23015000	2.18540300	0.49980900
F	0.63802400	1.28589200	-1.47067000
F	4.69943600	-1.82217800	-0.27061600
C	4.27804100	-0.57885600	-0.01200200
C	2.73869800	-0.52411700	-0.19295600
F	4.61219000	-0.25936300	1.24513600
F	4.88009600	0.26547900	-0.85513500
F	2.47512800	-0.67277900	-1.51161000
F	2.20715100	-1.56470900	0.48860000
H	-2.10787500	2.16348300	-2.23848000

3. **IM1 (C_8F_{17})**

EE[B3LYP/6−311G**] = -2002.62547 Ha

$Hcorr^{\theta}$ [B3LYP/6−31+G(d,p)]= 0.12264 Ha

$Gcorr^{\theta}$[B3LYP/6−31+G(d,p)]= 0.04404 Ha

H^{θ}= *EE*+ $Hcorr^{\theta}$= -2002.50283 Ha

G^{θ}= *EE*+ $Gcorr^{\theta}$= -2002.58143 Ha

$Hcorr^{r}$[B3LYP/6−31+G(d,p)]= 0.76772 Ha

$Gcorr^{r}$[B3LYP/6−31+G(d,p)]= -1.54141 Ha

H^{r}= *EE*+ $Hcorr^{r}$= -2001.85774 Ha

G^{r}= *EE*+ $Gcorr^{r}$= -2004.16687 Ha

C	2.98495500	-0.47985500	0.61664700

C	3.72012700	0.80111800	0.14096200
F	2.34517600	-0.18419500	1.77271500
F	3.94997900	-1.40929300	0.87156500
F	4.48124300	1.28311300	1.12736900
F	2.82141500	1.73366900	-0.21274200
F	4.49448100	0.52528800	-0.91517000
C	0.50779400	-1.01971700	-0.25224500
C	2.00529500	-1.02994300	-0.37336500
F	0.14963100	-1.46455600	0.97907500
F	0.00470100	-1.85461000	-1.19799800
F	2.47490000	-1.19011100	-1.60567300
C	-1.66715600	0.45312600	-0.67779200
C	-0.12604200	0.38194900	-0.46710800
F	-1.96222500	-0.11341700	-1.86708100
F	-1.98107600	1.77229400	-0.73002000
F	0.43262400	0.90351600	-1.59002300
F	0.20260400	1.16216900	0.58613400
F	-4.72691200	-0.61373800	1.18404000
C	-4.03876000	0.18304500	0.35835800
C	-2.54022600	-0.21133400	0.41452600
F	-4.51374100	0.03461100	-0.88529400
F	-4.20472400	1.45183800	0.74404000
F	-2.07037800	0.13144100	1.63585300
F	-2.46169000	-1.55361000	0.26322200

4. TS2

EE[B3LYP/6−311G**] = -2003.04667 Ha

$Hcorr^{\theta}$ [B3LYP/6−31+G(d,p)]= 0.12381 Ha

$Gcorr^{\theta}$[B3LYP/6−31+G(d,p)]= 0.04456 Ha

$H^{\theta}= EE+ Hcorr^{\theta}$= -2002.92286 Ha

$G^{\theta}= EE+$ Gcorr$^{\theta}$= -2003.00211 Ha

$Hcorr^{r}$[B3LYP/6−31+G(d,p)]= 0.78848 Ha

$Gcorr^{r}$[B3LYP/6−31+G(d,p)]= -1.58331 Ha

$H^r = EE + Hcorr^r$ = -2002.25819 Ha

$\boldsymbol{G}^r = EE+$ Gcorr^r= -2004.62998 Ha

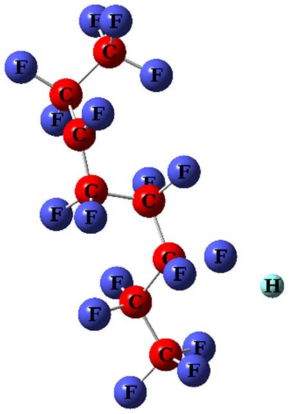

C	2.98313500	0.55719400	-0.54168500
C	3.70659100	-0.79067600	-0.26698800
F	2.34587800	0.44174800	-1.73293900
F	3.98007400	1.48952200	-0.66956200
F	4.45917900	-1.13009300	-1.31983800
F	2.80358700	-1.76186200	-0.05236700
F	4.49124800	-0.68875900	0.81294500
C	0.56494500	1.02535900	0.39653000
C	2.02225000	0.97803700	0.51303100
F	0.16943600	1.59283300	-0.77474400
F	0.03857600	1.71993500	1.44587200
F	2.49602400	0.90307100	1.76056800
C	-1.63350300	-0.43553500	0.63256700
C	-0.10927400	-0.40672500	0.44239700
F	-1.96384400	-0.03880600	1.86238100
F	-1.97810200	-1.85291700	0.55163100
F	0.41829700	-1.06958700	1.50187800
F	0.20843600	-1.05787100	-0.69650600
F	-4.69876100	0.78749400	-1.11236600
C	-4.00334200	-0.11435300	-0.41518400
C	-2.50718200	0.30261500	-0.40618800
F	-4.48357600	-0.16576700	0.83526400
F	-4.15210100	-1.31327700	-0.99052100

F	-2.01827500	0.09586000	-1.64624200
F	-2.45958300	1.62200100	-0.11009200
H	-3.05016300	-2.85724300	0.88486900

5. **IM2 (C_8F_{16})**

EE[B3LYP/6−311G**] = -1902.74420 Ha

$Hcorr^{\theta}$ [B3LYP/6−31+G(d,p)]= 0.11687 Ha

$Gcorr^{\theta}$[B3LYP/6−31+G(d,p)]= 0.03719 Ha

H^{θ}= *EE*+ $Hcorr^{\theta}$= -1902.62733 Ha

G^{θ}= *EE*+ Gcorr^{θ}= -1902.70701 Ha

$Hcorr^{r}$[B3LYP/6−31+G(d,p)]= 0.73662 Ha

$Gcorr^{r}$[B3LYP/6−31+G(d,p)]= -1.49564 Ha

H^{r}= *EE*+ $Hcorr^{r}$= -1902.00758 Ha

G^{r}= *EE*+ Gcorr^{r}= -1904.23984 Ha

C	-2.87715100	0.50672600	0.54789400
C	-3.53024500	-0.83589100	0.13330400
F	-2.17405500	0.28414000	1.68620400
F	-3.89931300	1.36281200	0.83611800
F	-4.17695300	-1.36259500	1.17843700
F	-2.58347500	-1.70002600	-0.26905700
F	-4.39900800	-0.65050800	-0.86605600
C	-0.77963700	1.62592100	-0.31204300
C	-1.98563600	1.09204000	-0.50369000
F	-0.16626700	1.69679600	0.84883700
F	-0.06457600	2.13423600	-1.29247400
F	-2.50725400	1.09563200	-1.74488100
C	1.70241500	-0.56080000	-0.66855800
C	0.60269500	-1.25246500	-0.37068100
F	2.15325800	-0.57802100	-1.93651100
F	-0.05886200	-1.93985600	-1.27698900
F	0.04711700	-1.31869300	0.81924800
F	4.69549000	0.45366100	1.25026700

C	4.02845500	-0.22993000	0.31445100
C	2.55244500	0.23797700	0.26940800
F	4.62047000	-0.03754500	-0.86959000
F	4.07383600	-1.53778300	0.61596500
F	2.06531000	0.16314200	1.53171800
F	2.56538800	1.54555500	-0.11795800

6. TS3

EE[B3LYP/6−311G**] = -1902.68711 Ha

$Hcorr^{\theta}$ [B3LYP/6−31+G(d,p)]= 0.11603 Ha

$Gcorr^{\theta}$[B3LYP/6−31+G(d,p)]= 0.03858 Ha

H^{θ}= *EE*+ $Hcorr^{\theta}$= -1902.57108 Ha

G^{θ}= *EE*+ $Gcorr^{\theta}$= -1902.64853 Ha

$Hcorr^{r}$[B3LYP/6−31+G(d,p)]= 0.72577 Ha

$Gcorr^{r}$[B3LYP/6−31+G(d,p)]= -1.48153 Ha

H^{r}= *EE*+ $Hcorr^{r}$= -1901.96135 Ha

G^{r}= *EE*+ $Gcorr^{r}$= -1904.16865 Ha

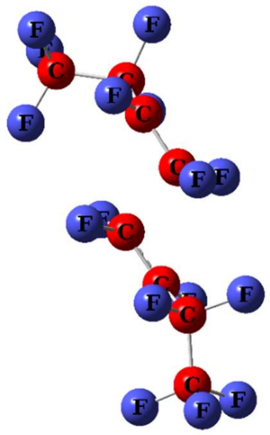

C	2.97381400	0.27544200	0.57542500
C	3.72682800	-0.67436600	-0.39704300
F	3.89630200	1.18706100	1.00691600
F	2.55610100	-0.44540400	1.63967200
F	4.58662000	-1.43046800	0.29281700
F	4.40370900	0.03926400	-1.30436900
F	2.86076300	-1.47387900	-1.03663100
C	0.49733600	1.01471900	0.38389200

C	1.82249400	1.00804800	-0.04470000
F	-0.26335400	1.97455600	-0.17316700
F	0.27080600	0.91257400	1.70224600
F	2.07217100	1.48769700	-1.26187800
C	-1.61260700	-0.51877500	0.24007000
C	-0.31322200	-0.50620500	-0.26788500
F	-1.78517100	-1.05563500	1.44797300
F	0.47264800	-1.49098700	0.19941100
F	-0.15585700	-0.34335200	-1.58886500
F	-5.06416600	0.26141500	-0.75570600
C	-4.12934100	-0.40101000	-0.06895500
C	-2.76385700	0.32749700	-0.20839200
F	-4.49435600	-0.46726100	1.21540400
F	-4.03262500	-1.64814600	-0.55871700
F	-2.60606400	0.67544500	-1.50599400
F	-2.85182300	1.46688800	0.53927900

7. IM3 (C_4F_8)

EE[B3LYP/6−311G**] =-951.37312 Ha

$Hcorr^{\theta}$ [B3LYP/6−31+G(d,p)]= 0.05845 Ha

$Gcorr^{\theta}$[B3LYP/6−31+G(d,p)]= 0.00793 Ha

H^{θ}= *EE*+ $Hcorr^{\theta}$= -951.31467 Ha

G^{θ}= *EE*+ Gcorr$^{\theta}$= -951.36520 Ha

$Hcorr^{r}$[B3LYP/6−31+G(d,p)]= 0.35787 Ha

$Gcorr^{r}$[B3LYP/6−31+G(d,p)]= -0.88188 Ha

H^{r}= *EE*+ $Hcorr^{r}$=-951.01525 Ha

G^{r}= *EE*+ Gcorr^{r}= -952.25501 Ha

C	0.43338000	-0.08620700	0.63871200
C	1.51258900	-0.05272900	-0.46767600
F	0.91565100	0.62810800	1.69047900
F	0.28051100	-1.38042700	1.01560400
F	2.66509300	-0.55932300	-0.01860700

F	1.71681400	1.20859300	-0.87160700
F	1.09647000	-0.78036400	-1.51724600
C	-1.97993900	-0.17936600	-0.11532000
C	-0.87913000	0.49664200	0.20997200
F	-3.08935300	0.41565600	-0.50130600
F	-2.09509600	-1.48915300	-0.08942300
F	-0.88135600	1.83801700	0.11498100

8. IM4 ($\cdot CF_3$)

EE[B3LYP/6−311G**] = -337.65773 Ha

$Hcorr^{\theta}$ [B3LYP/6−31+G(d,p)]= 0.01656 Ha

$Gcorr^{\theta}$[B3LYP/6−31+G(d,p)]= -0.01457 Ha

$H^{\theta}= EE+ Hcorr^{\theta}$= -337.64117 Ha

$G^{\theta}= EE+$ $Gcorr^{\theta}$= -337.67229 Ha

$Hcorr^{r}$[B3LYP/6−31+G(d,p)]= 0.10517 Ha

$Gcorr^{r}$[B3LYP/6−31+G(d,p)]= -0.44658 Ha

$H^{r}= EE+ Hcorr^{r}$= -337.55256 Ha

$G^{r}= EE+$ $Gcorr^{r}$= -338.10431 Ha

F	-1.13796800	-0.55140800	-0.07309300
C	-0.00008700	0.00005400	0.32888300
F	1.04659100	-0.70972600	-0.07307100
F	0.09143500	1.26109800	-0.07309200

9. IM5 (C_3F_5)

EE[B3LYP/6−311G**] = -613.58756 Ha

$Hcorr^{\theta}$ [B3LYP/6−31+G(d,p)]= 0.03690 Ha

$Gcorr^{\theta}$[B3LYP/6−31+G(d,p)]= -0.00587 Ha

$H^{\theta}= EE+ Hcorr^{\theta}$= -613.55066 Ha

$G^{\theta}= EE+$ $Gcorr^{\theta}$= -613.59343 Ha

$Hcorr^{r}$[B3LYP/6−31+G(d,p)]= 0.23154 Ha

$Gcorr^{r}$[B3LYP/6−31+G(d,p)]= -0.69904 Ha

$H^{r}= EE+ Hcorr^{r}$= -613.35602 Ha

$\boldsymbol{G}^{r}$= *EE*+ Gcorr^{r}= -614.28660 Ha

C	1.24068500	-0.14986600	-0.15280900
F	2.35957200	0.50800300	0.11639300
F	1.36715100	-1.46763700	-0.06022500
C	-1.22239600	-0.15437000	0.04166900
C	-0.00418300	0.46769600	-0.00967600
F	-2.36487200	0.50340200	-0.02809700
F	-1.36682800	-1.46747600	0.07207300
F	-0.00442700	1.81473400	-0.01959900

10. **IM6 (:CF_2(S1))**

EE[B3LYP/6−311G**] = -237.76499 Ha

$Hcorr^{\theta}$ [B3LYP/6−31+G(d,p)]= 0.01082 Ha

$Gcorr^{\theta}$[B3LYP/6−31+G(d,p)]= -0.01769 Ha

H^{θ}= *EE*+ $Hcorr^{\theta}$= -237.75418 Ha

$\boldsymbol{G}^{\theta}$= *EE*+ Gcorr$^{\theta}$= -237.78269 Ha

$Hcorr^{r}$[B3LYP/6−31+G(d,p)]= 0.07356 Ha

$Gcorr^{r}$[B3LYP/6−31+G(d,p)]= -0.38575 Ha

H^{r}= *EE*+ $Hcorr^{r}$= -237.69143 Ha

$\boldsymbol{G}^{r}$= *EE*+ Gcorr^{r}= -238.15075 Ha

F	0.00000000	1.03551600	-0.20198300
C	0.00000000	0.00000000	0.60594800
F	0.00000000	-1.03551600	-0.20198300

11. IM7 (·C_2F_3)

EE[B3LYP/6−311G**] = -375.69624 Ha

$Hcorr^{\theta}$ [B3LYP/6−31+G(d,p)]= 0.02231 Ha

$Gcorr^{\theta}$[B3LYP/6−31+G(d,p)]= -0.01185 Ha

H^{θ}= *EE*+ $Hcorr^{\theta}$= -375.67393 Ha

$\boldsymbol{G}^{\theta}$= *EE*+ Gcorr$^{\theta}$= -375.70809 Ha

$Hcorr^{r}$[B3LYP/6−31+G(d,p)]= 0.13694 Ha

$\boldsymbol{Gcorr}^{r}$[B3LYP/6−31+G(d,p)]= -0.52348 Ha

H^{r}= *EE*+ $Hcorr^{r}$= -375.55930 Ha

$\boldsymbol{G}^{r}$= *EE*+ Gcorr^{r}= -376.21972 Ha

C	-0.41917400	-0.06004800	-0.00007700
C	0.77802200	-0.61196100	0.00033100
F	-0.65755600	1.24955600	0.00005300
F	-1.53735800	-0.77006300	-0.00007900
F	1.95568200	-0.03148600	-0.00014300

12. IM8 (·CF_2OH)

EE[B3LYP/6−311G**] = -313.63742 Ha

$Hcorr^{\theta}$ [B3LYP/6−31+G(d,p)]= 0.02723 Ha

$\boldsymbol{Gcorr}^{\theta}$[B3LYP/6−31+G(d,p)]= -0.00390 Ha

H^{θ}= *EE*+ $Hcorr^{\theta}$=-313.61019 Ha

$\boldsymbol{G}^{\theta}$= *EE*+ Gcorr$^{\theta}$=-313.64132 Ha

$Hcorr^{r}$[B3LYP/6−31+G(d,p)]= 0.12809 Ha

$\boldsymbol{Gcorr}^{r}$[B3LYP/6−31+G(d,p)]= -0.45999 Ha

H^{r}= *EE*+ $Hcorr^{r}$= -313.50933 Ha

$\boldsymbol{G}^{r}$= *EE*+ Gcorr^{r}= -314.09741 Ha

C	-0.00402300	-0.00006600	-0.32955100
F	0.66367000	1.09288600	0.07636500
F	0.66353000	-1.09293000	0.07642600
O	-1.30868400	0.00003100	-0.04086500
H	-1.45119200	0.00054600	0.92911200

13. TS4

EE[B3LYP/6−311G**] = -313.57651 Ha

$Hcorr^{\theta}$ [B3LYP/6−31+G(d,p)]= 0.02290 Ha

$\boldsymbol{Gcorr}^{\theta}$[B3LYP/6−31+G(d,p)]= -0.00885 Ha

H^{θ}= *EE*+ $Hcorr^{\theta}$=-313.55361 Ha

$\boldsymbol{G}^{\theta}$= *EE*+ Gcorr$^{\theta}$=-313.58536 Ha

$Hcorr^{r}$[B3LYP/6−31+G(d,p)]= 0.12673 Ha

$\boldsymbol{Gcorr}^{r}$[B3LYP/6−31+G(d,p)]= -0.47696 Ha

$H^{r}= EE+ Hcorr^{r}$= -313.46454 Ha

$\boldsymbol{G}^{r}= EE+$ Gcorr^{r}=-314.07816 Ha

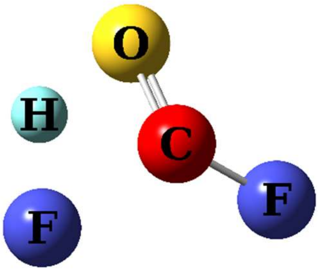

C	0.26084900	0.13573500	0.39388500
F	-1.16126200	-0.74755400	-0.03139700
F	1.31589700	-0.45468600	-0.11901700
O	-0.22092700	1.19522300	-0.09498100
H	-1.18939300	0.44396600	-0.24974200

14. IM9 (·COF)

EE[B3LYP/6−311G**] = -213.16141 Ha

$Hcorr^{\theta}$ [B3LYP/6−31+G(d,p)]= 0.01221 Ha

$\boldsymbol{Gcorr}^{\theta}$[B3LYP/6−31+G(d,p)]= -0.01607 Ha

$H^{\theta}= EE+ Hcorr^{\theta}$=-213.14920 Ha

$\boldsymbol{G}^{\theta}= EE+$ Gcorr$^{\theta}$=-213.17747 Ha

$Hcorr^{r}$[B3LYP/6−31+G(d,p)]= 0.07386 Ha

$\boldsymbol{Gcorr}^{r}$[B3LYP/6−31+G(d,p)]= -0.39225 Ha

$H^{r}= EE+ Hcorr^{r}$= -213.08755 Ha

$\boldsymbol{G}^{r}= EE+$ Gcorr^{r}=-213.55365 Ha

C	0.00000000	0.42598800	0.00000000
F	-1.02182000	-0.43648700	0.00000000
O	1.14954700	0.17155700	0.00000000

15. IM10 (FCOOH)

EE[B3LYP/6−311G**] = -289.097649 Ha

$Hcorr^{\theta}$ [B3LYP/6−31+G(d,p)]= 0.031324 Ha

$Gcorr^{\theta}$[B3LYP/6−31+G(d,p)]= 0.001035 Ha

$H^{\theta}= EE+ Hcorr^{\theta}$= -289.066325 Ha

$G^{\theta}= EE+$ Gcorr$^{\theta}$= -289.096614 Ha

$Hcorr^{r}$[B3LYP/6−31+G(d,p)]= 0.139157 Ha

$Gcorr^{r}$[B3LYP/6−31+G(d,p)]= -0.454417 Ha

$H^{r}= EE+ Hcorr^{r}$= -288.9584923 Ha

$G^{r}= EE+$ Gcorr^{r}= -289.5520665 Ha

C	-0.00982300	0.12522800	-0.00002000
F	-1.11573500	-0.61475000	0.00001500
O	-0.00520100	1.31940400	0.00000400
O	1.03834600	-0.70360800	-0.00001300
H	1.83539000	-0.14498100	0.00005800

16.TS5

EE[B3LYP/6−311G**] = -289.044072Ha

$Hcorr^{\theta}$ [B3LYP/6−31+G(d,p)]= 0.025586Ha

$Gcorr^{\theta}$[B3LYP/6−31+G(d,p)]=- 0.004871Ha

$H^{\theta}= EE+ Hcorr^{\theta}$= -289.018486Ha

$G^{\theta}= EE+$ Gcorr$^{\theta}$= -289.048943Ha

$Hcorr^{r}$[B3LYP/6−31+G(d,p)]= 0.127284Ha

$Gcorr^{r}$[B3LYP/6−31+G(d,p)]= -0.454445Ha

$H^{r}= EE+ Hcorr^{r}$= -288.9167876Ha

$G^{r}= EE+$ Gcorr^{r}= -289.4985167Ha

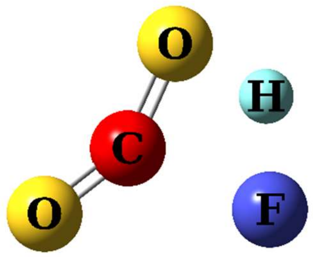

C	-0.00982300	0.12522800	-0.00002000
F	-1.11573500	-0.61475000	0.00001500
O	-0.00520100	1.31940400	0.00000400

O	1.03834600	-0.70360800	-0.00001300
H	1.83539000	-0.14498100	0.00005800

17.IM11(C_2F_3OH)

EE[B3LYP/6−311G**] = -451.633868Ha

$Hcorr^{\theta}$ [B3LYP/6−31+G(d,p)]= 0.040013Ha

$\boldsymbol{Gcorr}^{\theta}$[B3LYP/6−31+G(d,p)]= 0.003913Ha

$H^{\theta}= EE + Hcorr^{\theta}$= -451.593855Ha

$\boldsymbol{G}^{\theta}= EE+$ Gcorr^{θ}= -451.629955Ha

$Hcorr^{r}$[B3LYP/6−31+G(d,p)]= 0.20208Ha

$\boldsymbol{Gcorr}^{r}$[B3LYP/6−31+G(d,p)]= -0.590873Ha

$H^{r}= EE+ Hcorr^{r}$= -451.4317879Ha

$\boldsymbol{G}^{r}= EE+$ Gcorr^{r}= -452.2247408Ha

C	0.66765200	-0.02860700	-0.01126700
C	-0.65988100	0.01145100	0.00547600
F	1.43427000	1.05278000	0.01390300
F	1.36416700	-1.15607600	-0.02437700
F	-1.29538900	1.19893900	-0.01360900
O	-1.44197700	-1.07902600	0.11332700
H	-2.03824000	-1.12564600	-0.65511800

18.IM13 (CFOH)

EE[B3LYP/6−311G**] = -213.758639Ha

$Hcorr^{\theta}$ [B3LYP/6−31+G(d,p)]= 0.023583Ha

$\boldsymbol{Gcorr}^{\theta}$[B3LYP/6−31+G(d,p)]= -0.004583Ha

$H^{\theta}= EE+ Hcorr^{\theta}$= -213.735056Ha

$\boldsymbol{G}^{\theta}= EE+$ Gcorr^{θ}= -213.763222Ha

$Hcorr^{r}$[B3LYP/6−31+G(d,p)]= 0.106958Ha

$\boldsymbol{Gcorr}^{r}$[B3LYP/6−31+G(d,p)]= -0.405484Ha

$H^{r}= EE+ Hcorr^{r}$= -213.6516812Ha

$\boldsymbol{G}^{r}= EE+$ Gcorr^{r}= -214.1641233Ha

C	-0.00886100	0.63815900	-0.00000700
F	1.04914400	-0.19949700	0.00000300
O	-1.06978300	-0.11933700	0.00000700
H	-0.83086000	-1.07878800	-0.00003600

19. TS6

EE[B3LYP/6−311G**] = -213.718433 Ha

$Hcorr^{\theta}$ [B3LYP/6−31+G(d,p)]= 0.018393 Ha

$Gcorr^{\theta}$[B3LYP/6−31+G(d,p)]= -0.009832 Ha

$H^{\theta}= EE+ Hcorr^{\theta}$= -213.70004 Ha

$G^{\theta}= EE+ \mathrm{Gcorr}^{\theta}$= -213.728265 Ha

$Hcorr^{r}$[B3LYP/6−31+G(d,p)]= 0.095302 Ha

$Gcorr^{r}$[B3LYP/6−31+G(d,p)]= -0.403055 Ha

$H^{r}= EE+ Hcorr^{r}$= -213.623131 Ha

$G^{r}= EE+ \mathrm{Gcorr}^{r}$= -214.1214877 Ha

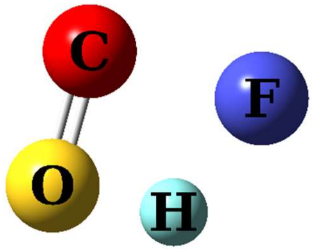

C	0.35768300	0.73110400	-0.00001300
F	-1.10226500	-0.09789500	0.00000600
O	0.97958500	-0.33096900	0.00000200
H	-0.06239100	-0.85781000	0.00001400

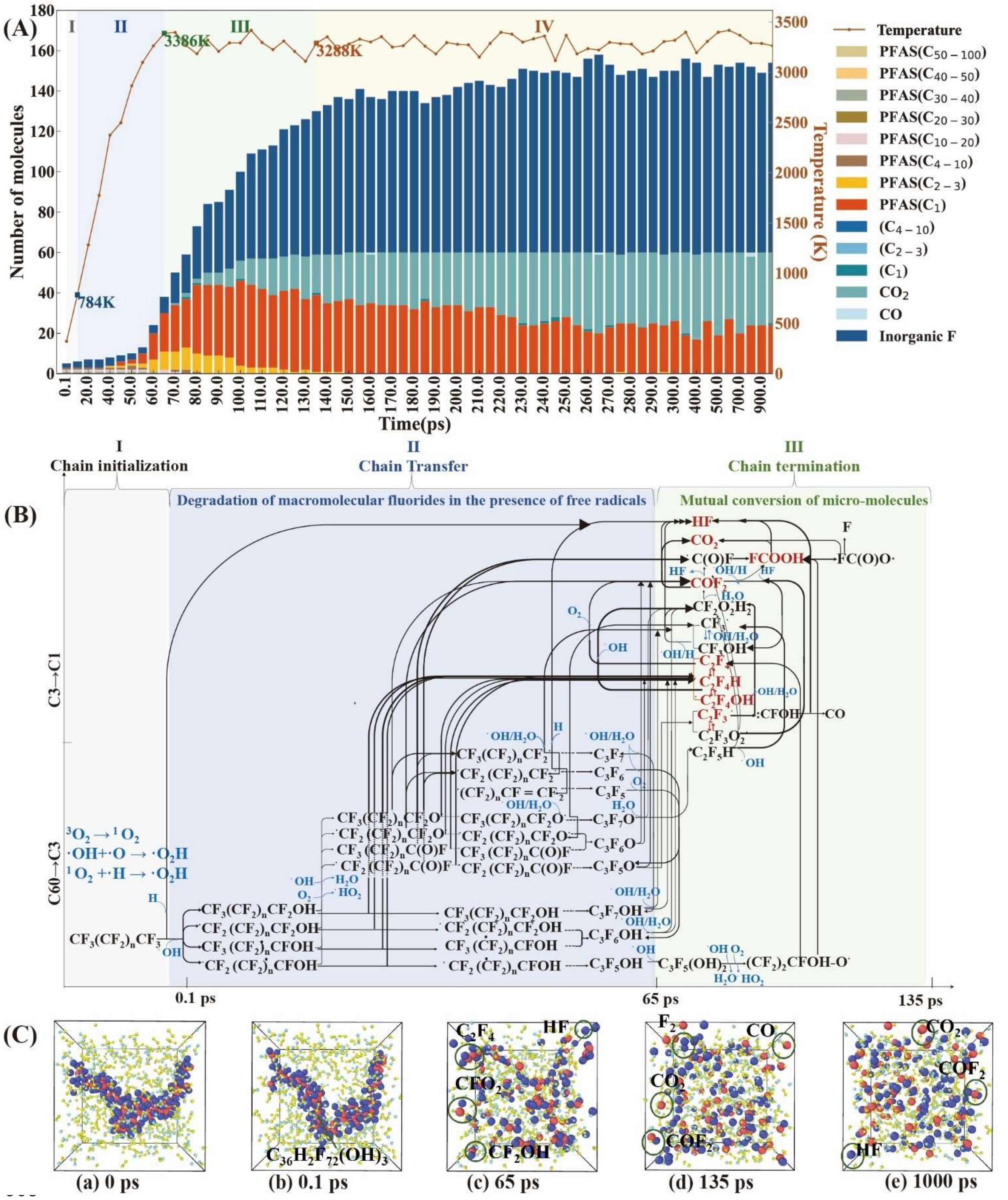

Supplementary Figure 5. MD results of PTFE thermal plasma gasification process under mixed atmosphere (O_2/C ratio=2.79, H_2O/C ratio=2.79, temperature=3300K). (A) The evolution of products distribution with treatment time. (B) The main reaction pathways in (I) - (III) stages. (C) Snapshots of MD trajectory frames at (a) 0ps, (b) 0.1 ps, (c) 65 ps, (d) 135 ps, (e) 1000 ps. The color codes for the atoms are red: C, violet: F, yellow: O, cyan: H.

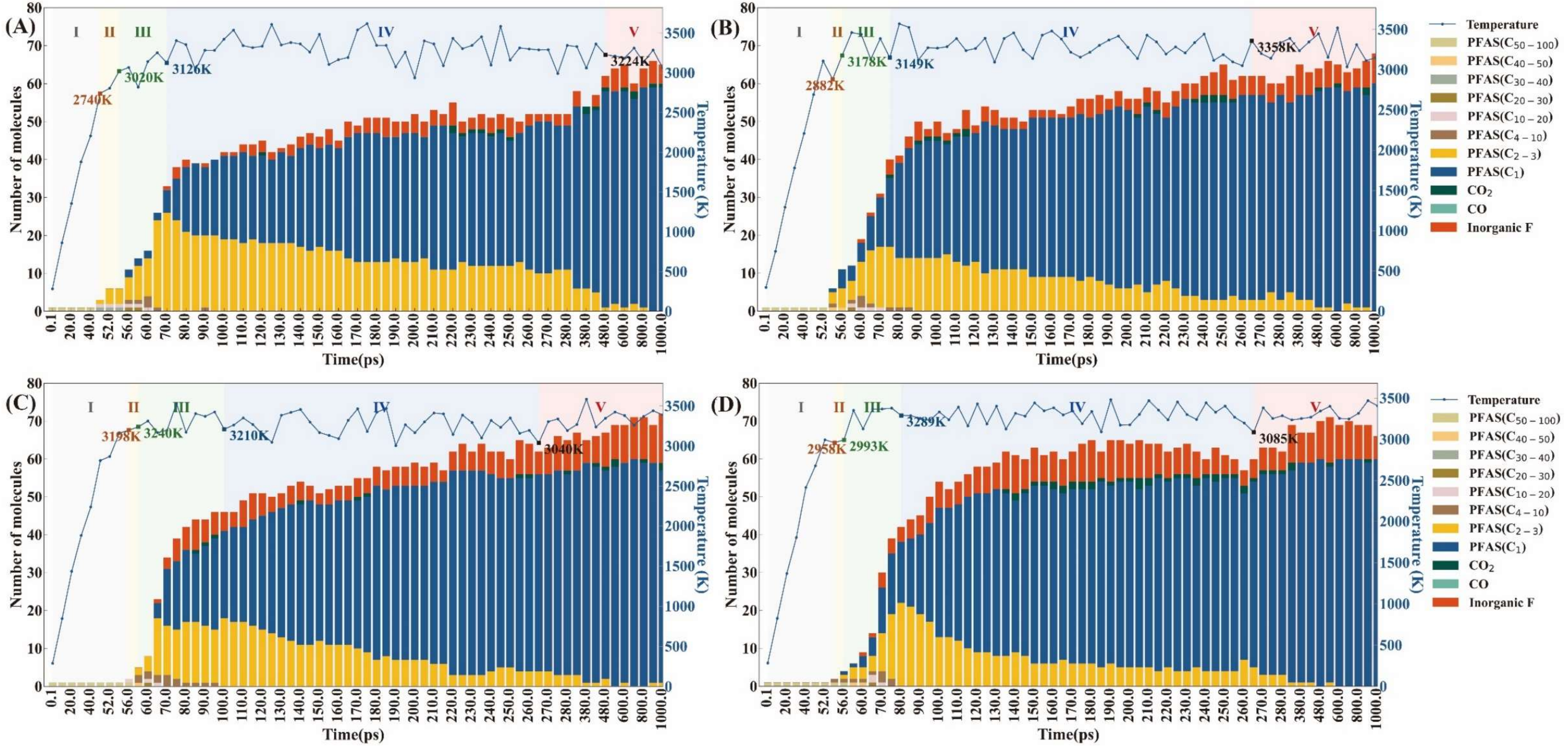

Supplementary Figure 6. MD results of the evolution of product distribution with treatment time during the thermal plasma degradation process of PTFE **at O_2/C ratio of** (A) 0.93, (B)1.86, (C) 3.72, (D) 4.65 (temperature=3300K). The MD result at **O_2/C** ratio of 2.79 was shown in Fig. 4(B).

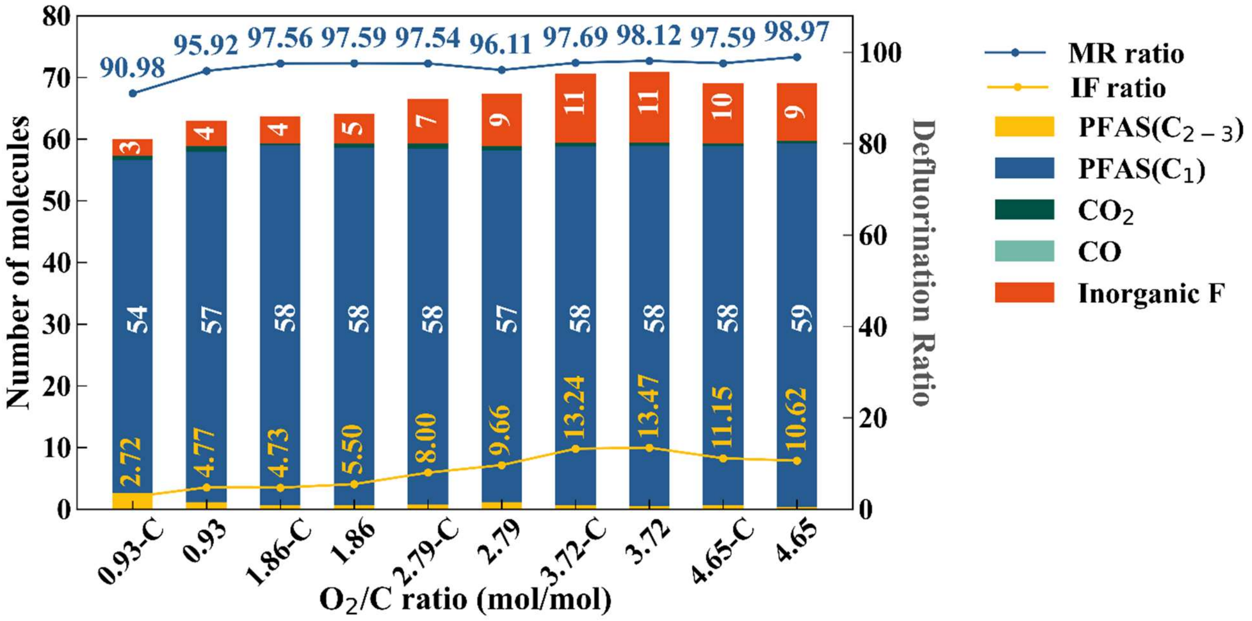

Supplementary Figure 7. The comparison of product composition and defluorination ratio at the equilibrium stage between conventional thermochemical and thermal plasma degradation process **under different O_2/C ratio** (temperature=3300K).

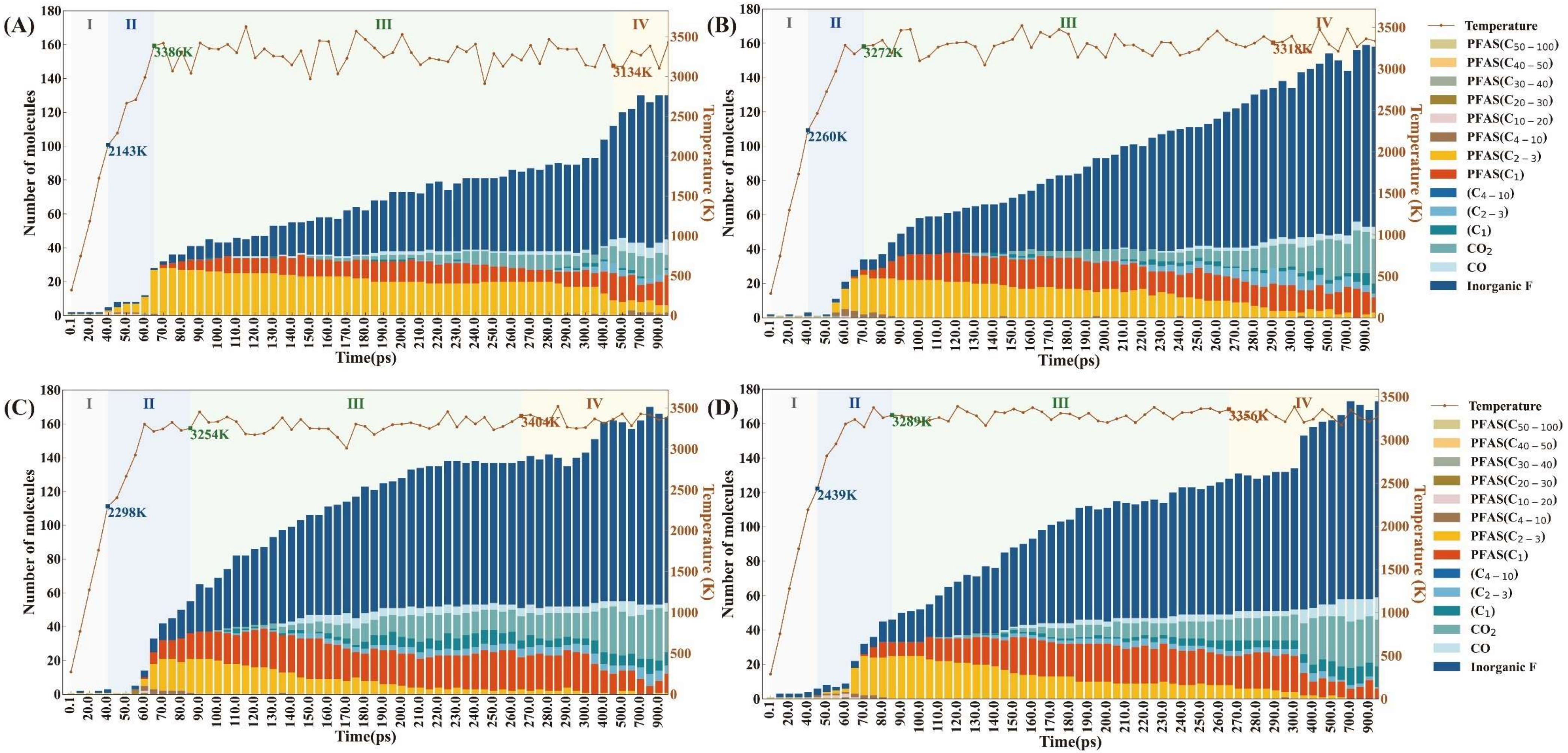

Supplementary Figure 8. MD results of the evolution of product distribution with treatment time during the thermal plasma degradation process of PTFE **at H_2O/C ratio of** (A) 0.93, (B) 1.86, (C)3.72, (D) 4.65 (temperature=3300K). The MD result at H_2O/C ratio of 2.79 was shown in Fig. 5(B).

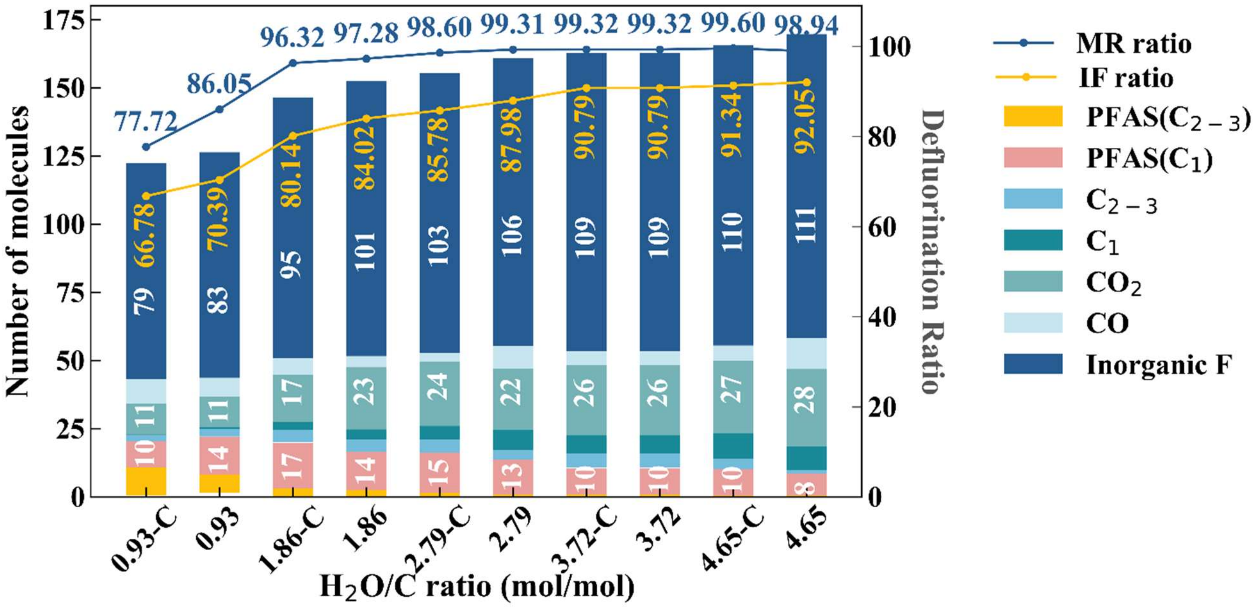

Supplementary Figure 9. The comparison of product composition and Defluorination ratio at the equilibrium stage between conventional thermochemical and thermal plasma degradation process **under different H_2O/C ratio** (temperature=3300K).

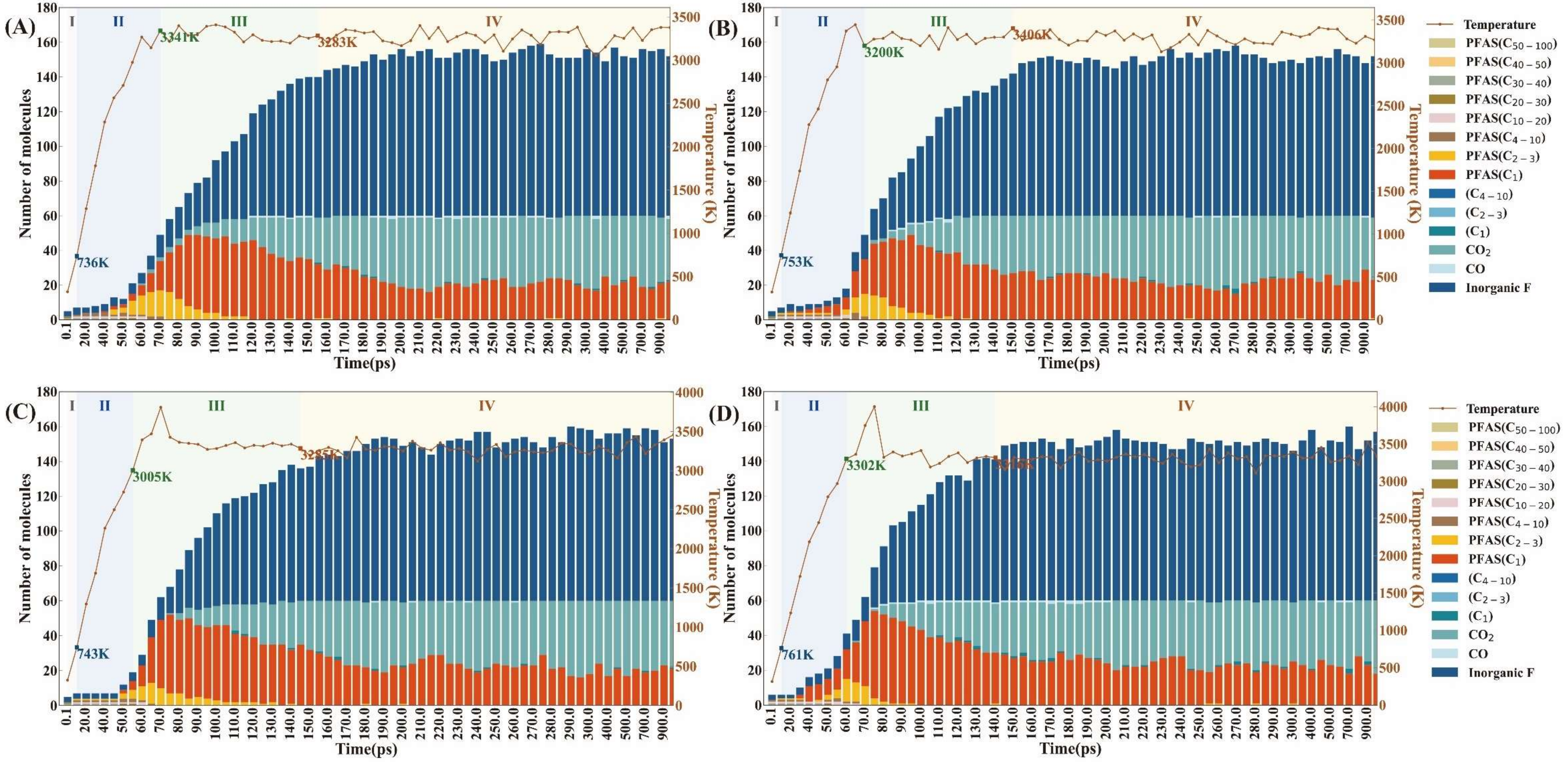

Supplementary Figure 10. MD results of the time evolution of product distribution during the thermal plasma degradation process of PTFE **under different temperature:** (a) 3400K, (B) 3600K, (C)3800K, (D) 4000K (O_2/C=2.79, H_2O/C=2.79). The MD result at **3300K** was shown in Supplementary Fig.9(B).

Supplementary Method 1 The detailed measurement and simulation methods of plasma characteristics

Before conducting the PTFE thermal plasma degradation experiment, the gas temperature of plasma jet in the presence of oxygen and steam were measured using optical emission spectroscopy (Optosky ATP2400). The emission spectra of the plasma torch under various reaction conditions were recorded using an optical fiber located 5cm away from the plasma generator. The gas temperature of plasma jet was determined by fitting the observed spectra with the standard spectra at different temperature obtained from SpectraPlot.com, an emission spectroscopy simulator website. The measuring principle aligns with the methods described in in the study by Horst et al[1]. and Fierro et al[2].

This study employs nitrogen as the plasma torch feed gas. Previous research has demonstrated that the emission density of atomic species significantly influences the optical emission in pure nitrogen discharge. Furthermore, due to the pronounced absorption of VUV (vacuum ultraviolet) radiation by molecular oxygen and water, the observation of such energetic photons poses challenges. Consequently, the emission spectra of nitrogen atoms (spanning from 450 nm to 800 nm) were chosen at different temperatures to determine the gas temperature at the core of the plasma jet.

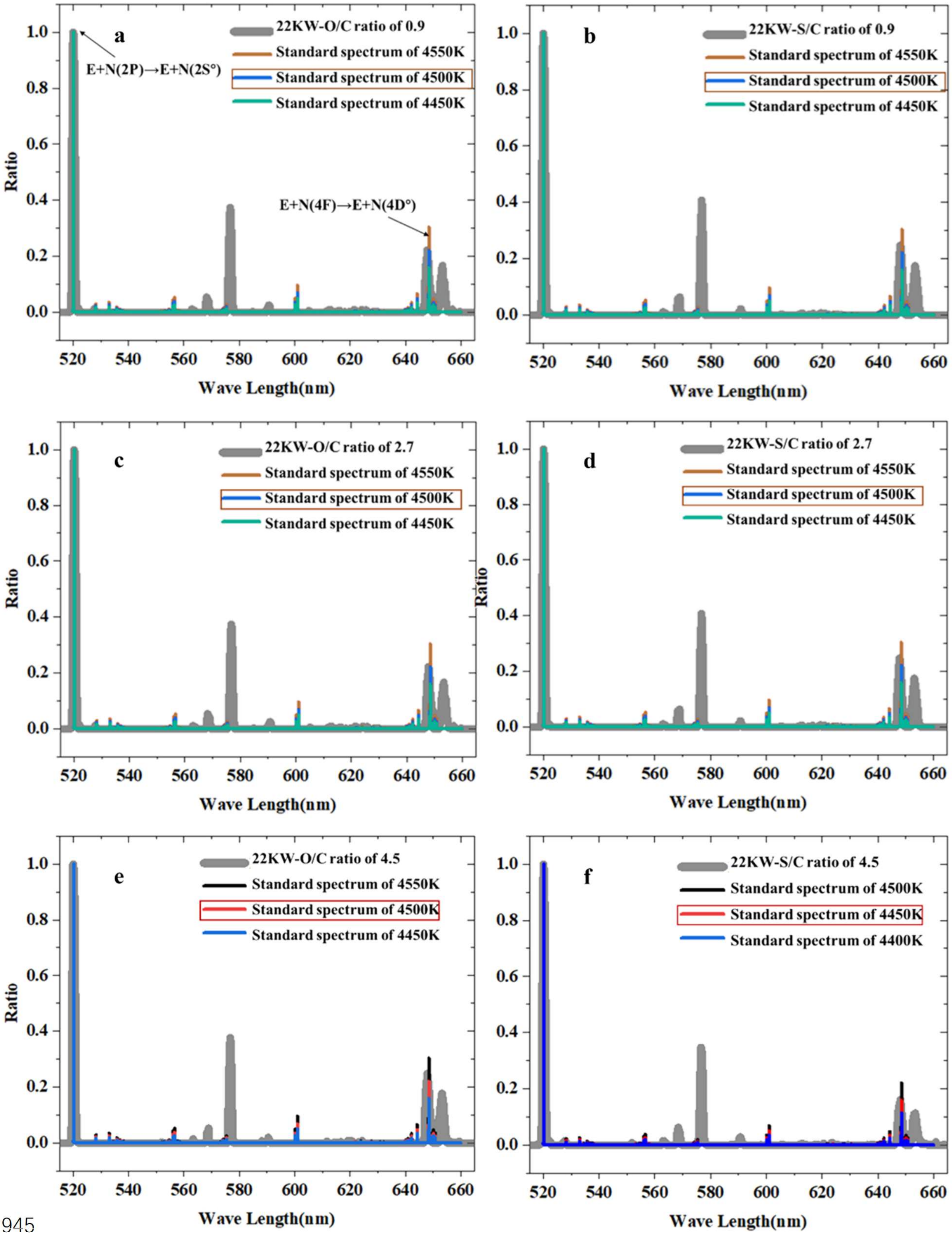
a
22KW-O/C ratio of 0.9
Standard spectrum of 4550K
Standard spectrum of 4500K
Standard spectrum of 4450K
E+N(2P)→E+N(2S°)
E+N(4F)→E+N(4D°)
b
22KW-S/C ratio of 0.9
Standard spectrum of 4550K
Standard spectrum of 4500K
Standard spectrum of 4450K
c
22KW-O/C ratio of 2.7
Standard spectrum of 4550K
Standard spectrum of 4500K
Standard spectrum of 4450K
d
22KW-S/C ratio of 2.7
Standard spectrum of 4550K
Standard spectrum of 4500K
Standard spectrum of 4450K
e
22KW-O/C ratio of 4.5
Standard spectrum of 4550K
Standard spectrum of 4500K
Standard spectrum of 4450K
f
22KW-S/C ratio of 4.5
Standard spectrum of 4500K
Standard spectrum of 4450K
Standard spectrum of 4400K
Ratio
Wave Length(nm)

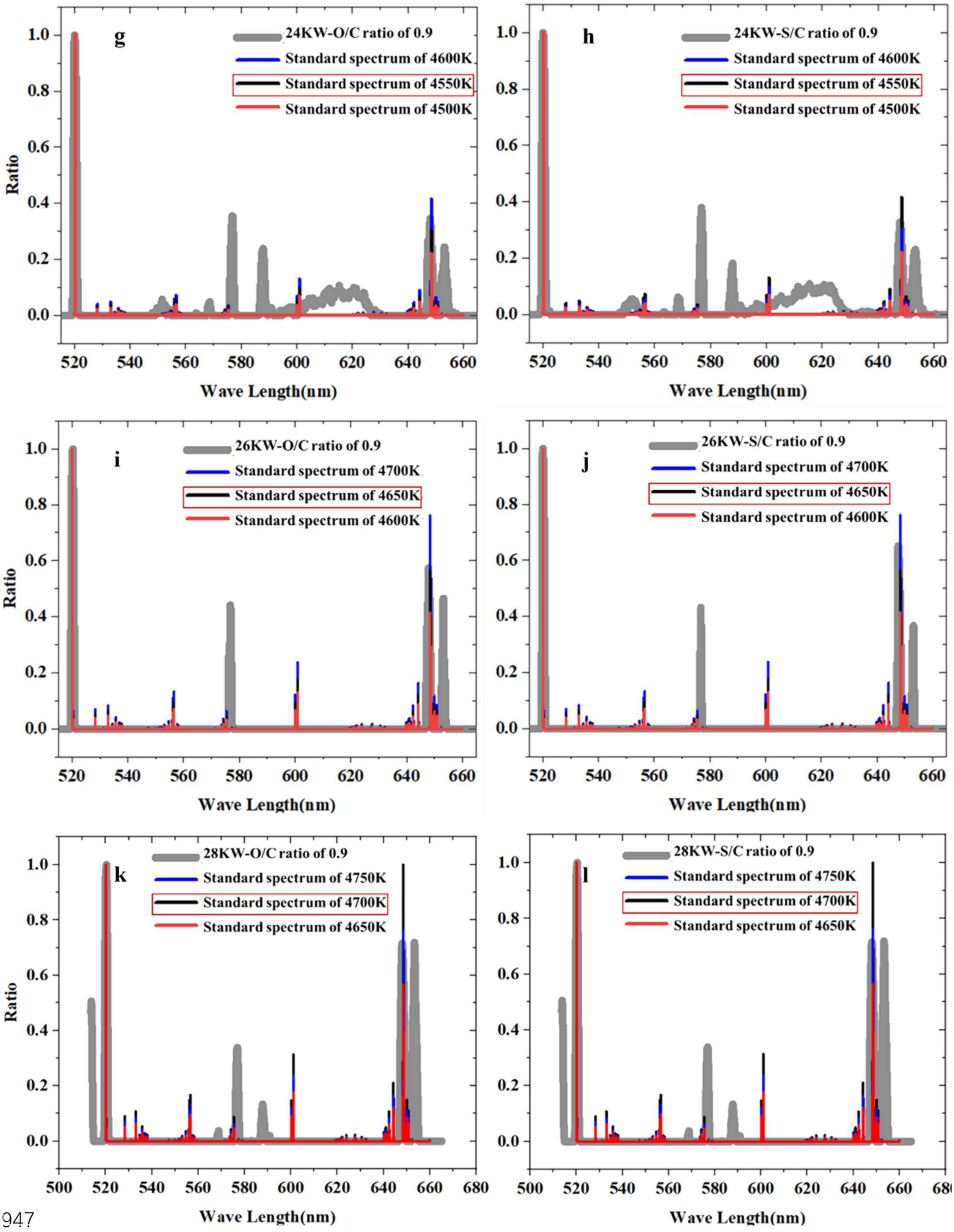
g
24KW-O/C ratio of 0.9
Standard spectrum of 4600K
Standard spectrum of 4550K
Standard spectrum of 4500K
Ratio
Wave Length(nm)
h
24KW-S/C ratio of 0.9
Standard spectrum of 4600K
Standard spectrum of 4550K
Standard spectrum of 4500K
Wave Length(nm)
i
26KW-O/C ratio of 0.9
Standard spectrum of 4700K
Standard spectrum of 4650K
Standard spectrum of 4600K
Ratio
Wave Length(nm)
j
26KW-S/C ratio of 0.9
Standard spectrum of 4700K
Standard spectrum of 4650K
Standard spectrum of 4600K
Wave Length(nm)
k
28KW-O/C ratio of 0.9
Standard spectrum of 4750K
Standard spectrum of 4700K
Standard spectrum of 4650K
Ratio
Wave Length(nm)
l
28KW-S/C ratio of 0.9
Standard spectrum of 4750K
Standard spectrum of 4700K
Standard spectrum of 4650K
Wave Length(nm)

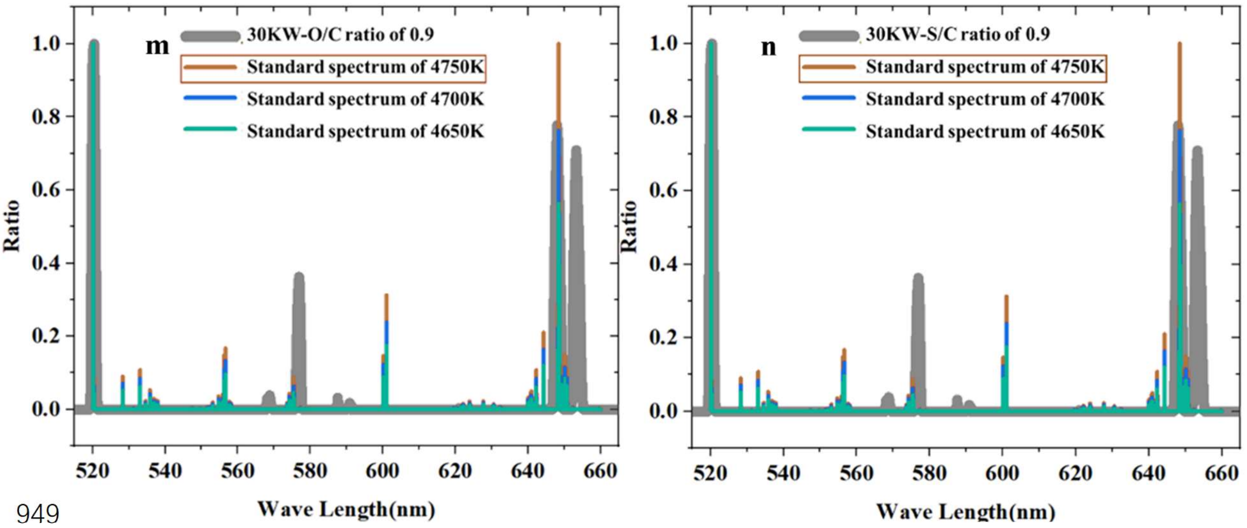

Supplementary Figure 11. The comparison between optical emission spectra of plasma jet and the standard spectrum under different input power and atmosphere (in the wavelengths range of 640nm-760nm): (a) power = 22 kW, O_2/C = 0.9, (b) power = 22 kW, H_2O/C = 0.9, (c) power = 22 kW, O_2/C = 2.7, (d) power = 22 kW, H_2O/C = 2.7, (e) power = 22 kW, O_2/C = 4.5, (f) power = 22 kW, H_2O/C = 4.5, (g) power = 24 kW, O_2/C = 0.9, (h) power = 24 kW, H_2O/C = 0.9, (i) power = 26 kW, O_2/C = 0.9, (j) power = 26 kW, H_2O/C = 0.9, (k) power = 28 kW, O_2/C = 0.9, (I) power = 28 kW, H_2O/C = 0.9, (m) power = 30 kW, O_2/C = 0.9, (n) power = 30 kW, H_2O/C = 0.9.

As shown in Fig S10, the gas temperature is mainly determined by the input power and exhibits low sensitivity to variations in the gas agent concentrations (O_2 or H_2O). This observation can be attributed to the limited involvement of oxygen and steam in the plasma generation process. Based on the comparison results of optical emission spectra of plasma jet, 4500K, 4550K, 4650K, 4700K and 4750K were selected to represent the simulated gas temperatures at the core of the plasma jet at the input power of 22 kW, 24 kW, 26 kW, 28 kW and 30 kW, respectively.

Subsequently, a two-dimensional numerical model was developed using based on Comsol software in order to investigate the temperature distribution of the plasma jet. This model takes into account the DC plasma generator's structure (as shown in Fig S11), the core temperature, and the voltage and current values of the plasma generator. The fundamental assumptions of this model include: (1) The plasma model is a two-dimensional axisymmetric representation; (2) The plasma is treated as a continuous Newtonian fluid with steady, compressible laminar flow; (3) The plasma adheres to the assumption of local thermodynamic equilibrium and local chemical equilibrium; (4) The plasma is quasi-neutral and optically thin, with only volumetric radiation losses considered for radiation; (5) Gravity effects are neglected.

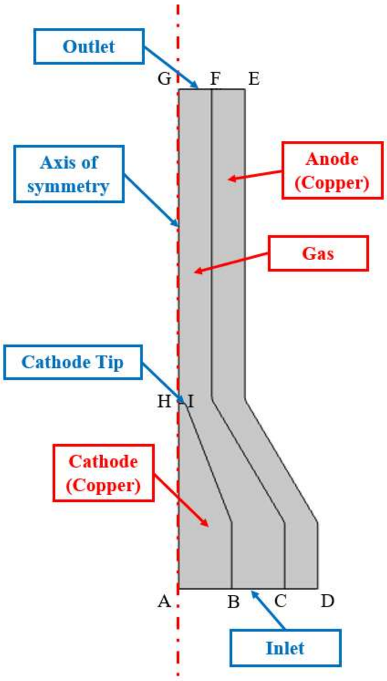

Supplementary Figure 12. The structure of DC plasma generator used in this study

The control equation used in this model includes mass conservation equation (Supplementary Equation (1)), momentum conservation equation (Supplementary Equation (2)), energy conservation equation (Supplementary Equation (3)), ideal gas state equation (Supplementary Equation (4)), and maxwell equation (Supplementary Equation (5) – (8)).

$$\nabla \cdot (\rho \boldsymbol{u}) = 0 \tag{1}$$

Where, ρ represents the gas density, $\boldsymbol{u}$ represents velocity field.

$$\rho \boldsymbol{u} \cdot \nabla \boldsymbol{u} = \nabla \cdot \left\{ -p\hat{I} + \mu[(\nabla \boldsymbol{u}) + (\nabla \boldsymbol{u})^T] - \frac{2}{3}\mu(\nabla \cdot \boldsymbol{u}) \right\} + \boldsymbol{j} \times \boldsymbol{B} \tag{2}$$

Where, p represents the gas pressure, μ represents the dynamic viscosity，$\boldsymbol{j} \times \boldsymbol{B}$ represents the Lorentz force, $\boldsymbol{j}$ represents the electric current density，$\boldsymbol{B}$ represents the magnetic strength.

$$\nabla \cdot (\rho C_p \nabla T_g \cdot \boldsymbol{u}) - \nabla(k \cdot \nabla T_g) = Q_p + Q_{Joule} + Q_h + Q_{rad} + \tau : \nabla \boldsymbol{u} \tag{3}$$

Where, C_p represents the constant pressure specific heat capacity, T_g represents the gas temperature, k represents the heat conductivity coefficient，Q_p represents the work done by pressure, $Q_{Joule} = \boldsymbol{j} \cdot \boldsymbol{E}$ represents the Joule heating term, Q_h represents the enthalpy transportation，Q_{rad} represents the Radiated power per unit volume，which is calculated by $Q_{rad} = 4\pi\varepsilon_r$，$\varepsilon_r$ represents the radiation coefficient per unit volume. $\tau : \nabla \boldsymbol{u}$ represents the viscous dissipative term.

$$p = \rho R_s T_g \tag{4}$$

Where, R_s represents the gas constant.

$$\boldsymbol{j} = \sigma(\boldsymbol{E} + \boldsymbol{u} \times \boldsymbol{B}) \qquad (5)$$

$$\nabla \cdot \boldsymbol{j} = 0 \qquad (6)$$

$$\boldsymbol{E} = -\nabla \Phi \qquad (7)$$

$$\nabla \times \mathbf{B} = \mu_0 \boldsymbol{j} \qquad (8)$$

Where, **E** represents the electric field, Φ represents the electric potential， σ represents the conductivity, μ_0 represents the relative magnetic permeability.

Furthermore, the boundary conditions of this Comsol-based model include:

	Entrance	Exit	Wall surfaces
Fluid field	$v_z = Q/S$<br>$v_r = 0$	$p = 1atm$	$v = 0$

* Flow Q is given by actual operating conditions.

	Entrance	Anode outer wall	Cathode point	Outer boundary (AB, CD, EFG)
Heat Transfer	$T = 300K$	$1000K$	$T = 3500K$	$-\boldsymbol{n} \cdot \boldsymbol{q} = 0$

The external surface of the anode serves as an artificially imposed boundary with a constant temperature condition, whereas the cathode is held at a consistent temperature of 1000K, except at the tip position, where the condition $-\boldsymbol{n} \cdot \boldsymbol{q} = 0$ represents the adiabatic boundary.

	Cathode tip	Anode outer wall	Outer boundary（IB, BCD, EFG)
Electric Field	$V = constant$	$\Phi = 0V$	$\boldsymbol{n} \cdot \boldsymbol{j} = 0$

* The voltage boundary is determined by the practical operating conditions, where $n \cdot j=0$ represents the insulating boundary, and the solid cathode is excluded from the electromagnetic field calculations.

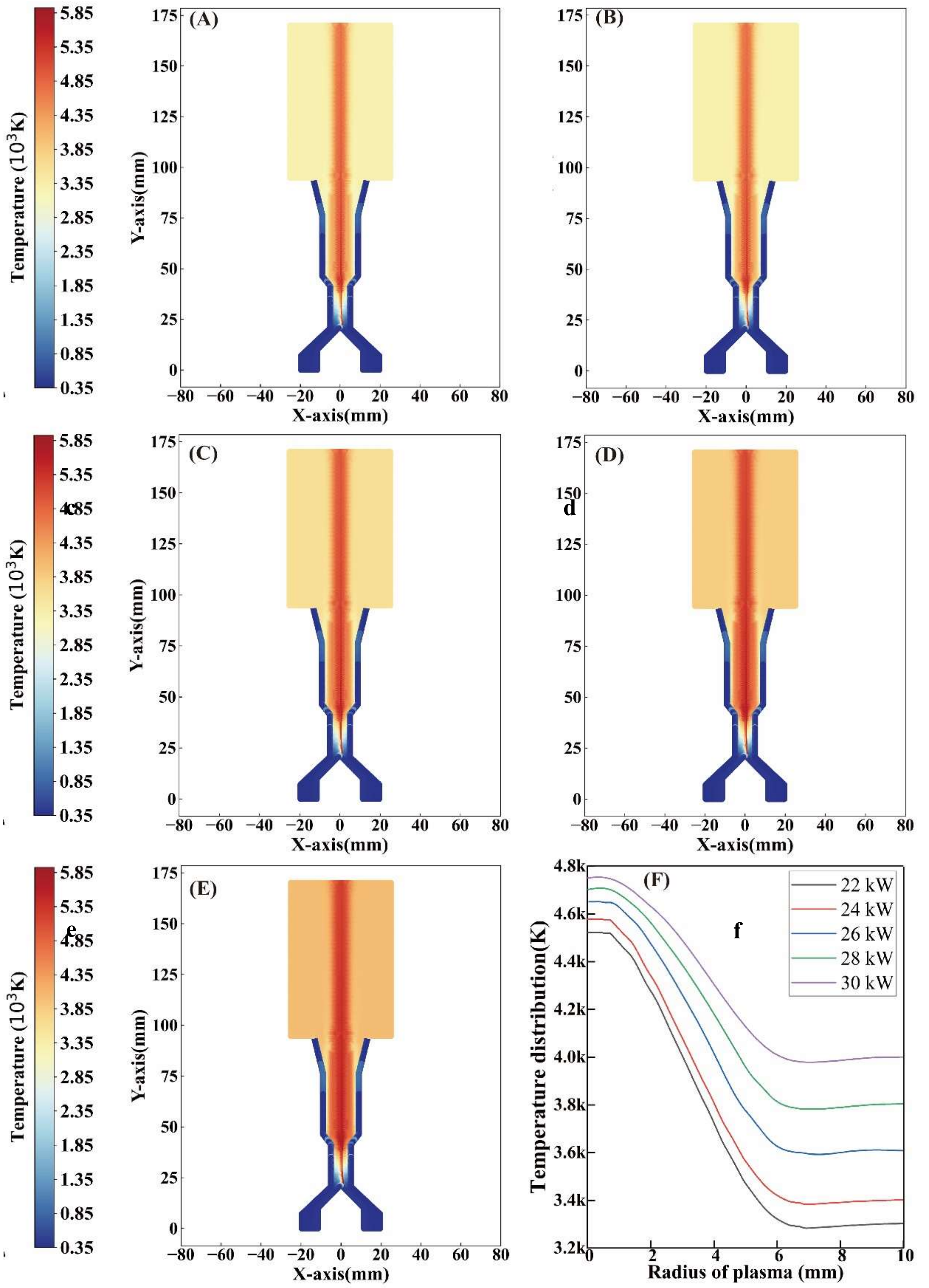

Supplementary Figure 13. The 2D temperature distribution of plasma jet at (A) 22 kW (B) 24 kW (C) 26 kW (D) 28 kW (E) 30 kW and (F) the horizontal temperature distribution of plasma jet at the area 10 cm away from the plasma generator.

The observations presented in Supplementary Fig.12 validate the consistency of the simulated core temperatures by the two-dimensional numerical model with the measurements depicted in Supplementary Fig.10. Furthermore, the gas temperature experiences a rapid decline as the distance from the center increases from 2mm to 6mm, followed by a relatively stable trend within the range of 6mm to 10mm. Given the 10mm diameter of the plasma jet and the predominant occurrence of reaction regions near the plasma jet's periphery, the temperatures of 3300K, 3400K, 3600K, 3800K, and 4000K were selected as the reaction temperatures for MD simulations at the input power of 22 kW, 24 kW, 26 kW, 28 kW and 30 kW, respectively.

The density of activated particles was simulated using the measured temperature and a 0-D numerical simulation of plasma kinetics. The equation for this kinetic model is as follows:

$$\frac{\mathrm{d}[N_i]}{\mathrm{d}t} = \sum_{j=1}^{j_{max}\Sigma\ \ \mathrm{i=1}_{speciesmax}} \mathrm{Q_{ij}} \qquad (9)$$

Where $\mathrm{Q_{ij}}(t)$ represents the source term of the j reaction to the *i* particle, N_i represents the density of the *i* particle. Assuming that the arc is quasi-equilibrium, this model sets the electron temperature and gas temperature based on measurements obtained from the spectrum.

The detailed simulated methods referred to the studies by Nicolas et al. (Chapter 5&6) [3], by Wang et al[4] and by Capitelli et al.[5]. The reactions considered in the calculations (Supplementary Equation (9)) mainly includes:

Supplementary Table 4 The main kinetic mechanism in this work is taken from Laux et al[6].

Reaction		Temperature independent variable k_f	k_b	$k_f = AT^b \exp(-\frac{E}{RT})$ A(cm^3 s^{-1})	b	E/R(K)	Reference	
Dissociation/recombination of O_2,NO,N_2 (Laux et al.1999)								
(R1)	$O_2 + N_2 \leftrightarrow 2O + N_2$	T_g	T_g	3.32 10^{-3}	-1.5	59,500	(Park,1993)	*
(R2)	$O_2 + O_2 \leftrightarrow 2O + O_2$	T_g	T_g	3.32 10^{-3}	-1.5	59,500	(Park,1993)	*
(R3)	$O_2 + NO \leftrightarrow 2O + NO$	T_g	T_g	3.32 10^{-3}	-1.5	59,500	(Park,1993)	*
(R4)	$O_2 + N \leftrightarrow 2O + N$	T_g	T_g	1.66 10^{-2}	-1.5	59,500	(Park,1993)	*
(R5)	$O_2 + O \leftrightarrow 2O + O$	T_g	T_g	1.66 10^{-2}	-1.5	59,500	(Park,1993)	*
(R8)	$NO + N_2 \leftrightarrow N + O + N_2$	T_g	T_g	8.30 10^{-9}	0.0	75,500	(Park,1993)	*
(R9)	$NO + O_2 \leftrightarrow N + O + O_2$	T_g	T_g	8.30 10^{-9}	0.0	75,500	(Park,1993)	*
(R10)	$NO + NO \leftrightarrow N + O + NO$	T_g	T_g	1.83 10^{-7}	0.0	75,500	(Park,1993)	*
(R11)	$NO + N \leftrightarrow N + O + N$	T_g	T_g	1.83 10^{-7}	0.0	75,500	(Park,1993)	*
(R12)	$NO + O \leftrightarrow N + O + O$	T_g	T_g	1.83 10^{-7}	0.0	75,500	(Park,1993)	*
(R13)	$N_2 + N_2 \leftrightarrow 2N + N_2$	T_g	T_g	1.16 10^{-2}	-1.6	113,200	(Park,1993)	*
(R14)	$N_2 + O_2 \leftrightarrow 2N + O_2$	T_g	T_g	1.16 10^{-2}	-1.6	113,200	(Park,1993)	*
(R15)	$N_2 + NO \leftrightarrow 2N + NO$	T_g	T_g	1.16 10^{-2}	-1.6	113,200	(Park,1993)	*
(R16)	$N_2 + N \leftrightarrow 2N + N$	T_g	T_g	4.98 10^{-2}	-1.6	113,200	(Park,1993)	*
(R17)	$N_2 + O \leftrightarrow 2N + O$	T_g	T_g	4.98 10^{-2}	-1.6	113,200	(Park,1993)	*
Zeldovich reactions (Laux et al. 1999)								
(R18)	$N_2 + O \leftrightarrow NO + N$	T_g	T_g	1.06 10^{-6}	-1.0	38,400	(Park,1993)	*
(R19)	$NO + O \leftrightarrow O_2 + N$	T_g	T_g	1.39 10^{-11}	0.0	19,400	(Park,1993)	*
Adsorption, ionization/desorption (Laux et al. 1999)								
R(20)	$N + O \rightarrow NO^+ + e$	T_g	T_e	1.46 10^{-15}	1.0	31,900	(Park,1993)	
R(21)	$N + N \rightarrow N_2^+ + e$	T_g	T_e	9.97 10^{-17}	1.5	67,500	(Park,1993)	
Charge transfer (Laux et al. 1999)								
(R28)	$N^+ + N_2 \leftrightarrow N + N_2^+$	T_g	T_g	1.66 10^{-12}	0.5	12,000	(Park,1993)	
(R29)	$NO^+ + O \leftrightarrow N^+ + O_2$	T_g	T_g	1.66 10^{-12}	0.5	77,200	(Park,1993)	*
(R30)	$NO + O^+ \leftrightarrow N^+ + O_2$	T_g	T_g	2.32 10^{-19}	1.9	26,600	(Park,1993)	
(R31)	$NO^+ + N \leftrightarrow N_2 + O^+$	T_g	T_g	5.65 10^{-11}	-1.08	12,800	(Park,1993)	
(R32)	$O^+ + N_2 \leftrightarrow N_2^+ + O$	T_g	T_g	1.49 10^{-12}	0.36	22,800	(Park,1993)	
(R33)	$NO^+ + N \leftrightarrow N_2^+ + O$	T_g	T_g	1.20 10^{-10}	0.0	35,500	(Park,1993)	*
(R34)	$O_2^+ + N \leftrightarrow N^+ + O_2$	T_g	T_g	1.44 10^{-10}	0.14	28,600	(Park,1993)	
(R35)	$O_2^+ + N_2 \leftrightarrow N_2^+ + O_2$	T_g	T_g	1.64 10^{-11}	0.0	40,700	(Park,1993)	
(R36)	$NO^+ + O_2 \leftrightarrow NO + O_2^+$	T_g	T_g	3.99 10^{-11}	0.41	32,600	(Park,1993)	
(R37)	$NO^+ + O \leftrightarrow N + O_2^+$	T_g	T_g	1.20 10^{-11}	0.29	48,600	(Park,1993)	

Supplementary Table 5 Supplementary reactions of molecules calculated with BOLSIG+

	Electron excitation of N_2	Collision cross section
(R37)	$N_2 + e \leftrightarrow N_2(Y) + e$	(Pitchford and Phelps, 1982)
	Y = A, B, C, a’	
(R38)	$N_2(Z) + e \leftrightarrow N_2(Y) + e$	(Bacri and Medani,1982)
	Z = A, B, C, a’	
	Y = B, C, a’	
	**Ionization and dissociation of N_2**	
(R39)	$N_2(X) + e \leftrightarrow N_2^+(Y) + 2e$	(Isola, Gomez and Guerra,2010)
	Y = X, B	
(R40)	$N_2(X) + e \leftrightarrow N_2^+(Y) + 2e$	(Bacri and Medani, 1982)
	Y = A, C	
(R41)	$N_2(Z) + e \leftrightarrow N_2^+(Y) + 2e$	(Bacri and Medani, 1982)
	Z = A, B, C, a’	
	Y = X, A, B, C	
(R42)	$N_2(Z) + e \leftrightarrow N + N + e$	(Bacri and Medani, 1982)
	Z = X, A	
(R43)	$N_2(Z) + e \leftrightarrow N + N(^2D) + e$	(Bacri and Medani, 1982)
	Y = B, a’, C	
	**Vibrational excitation of N_2+**	
(R44)	$N_2^+(Z) + e \leftrightarrow N_2^+(Y) + e$	(Bacri and Medani, 1982)
	Z = X, A, B, C	
	Y = X, A, B, C	

Supplementary Table 6 Transitions between atomic excited states and their ionization. The backward reaction rates are calculated by multiplying forward reaction rates and partition function[7, 8, 9, 10, 11, 12, 13]

Reaction		$k_f = AT^b \exp\left(-\frac{E}{RT}\right)$ A(cm^3 s^{-1})	b	E/R(K)	Ref.
**Transition of excited states of atoms**					
(R45)	$N(^4S^0) + e \leftrightarrow N(Y) + e$<br>$Y = {}^2D^0, {}^2P^0, {}^4P, {}^2P, {}^4P, {}^2S^0, {}^4D^0$	BOLSIG+			(Wang et al., 2014)
(R52)	$N(^2D^0) + e \leftrightarrow N(Y) + e$<br>$Y = {}^2P^0, {}^4P, {}^2P, {}^4P, {}^2S^0, {}^4D^0$	BOLSIG+			(Wang et al., 2014)
(R58)	$N(^2P^0) + e \leftrightarrow N(Y) + e$<br>$Y = {}^4P, {}^2P, {}^4P, {}^2S^0, {}^4D^0$	BOLSIG+			(Wang et al., 2014)
(R63)	$N(^4P, 3s) + e \leftrightarrow N(Y) + e$<br>$Y = {}^2P, {}^4P, {}^2S^0, {}^4D^0$	BOLSIG+			(Wang et al., 2014)
(R67)	$N(^2P, 3s) + e \leftrightarrow N(Y) + e$<br>$Y = {}^4P, {}^2S^0, {}^4D^0$	BOLSIG+			(Wang et al., 2014)
(R70)	$N(^4P, 2s^22p^4) + e \leftrightarrow N(Y) + e$<br>$Y = {}^2S^0, {}^4D^0$	BOLSIG+			(Wang et al., 2014)
(R72)	$N(^2S^0, 3p) + e \leftrightarrow N(Y) + e$	BOLSIG+			(Wang et al., 2014)
(R73)	$O(^3P) + e \leftrightarrow O(^1D, {}^1S) + e$	BOLSIG+			(Tayal et al., 2016)
(R75)	$O(^3D) + e \leftrightarrow O(^1S) + e$	BOLSIG+			(Tayal et al., 2016)
**Ionization of atom excited state**					
(R76)	$N(^1S^0) + e \leftrightarrow N^+ + 2e$	BOLSIG+			(Wang et al., 2014)
(R77)	$N(^2D^0) + e \leftrightarrow N^+ + 2e$	BOLSIG+			(Wang et al., 2014)
(R78)	$N(^2P^0) + e \leftrightarrow N^+ + 2e$	BOLSIG+			(Wang et al., 2014)
(R79)	$N(^4P, 3s) + e \leftrightarrow N^+ + 2e$	$9.33\ 10^{-10}$	0.49	49,120	(Ciccarino et al., 2019)
(R80)	$N(^2P, 3s) + e \leftrightarrow N^+ + 2e$	$1.67\ 10^{-9}$	0.45	45,090	(Ciccarino et al., 2019)
(R81)	$N(^4P, 2s^22p^4) + e \leftrightarrow N^+ + 2e$	$6.57\ 10^{-11}$	0.74	41,660	(Ciccarino et al., 2019)
(R82)	$N(^2S^0, 3p) + e \leftrightarrow N^+ + 2e$	$3.74\ 10^{-9}$	0.42	34,580	(Ciccarino et al., 2019)
(R83)	$N(^4D^0, 3p) + e \leftrightarrow N^+ + 2e$	$4.82\ 10^{-9}$	0.41	32,820	(Ciccarino et al., 2019)

Supplementary Table 7 Supplementary reactions for the thermal spark transition.

Reaction	Freedom of temperature		$k_f = AT^b \exp\left(-\frac{E}{RT}\right)$			Ref.
			A(cm^3 s^{-1})	b	E/R(K)	
Extinction of the excited state of N_2 electron						
(R87)	N2(A) + O2 ↔ N2(X) + 2O	T_g	9.81 10^{-14}	0.5	0	(Kossyi et al., 1992)
(R88)	N2(A) + O2 ↔ N2(X) + O_2	T_g	4.33 10^{-14}	0.5	0	(Kossyi et al., 1992)
(R89)	N2(A) + O ↔ NO+ N(^{2}D)	T_g	4.00 10^{-11}	0.0	0	(Thomas et al., 1996)
(R90)	N2(B) + N2 ↔ N2(A) + N_2	T_g	1.00 10^{-11}	0.0	0	(Kossyi et al., 1992)
(R91)	N2(B) + O2 ↔ N2(X) + 2O	T_g	3.00 10^{-10}	0.0	0	(Kossyi et al., 1992)
(R92)	N2(B) + O2 ↔ NO+ N(^{2}D)	T_g	3.00 10^{-10}	0.0	0	(Shkurenkov et al., 2014)
(R93)	N2(C) + N2 ↔ N2(B) + N_2	T_g	1.00 10^{-11}	0.0	0	(Kossyi et al., 1992)
(R94)	N2(C) + O2 ↔ N2(X) + O +O*	T_g	1.73 10^{-11}	0.3	0	(Rusterholtz et al., 2013)
(R95)	N2(C) + O ↔ NO + N(^{2}D)	T_g	3.00 10^{-10}	0.0	0	(Shkurenkov et al., 2014)
(R96)	N2(a') + N_2 ↔ N_2(B) + N_2	T_g	2.00 10^{-13}	0.0	0	(Piper, 1987)
(R97)	N2(a') + O_2 ↔ N_2(X) + O + O*	T_g	2.80 10^{-11}	0.0	0	(Piper, 1987)
(R98)	N2(a') + O ↔ NO + N(^{2}D)	T_g	3.00 10^{-10}	0.0	0	(Shkurenkov et al., 2014)

Supplementary Table 8 Supplementary reactions for the Dissociation/recombination of O_2, H_2O, N_2[13, 14, 15, 16, 17, 18, 19, 20].

Reaction		k (two body reaction: $cm^3\ s^{-1}$ three body reaction: $cm^6\ s^{-2}$)	Ref.		
Dissociation/recombination of O_2, H_2O, N_2					
(R99)	O·+·OH ↔ O2 + H·	[11]	(R107)	O3+·OH ↔ HO2· + O2	[14]
(R100)	O(^{1}D) + H2O ↔ 2·OH	[12]	(R108)	OH($A^3Σ_u^-$) +H2O ↔ ·OH + H2O	[15]
(R101)	OH·+·OH↔ H2O +·O	[11]	(R109)	·O + HO2· ↔ ·OH +O2	[16]
(R102)	·OH+·H ↔ H_2 + ·O	[11]	(R110)	N^+ + ·OH ↔ NO^+ +·H	[17]
(R103)	$N_2(A^3Σ_u^-)$ + H2O ↔ ·OH+·H + N2	[13]	(R111)	N^+ + ·OH ↔ OH^++·N	[17]
(R104)	N·+·OH ↔ NO + ·H	[11]	(R112)	·N +·OH ↔ NO +·H	[17]
(R105)	O3+·H ↔ ·OH + O2	[14]	(R113)	OH($A^3Σ_u^-$) +N2 ↔N2 +·OH	[18]
(R106)	·H +O2 + N_2(X, A, B, C, a') ↔ HO2·+ N_2(X, A, B, C, a')	[13]	(R114)	OH($A^3Σ_u^-$) +O2 ↔ O2 +·OH	[18]

Supplementary Fig.14 displayed the results of activate particles density of plasma under different O_2/C ratio. The ·OH, ·H and ·O free radicals mainly resulted from the collisions involving the metastable state of $N_2(A^3Σ_u^-)$ and H_2O, O_2 molecules. N_2 and NO contributes little to the polymer decomposition process. Consequently, the MD simulations primarily investigate the impacts of ·OH, ·H, and ·O free radicals on PTFE decomposition. The densities of these free radicals are presented in Supplementary Table 9. The conversion formula from the free radical density to the number of free radicals in the periodic unit of MD simulation is as follows:

$$n_i = \frac{c_i \times V_r \times t \times 3600}{NA \times m_F / M_F} \times n_f \qquad (10)$$

where, *i* represents ·OH, ·H and ·O, c_i represents the density of free radicals (cm^{-3}

s^{-1}), *Vr* represents the volume of reaction region and herein, the volume of crucible containing raw materials is used (10173.6 cm^3), *t* represents the reaction time (10 min), NA represents the Avogadro constant (6.02×10^{23}), m_F represents the mass amount of fluorine in raw materials (152 g), M_F represents the molar mass of fluorine, n_f represents the number of fluorine atom in the periodic unit (122).

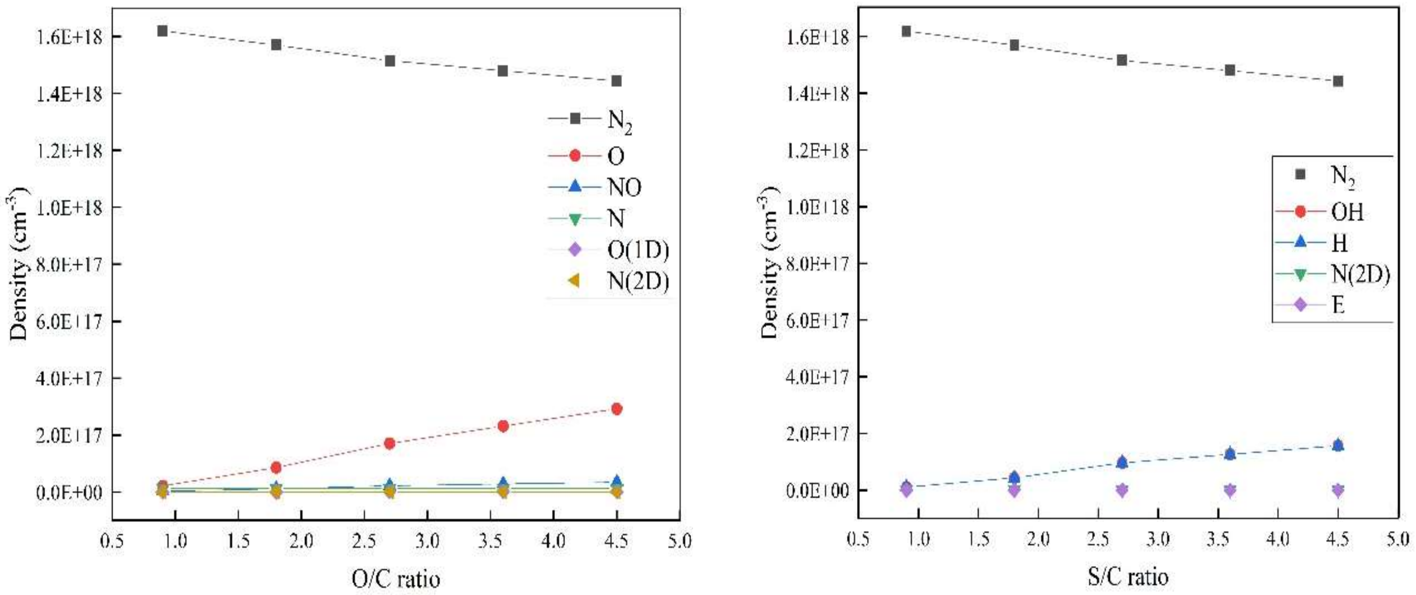

Supplementary Figure 14. The density of activate particles at different (a) O_2/C ratio (b)H_2O/C ratio (22kW)

Supplementary Table 9. The density of activate particles in the mixed atmosphere (22kW)

O_2/C ratio	H_2O/C ratio	Density of O	Density of OH	Density of H
2.79	2.79	1.712E+17	1.28E+17	1.28E+17
0.93	4.65	2.14E+16	3.85E+17	3.85E+17
2.79	2.79	1.712E+17	1.28E+17	1.28E+17
2.79	0.93	1.712E+17	1.07E+16	1.07E+16
0.93	0.93	2.14E+16	1.07E+16	1.07E+16
4.65	4.65	2.996E+17	3.85E+17	3.85E+17
4.65	2.79	2.996E+17	1.28E+17	1.28E+17
2.79	2.79	1.712E+17	1.28E+17	1.28E+17
2.79	2.79	1.712E+17	1.28E+17	1.28E+17
4.65	0.93	2.996E+17	1.07E+16	1.07E+16
2.79	4.65	1.712E+17	3.85E+17	3.85E+17
2.79	2.79	1.712E+17	1.28E+17	1.28E+17
2.79	2.79	1.712E+17	1.28E+17	1.28E+17
0.93	2.79	2.14E+16	1.28E+17	1.28E+17

Supplementary Table 10. Conditions of MD reaction system of PTFE plasma degradation

	System number	O_2/C ratio	H_2O/C ratio	PTFE molecule number	Oxygen molecule number	H_2O molecule number	·O molecule number	·OH molecule number	·H molecule number	Input power (kW) /Temperature (K)	Density (g cm^{-3})
The effect of O_2/C ratio	0	0	0	1	0	0	0	0	0	22/3300	1.00
	0.93-C*	0.93	0	1	58	0	0	0	0	22/3300	1.00
	0.93	0.93	0	1	57	0	2	0	0	22/3300	1.00
	1.86-C*	1.86	0	1	117	0	0	0	0	22/3300	1.00
	1.86	1.86	0	1	113	0	8	0	0	22/3300	1.00
	2.79-C*	2.79	0	1	178	0	0	0	0	22/3300	1.00
	2.79	2.79	0	1	170	0	16	0	0	22/3300	1.00
	3.72-C*	3.72	0	1	238	0	0	0	0	22/3300	1.00
	3.72	3.72	0	1	227	0	22	0	0	22/3300	1.00
	4.65-C*	4.65	0	1	298	0	0	0	0	22/3300	1.00
	4.65	4.65	0	1	284	0	28	0	0	22/3300	1.00
The effect of H_2O/C ratio	0.93-C*	0	0.93	1	0	58	0	0	0	22/3300	1.00
	0.93	0	0.93	1	0	57	0	1	1	22/3300	1.00
	1.86-C*	0	1.86	1	0	117	0	0	0	22/3300	1.00
	1.86	0	1.86	1	0	113	0	4	4	22/3300	1.00
	2.79-C*	0	2.79	1	0	179	0	0	0	22/3300	1.00
	2.79	0	2.79	1	0	170	0	9	9	22/3300	1.00
	3.72-C*	0	3.72	1	0	239	0	0	0	22/3300	1.00
	3.72	0	3.72	1	0	227	0	12	12	22/3300	1.00
	4.65-C*	0	4.65	1	0	299	0	0	0	22/3300	1.00
	4.65	0	4.65	1	0	284	0	15	15	22/3300	1.00
The effect of temperature	T1	2.79	2.79	1	170	170	16	12	12	22/3300	1.00
	T2	2.79	2.79	1	170	170	16	12	12	24/3400	1.00
	T3	2.79	2.79	1	170	170	16	12	12	26/3600	1.00
	T4	2.79	2.79	1	170	170	16	12	12	28/3800	1.00
	T5	2.79	2.79	1	170	170	16	12	12	30/4000	1.00

Supplementary Table 10. Continued

	System number	O_2/C ratio	H_2O/C ratio	PTFE molecule number	Oxygen molecule number	H_2O molecule number	·O molecule number	·OH molecule number	·H molecule number	Input power (kW) /Temperature (K)	Density (g cm^{-3})
The synergic effect of O_2/C, H_2O/C ratio and temperature	M1	0.9	2.7	1	57	170	2	12	12	22/3300	1.00
	M2	2.7	0.9	1	170	57	16	1	1	30/4000	1.00
	M3	4.5	0.9	1	284	57	28	1	1	26/3600	1.00
	M4	2.7	2.7	1	170	170	16	12	12	26/3600	1.00
	M5	4.5	4.5	1	284	284	28	36	36	26/3600	1.00
	M6	2.7	4.5	1	170	284	16	36	36	30/4000	1.00
	M7	2.7	4.5	1	170	284	16	36	36	22/3300	1.00
	M8	4.5	2.7	1	284	170	28	12	12	22/3300	1.00
	M9	2.7	2.7	1	170	170	16	12	12	26/3600	1.00
	M10	2.7	2.7	1	170	170	16	12	12	26/3600	1.00
	M11	0.9	2.7	1	57	170	2	12	12	30/4000	1.00
	M12	0.9	4.5	1	57	284	2	36	36	26/3600	1.00
	M13	0.9	0.9	1	57	57	2	1	1	26/3600	1.00
	M14	2.7	2.7	1	170	170	16	12	12	26/3600	1.00
	M15	4.5	2.7	1	284	170	28	12	12	30/4000	1.00
	M16	2.7	0.9	1	170	57	16	1	1	22/3300	1.00
	M17	2.7	2.7	1	170	170	16	12	12	26/3600	1.00

* These reaction systems represent the conventional high temperature thermochemical conversion technology without free radicals.

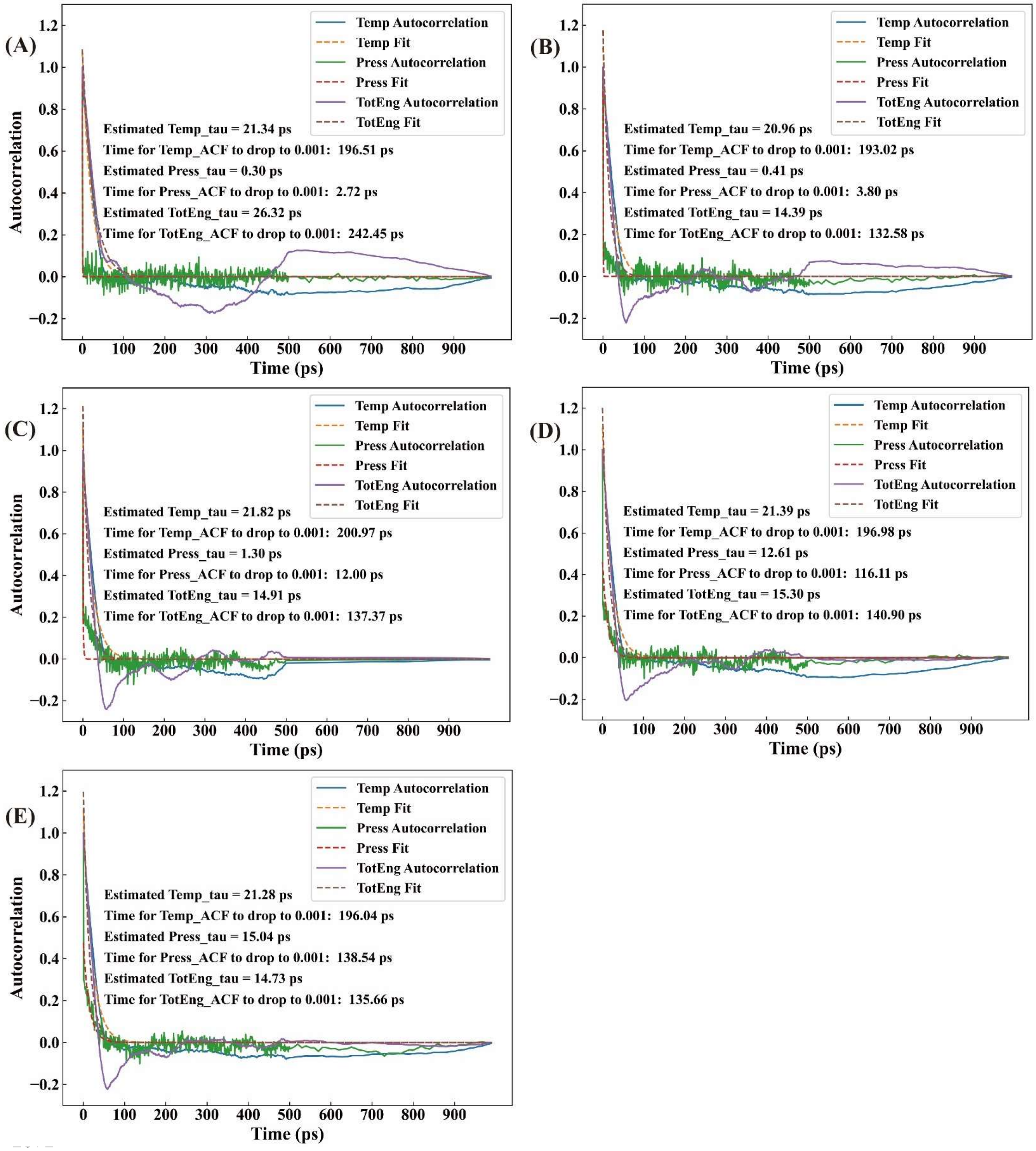

Supplementary Figure 15. Normalized time autocorrelation functions for the PTFE plasma gasification processes **at the O_2/C ratio of** (A) 0.93, (B)1.86, (C) 2.79, (D) 3.72, (E) 4.65 (temperature=3300K)

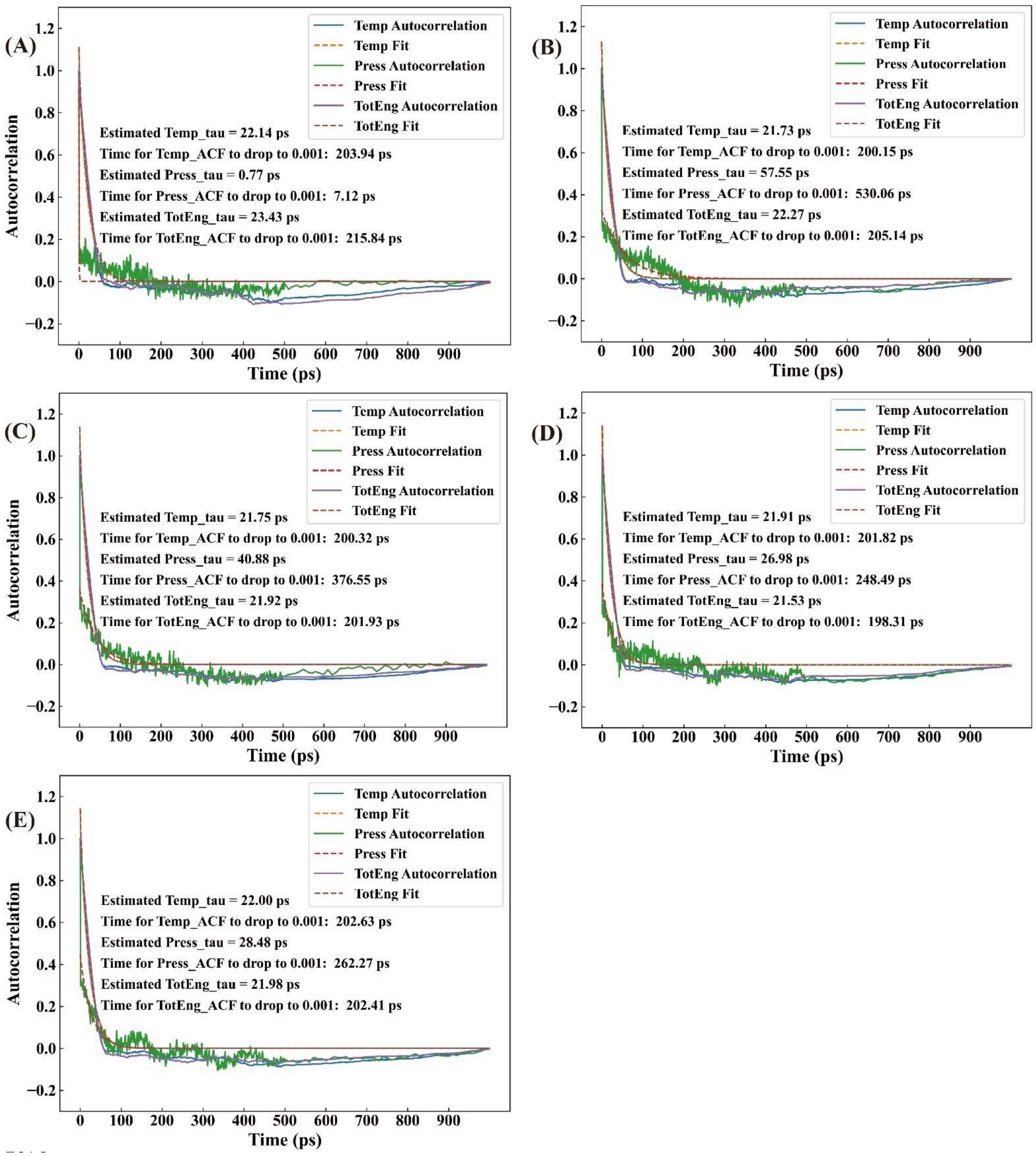

Supplementary Figure 16. Normalized time autocorrelation functions for the PTFE plasma gasification processes **at the H_2O/C ratio of** (A) 0.93, (B)1.86, (C) 2.79, (D) 3.72, (E) 4.65 (temperature=3300K)

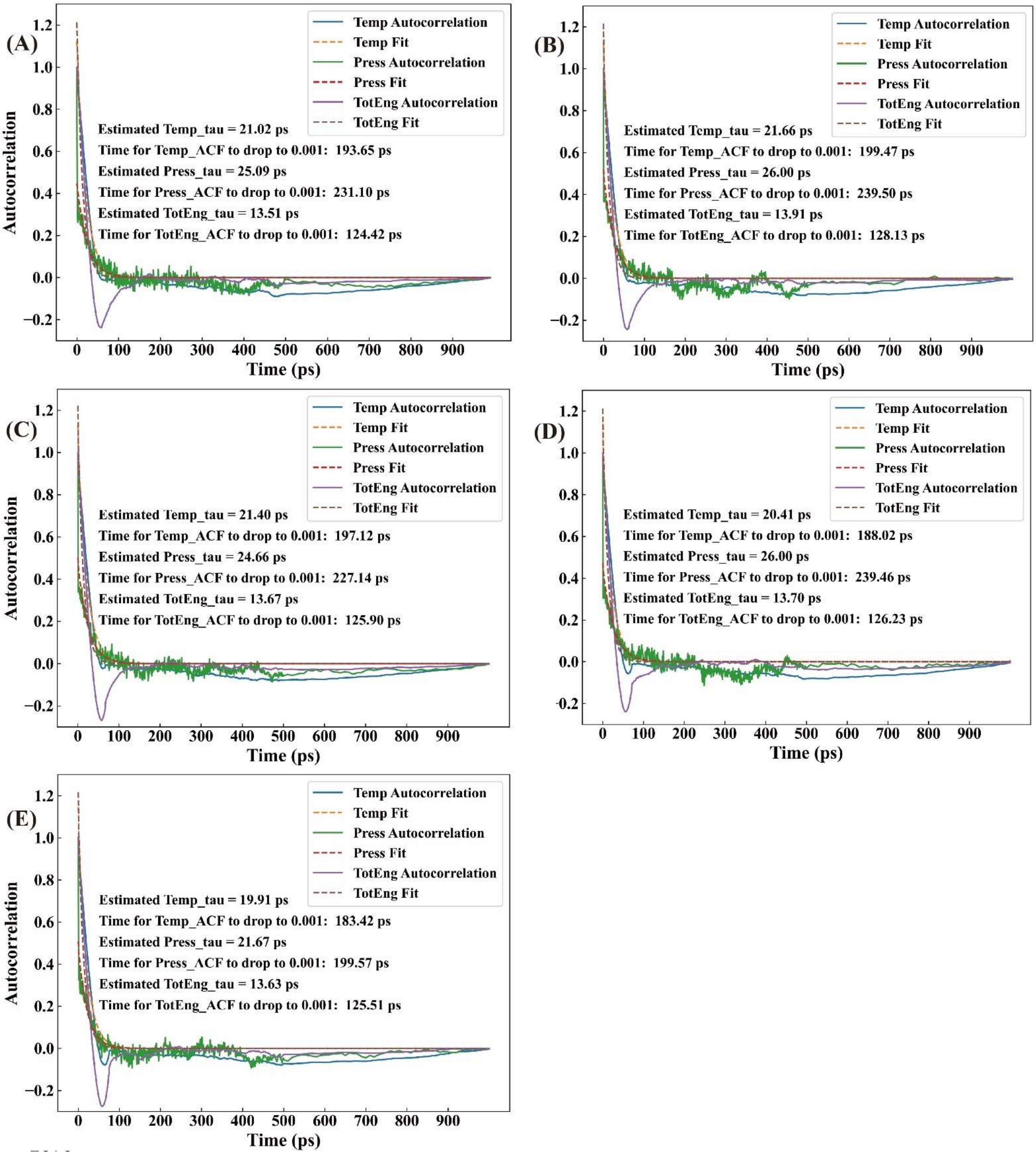

Supplementary Figure 17. Normalized time autocorrelation functions for the PTFE plasma gasification processes **under different temperature:** (A) 3300K, (B) 3400K, (C) 3600K, (D)3800K, (E) 4000K (O_2/C=2.79, H_2O/C=2.79)

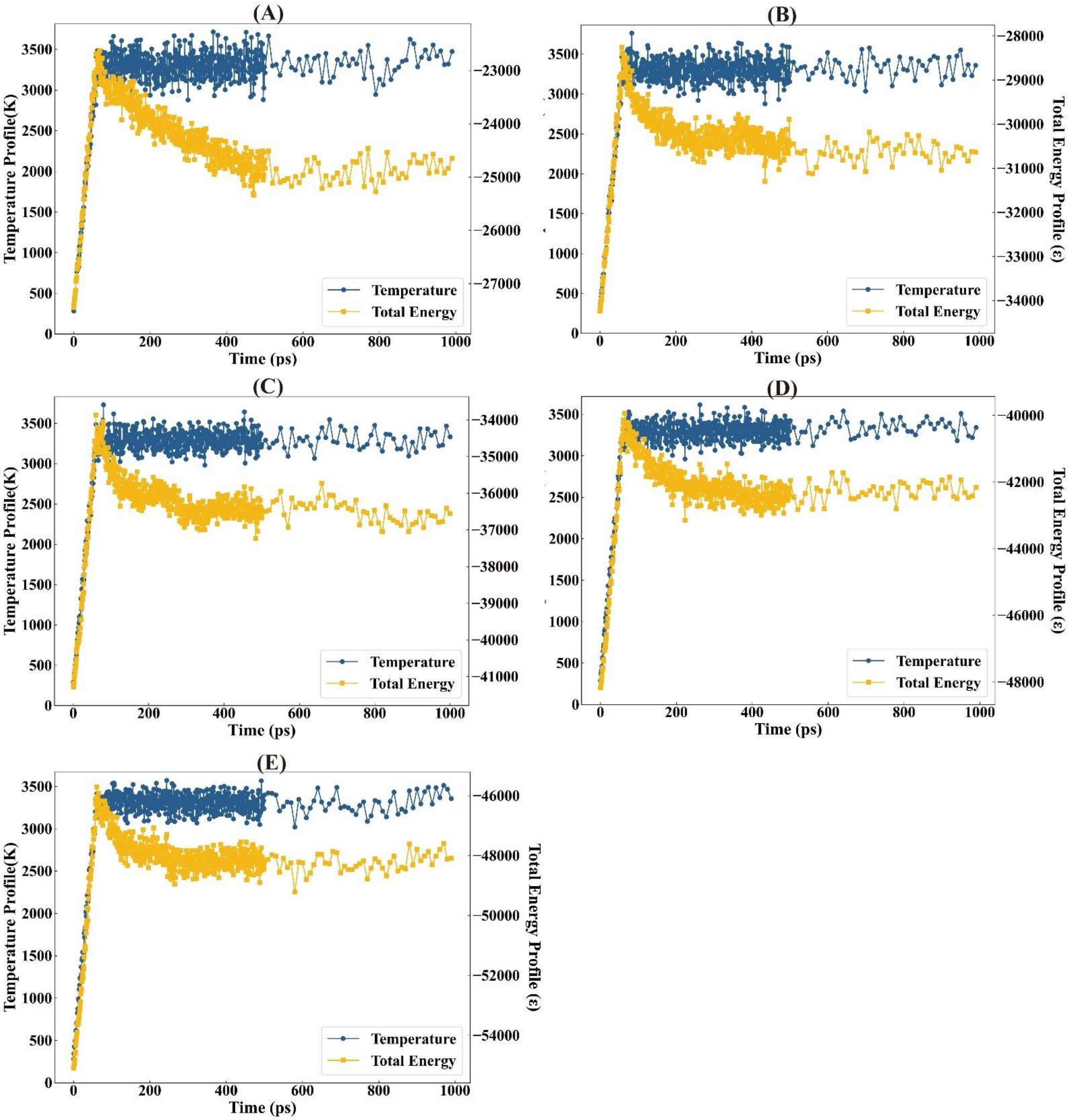

Supplementary Figure 18. The temperature and total energy profiles for the PTFE plasma gasification processes **at the O_2/C ratio of** (A) 0.93, (B)1.86, (C) 2.79, (D) 3.72, (E) 4.65 (temperature=3300K)

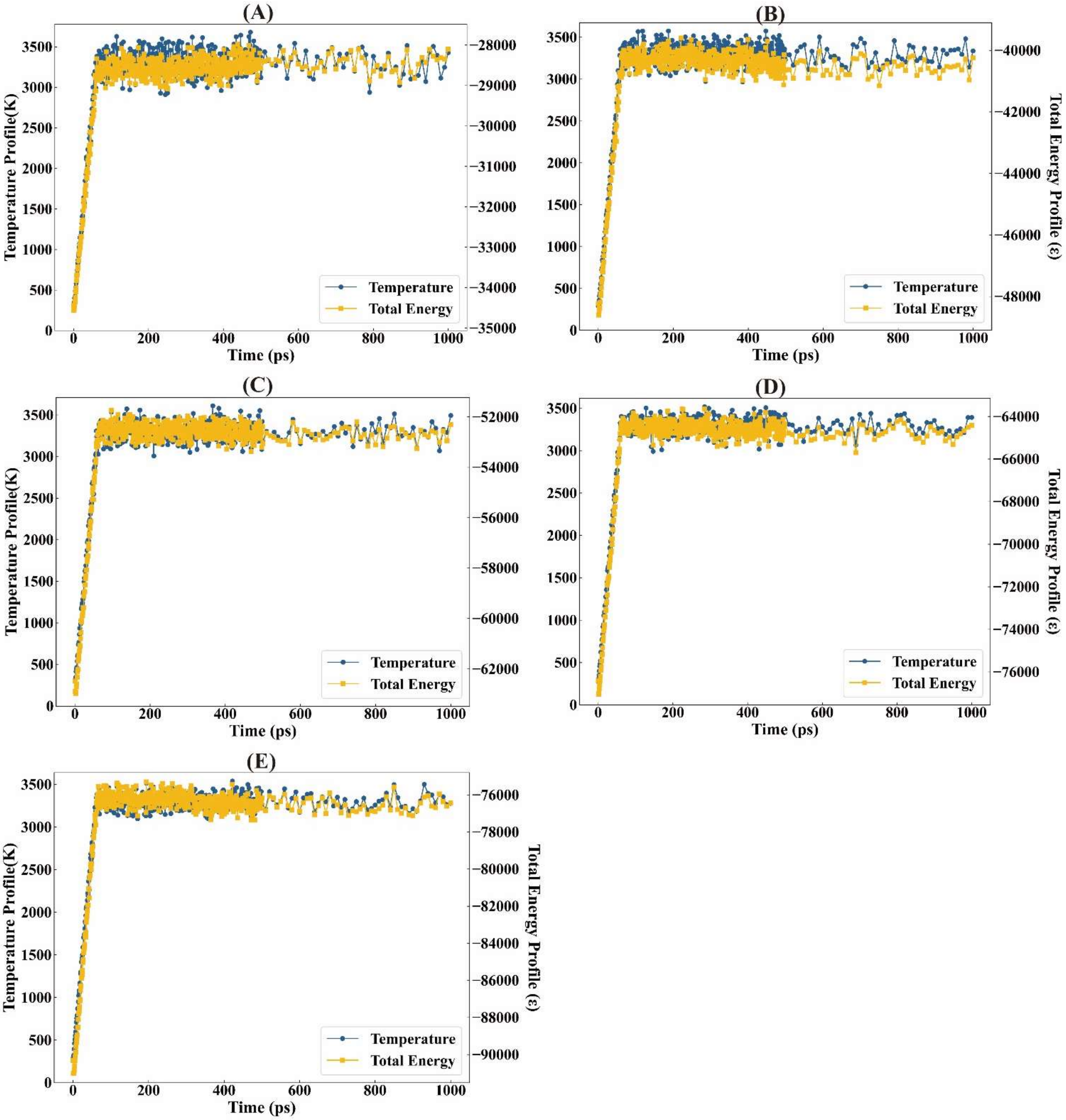

Supplementary Figure 19. The temperature and total energy profiles for the PTFE plasma gasification processes **at the H_2O/C ratio of** (A) 0.93, (B)1.86, (C) 2.79, (D) 3.72, (E) 4.65 (temperature=3300K)

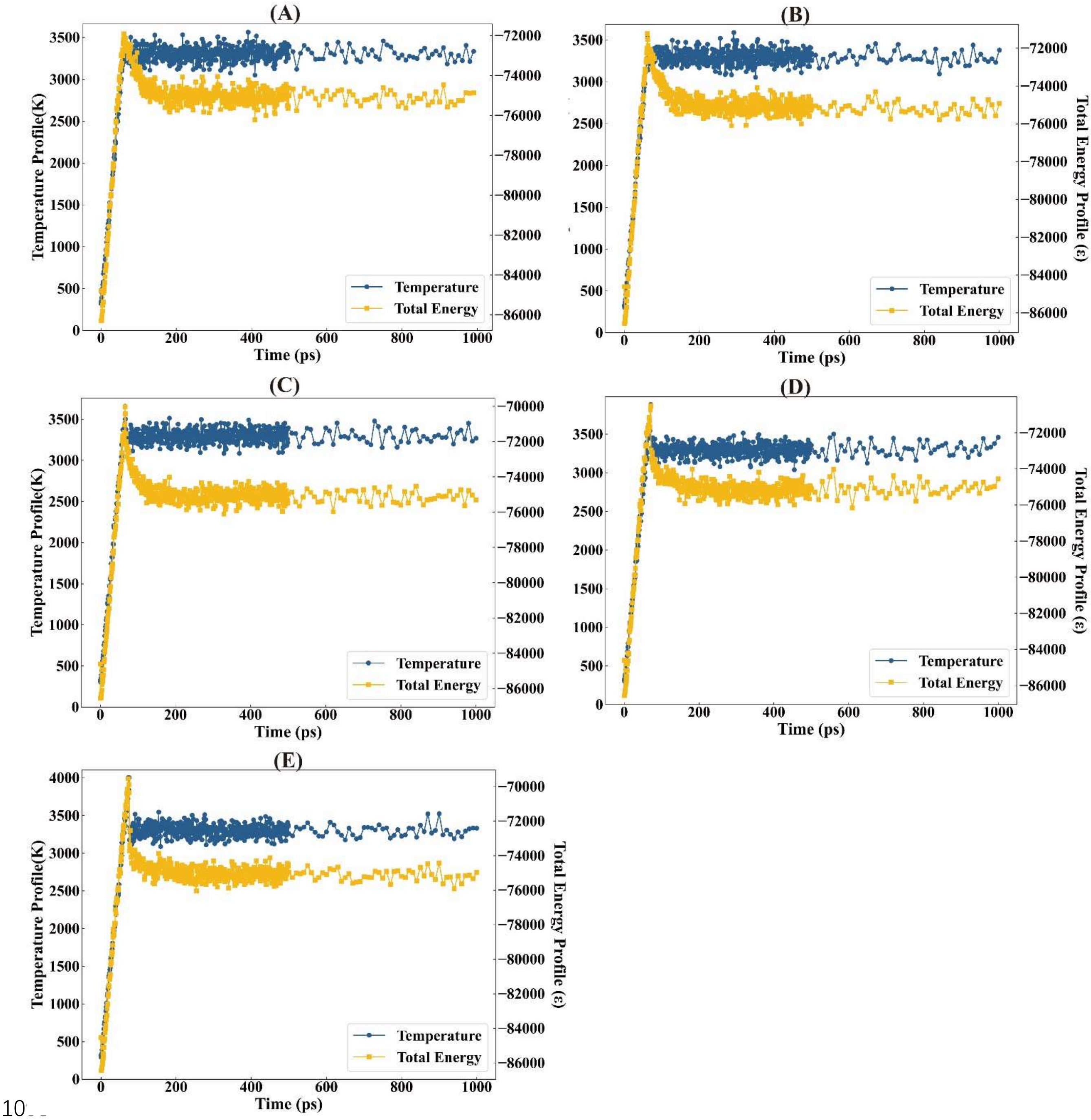

Supplementary Figure 20. The temperature and total energy profiles for the PTFE plasma gasification processes **under different temperature:** (A) 3300K, (B) 3400K, (C) 3600K, (D)3800K, (E) 4000K (O_2/C=2.79, H_2O/C=2.79)

(a)

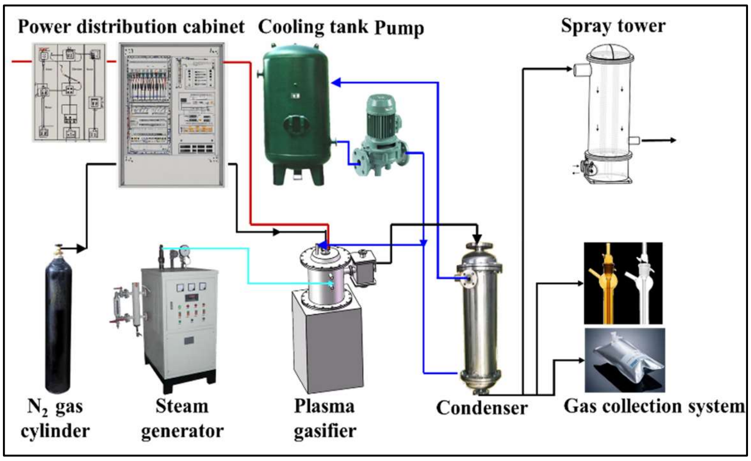

(b)

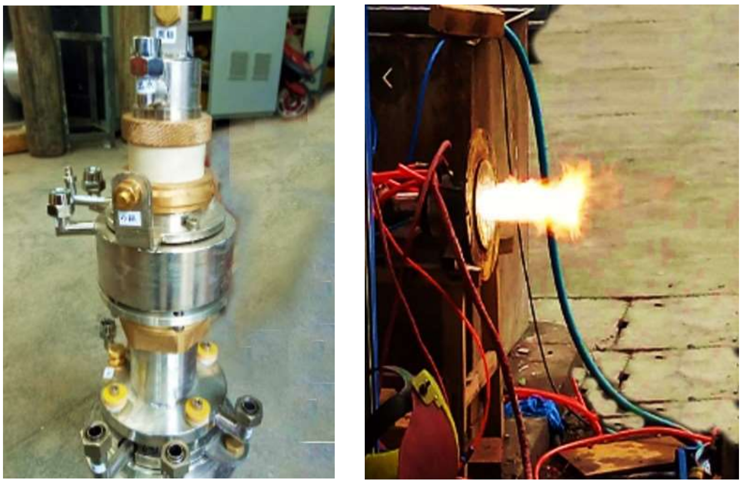

Supplementary Figure 21. The schematic diagram of plasma gasification device: (a) Schematic diagram of plasma gasifier. (b) The ignition of plasma torch (up to 30 kW), which is reproduced from

Supplementary Table 11 Targeted PFAS analytes

Chemical Name	Acronym	Molecular Formula	CAS-No.
Perfluorooctanoic Acid	PFOA	$C_8F_{15}O_2H$	335-67-1
Perfluorooctanoyl fluoride	PFOA-F	$C_8F_{16}O$	335-66-0
Ammonium pentadecafluorooctanoate	APFO	$C_8H_4F_{15}O_2N$	3825-26-1
1H,1H,2H,2H-Perfluorodecan-1-ol	8:2 FTOH	$C_{10}F_{17}OH_5$	678-39-7
1H,1H,2H,2H-Perfluorodecylacrylate	8:2 FTA	$C_{13}F_{17}O_2H_7$	27905-45-9
Methyl perfluorooctanoate	Me-PFOA	$C_9F_{15}O_2H_3$	376-27-2
1H,1H,2H,2H-Perfluorodecyl methacrylate	8:2 FTMA	$C_{14}H_9F_{17}O_2$	1996-88-9
Ethyl perfluorooctanoate	Et-PFOA	$C_{10}F_{15}O_2H_5$	3108-24-5
Perfluorobutane Acid	PFBA	$C_4F_7O_2H$	375-22-4
Perfluorobutane Sulfonate	PFBS	$C_4F_9O_3SH$	375-73-5
Perfluoropentane Acid	PFPA	$C_5F_9O_2H$	2706-90-3
Perfluorohexane Acid	PFHxA	$C_6F_{11}O_2H$	307-24-4
Perfluoroheptane Acid	PFHpA	$C_7F_{13}O_2H$	375-85-9
Perfluorononane Acid	PFNA	$C_9F_{17}O_2H$	375-95-1
Perfluorodecane Acid	PFDA	$C_{10}F_{19}O_2H$	335-76-2
Perfluoroundecanoic Acid	PFUnA	$C_{11}F_{21}O_2H$	2058-94-8
Perfluorododecanoic Acid	PFDoA	$C_{12}F_{23}O_2H$	307-55-1
Perfluorotridecanoic Acid	PFTrA	$C_{13}F_{25}O_2H$	72629-94-8
Perfluorotetradecanoic Acid	PFTeA	$C_{14}F_{27}O_2H$	376-06-7
Perfluoro-3,7-dimethyloctanoic Acid	PF-3,7-DMOA	$C_{10}F_{19}O_2H$	172155-07-6
7H-Dodecanefluoroheptane Acid	HPFHpA	$C_7F_{12}O_2H_2$	1546-95-8
2H,2H-Perfluorodecane Acid	H2PFDA	$C_{10}F_{17}O_2H_3$	27854-31-5
2H,2H,3H,3H-Perfluoroundecanoic Acid	H4PFUnA	$C_{11}F_{17}O_2H_5$	34598-33-9
Perfluorooctanesulphonic acid	H4PFOS; 6:2	$C_8H_5F_{13}O_3S$	27619-97-2
1H,1H,2H,2H-Perfluorooctylacrylate	6:2 FTA	$C_{11}H_7F_{13}O_2$	17527-29-6
1H,1H,2H,2H-Perfluorododecylacrylate	10:2 FTA	$C_{15}H_7F_{21}O_2$	17741-60-5
1H,1H,2H,2H-Perfluoro-1-hexanol	4:2 FTOH	$C_6H_5F_9O$	2043-47-2
1H,1H,2H,2H-Perfluoro-1-octanol	6:2 FTOH	$C_8H_5F_{13}O$	647-42-7
1H,1H,2H,2H-Perfluoro -1-dodecanol	10:2 FTOH	$C_{12}H_5F_{21}O$	865-86-1
Perfluorononanoate ammounium salt	APFN	$C_9H_4F_{17}NO_2$	4149-60-4
Perfluorodecanoate ammonium salt	APFDA	$C_{10}H_4F_{19}NO_2$	3108-42-7

Supplementary References

1. van der Horst R, Verreycken T, Veldhuizen E, Bruggeman P. Time-resolved optical emission spectroscopy of nanosecond pulsed discharges in atmospheric-pressure N2 and N2/H2O mixtures. *Journal of Physics D-applied Physics - J PHYS-D-APPL PHYS* **45**, (2012).

2. Fierro A, Laity G, Neuber A. Optical emission spectroscopy study in the VUV–VIS regimes of a developing low- temperature plasma in nitrogen gas. *Journal of Physics D Applied Physics* **45**, 495202-495211 (2012).

3. Minesi N, Blanchard V, Pannier E, Stancu G, Laux C. Plasma-assisted combustion with nanosecond discharges. Part I: discharge effects characterization in the burnt gases of a lean flame. *Plasma Sources Science and Technology* **31**, (2022).

4. LiNa W, ZhongWei LIU, AiMin ZHU, GuoLi Z, Yong XU. Numerical Simulation of ·OH and HO_2· Radicals in Dielectric Barrier Discharge Plasmas. *Acta Physica-Chimica Sinica* **24**, 1400-1404 (2008).

5. Capitelli M, Ferreira C, Gordiets B, Osipov A. Plasma Kinetics in Atmospheric Gases. *Plasma Physics and Controlled Fusion - PLASMA PHYS CONTROL FUSION* **43**, 371-372 (2001).

6. Laux C*, et al. Ionization Mechanisms In Two-Temperature Air Plasmas* (2000).

7. Ciccarino C, Savin D. Electron-Impact Ionization of Atomic Nitrogen. *Journal of Thermophysics and Heat Transfer* **33**, 1-9 (2018).

8. Wang Y, Zatsarinny O, Bartschat K. B-spline R-matrix-with-pseudostates calculations for electron-impact excitation and ionization of carbon. *Physical Review A* **87**, (2013).

9. Guan X, Zatsarinny O, Noble CJ, Bartschat K, Schneider B. Time-Dependent B-Spline R-Matrix Approach to Double Ionization of Atoms by XUV Laser Pulses. *Journal of Physics B: Atomic, Molecular and Optical Physics* **42**, (2009).

10. Kossyi IA, Kostinskiy A, Matveev AA. Kinetical scheme of the non-equilibrium nitrogen-oxygen mixtures. *Plasma Sources Science and Technology* **1**, 207-220 (1992).

11. Thomas JM, Kaufman F. An Upper Limit on the Formation of NO(X2Πr) in the Reactions + O(3P) and + at 298 K. *The Journal of Physical Chemistry* **100**, 8901-8906 (1996).

12. Shkurenkov I, Burnette D, Lempert WR, Adamovich IV. Kinetics of excited states and radicals in a nanosecond pulse discharge and afterglow in nitrogen and air*. *Plasma Sources Science and Technology* **23**, 065003 (2014).

13. Rusterholtz D, Lacoste D, Stancu G, Pai D, Laux C. Ultrafast heating and oxygen

dissociation in atmospheric pressure air by nanosecond repetitively pulsed discharges. *Journal of Physics D Applied Physics* **46**, 4010 (2013).

14. Mellouki A*, et al.* Evaluated kinetic and photochemical data for atmospheric chemistry: volume VIII – gas-phase reactions of organic species with four, or more, carbon atoms (⩾ C₄). *Atmospheric Chemistry and Physics* **21**, 4797-4808 (2021).

15. Stefanovic I, Bibinov NK, Deryugin AA, Vinogradov IP, Napartovich AP, Wiesemann K. Kinetics of ozone and nitric oxides in dielectric barrier discharges in O2/NOx and N2/O2/NOx mixtures. *Plasma Sources Science and Technology* **10**, 406 (2001).

16. Herron JT. Evaluated Chemical Kinetics Data for Reactions of N(2D), N(2P), and N2(A3Σ u+) in the Gas Phase. *Journal of Physical and Chemical Reference Data* **28**, 1453-1483 (1999).

17. Yu HG, Varandas AJC. Dynamics of H(D) + O3 reactions on a double many-body expansion potential-energy surface for ground state HO3. *Journal of the Chemical Society, Faraday Transactions* **93**, 2651-2656 (1997).

18. Tsang W, Hampson RFJJoP, Data CR. Chemical Kinetic Data Base for Combustion Chemistry. Part I. Methane and Related Compounds. **15**, 1087-1279 (1986).

19. Eichwald O, Yousfi M, Hennad A, Benabdessadok MD. Coupling of chemical kinetics, gas dynamics, and charged particle kinetics models for the analysis of NO reduction from flue gases. **82**, 4781-4794 (1997).

20. Hernandez-Lamoneda R, Salazar MR, Pack RT. Does ozone have a barrier to dissociation and recombination? *Chemical Physics Letters* **355**, 478-482 (2002).
