# Supplementary material for: Mechanistic exploration of polytetrafluoroethylene thermal plasma gasification through multiscale simulation coupled with experimental validation
Source: Nat Commun. 2024 Feb 23;15:1654. doi: 10.1038/s41467-024-45077-6 (PMC10891128; doi:10.1038/s41467-024-45077-6)
Supplement: Supplementary file 3 — Description of Additional Supplementary Files [file 41467_2024_45077_MOESM3_ESM.pdf]

Description of Additional Supplementary Files

File Name: Supplementary Data 1
Description: The LCMS spectra for the PFAS quantitative analysis.
