# Supplementary material for: Mechanistic exploration of polytetrafluoroethylene thermal plasma gasification through multiscale simulation coupled with experimental validation
Source: Nat Commun. 2024 Feb 23;15:1654. doi: 10.1038/s41467-024-45077-6 (PMC10891128; doi:10.1038/s41467-024-45077-6)

LC/MS Test Dataset

Table of Contents

Sample No.	Test Name	Description
1	KB-20220702	Mixed atmosphere (O_2/C=2.7, H_2O/C=2.7), at the Temperature of 3300K
2	WTF22F06132107C-1	Mixed atmosphere (O_2/C=2.7, H_2O/C=2.7), at the Temperature of 3300K
3	WTF22F06132107C-2	Mixed atmosphere (O_2/C=2.7, H_2O/C=2.7), at the Temperature of 3300K
4	WTF22F06132107C-3	Mixed atmosphere (O_2/C=2.7, H_2O/C=2.7), at the Temperature of 3300K
5	WTF22F06132107C-4	Mixed atmosphere (O_2/C=2.7, H_2O/C=2.7), at the Temperature of 3300K
6	WTF22F06132107C-5	Oxygen atmosphere (Temperature=3300K), at the O_2/C ratio of 0.83
7	WTF22F06132107C-6	Oxygen atmosphere (Temperature=3300K), at the O_2/C ratio of 1.86
8	WTF22F11234969C-1	Oxygen atmosphere (Temperature=3300K), at the O_2/C ratio of 2.79
9	WTF22F11234969C-2	Oxygen atmosphere (Temperature=3300K), at the O_2/C ratio of 3.72
10	WTF22F11234969C-3	Oxygen atmosphere (Temperature=3300K), at the O_2/C ratio of 4.65
11	WTF22F11234969C-4	Steam atmosphere (Temperature=3300K), at the H_2O/C ratio of 0.83
12	WTF22F11234969C-5	Steam atmosphere (Temperature=3300K), at the H_2O/C ratio of 1.86
13	WTF22F11234969C-6	Steam atmosphere (Temperature=3300K), at the H_2O/C ratio of 2.79
14	WTF22F11234969C-7	Steam atmosphere (Temperature=3300K), at the H_2O/C ratio of 3.72
15	WTF22F11234969C-8	Steam atmosphere (Temperature=3300K), at the H_2O/C ratio of 4.65

Dataset: D:\PFCs.PRO\20220704-1.qld

Last Altered: Friday, July 08, 2022 14:11:53 China Standard Time
Printed: Friday, July 08, 2022 14:12:14 China Standard Time

Method: D:\PFCs.PRO\MethDB\PFC-20211113.mdb 11 May 2022 18:44:32
Calibration: D:\PFCs.PRO\CurveDB\NO23-20220511.cdb 11 May 2022 18:54:41

Name: KB-20220702, Date: 02-Jul-2022, Time: 12:11:05, ID: , Description: KB-20220702

PFBA

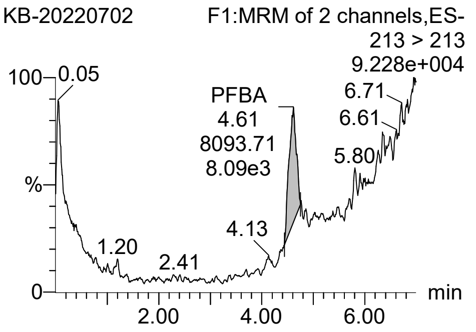

PFTeDA

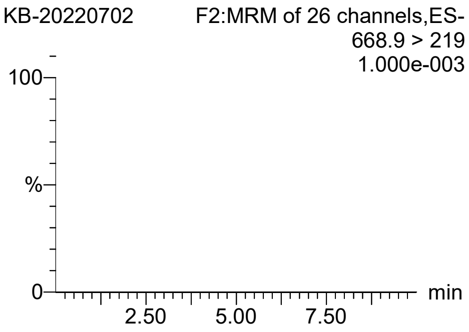

PFTrDA

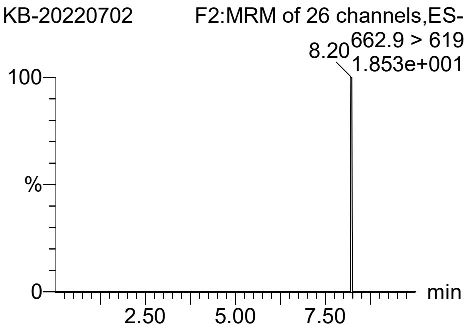

PFDoDA

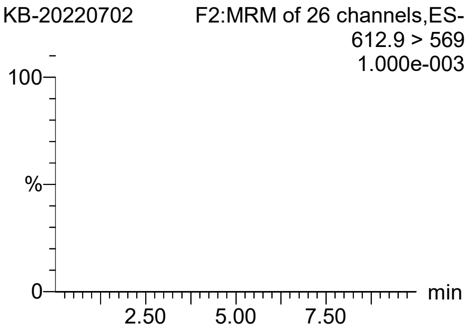

PFUNA

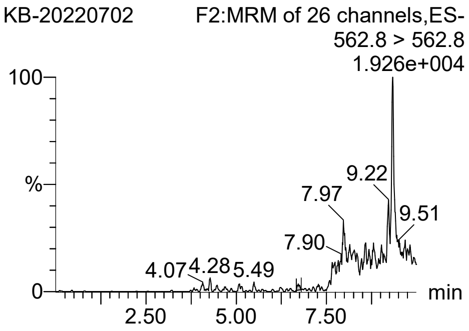

PFOS

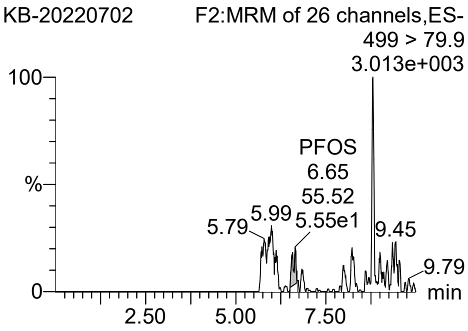

PFNA

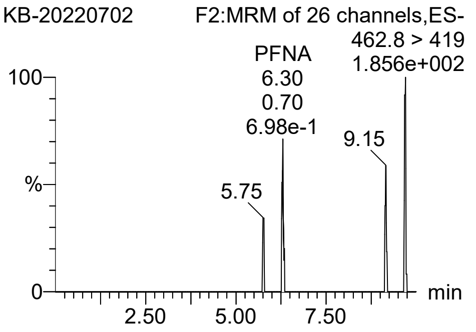

H4PFOS(6:2)

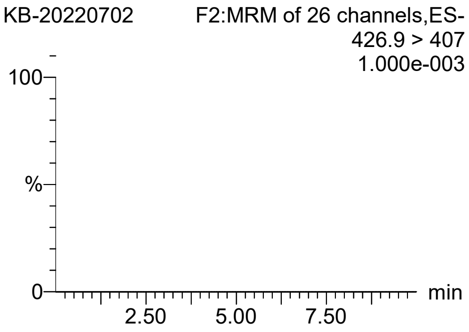

PFOA

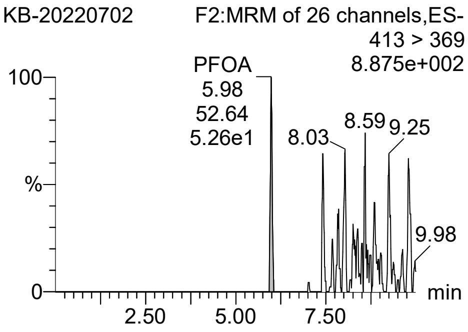

PFHPA

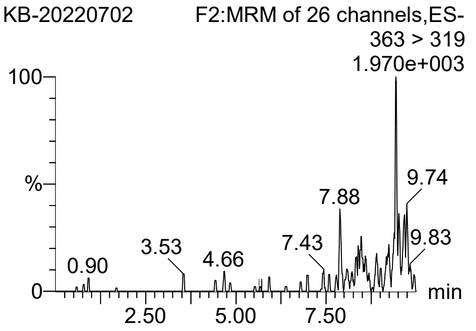

PFHxA

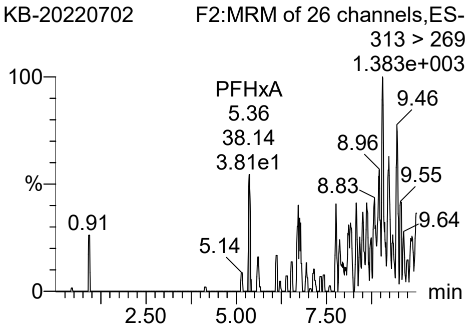

PFPA

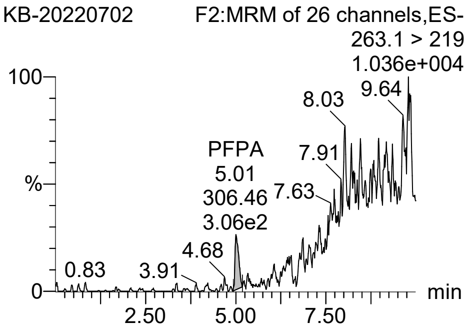

 Reviewer:

Dataset: D:\PFCs.PRO\20220704-1.qld

Last Altered: Friday, July 08, 2022 14:11:53 China Standard Time
Printed: Friday, July 08, 2022 14:12:14 China Standard Time

Name: KB-20220702, Date: 02-Jul-2022, Time: 12:11:05, ID: , Description: KB-20220702

PFDS

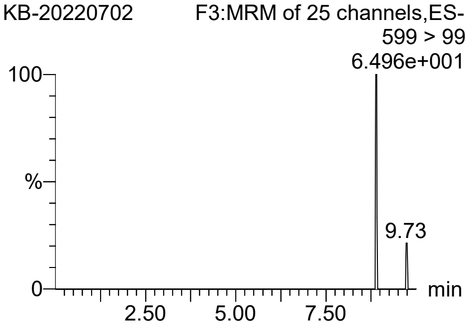

PFDA

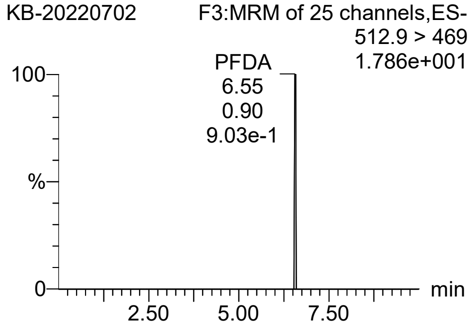

4HPFUnA

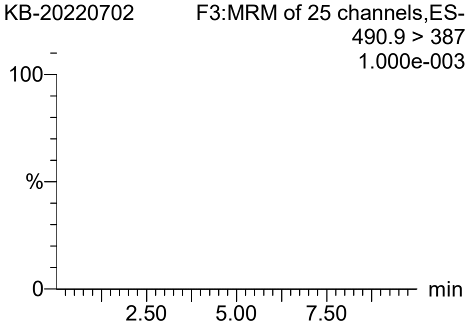

PF-3

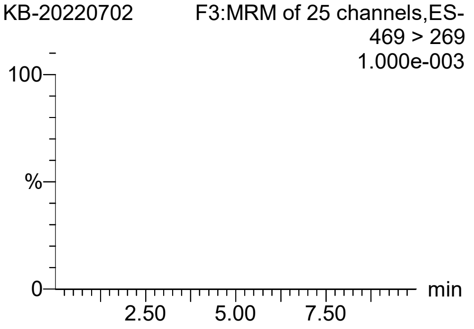

PFHpS

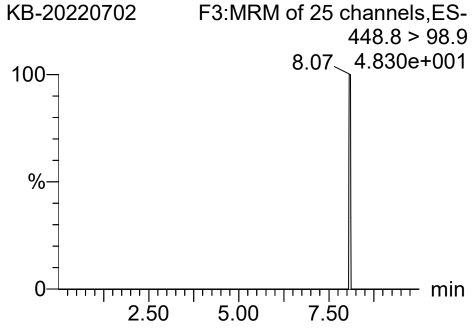

PFHxS

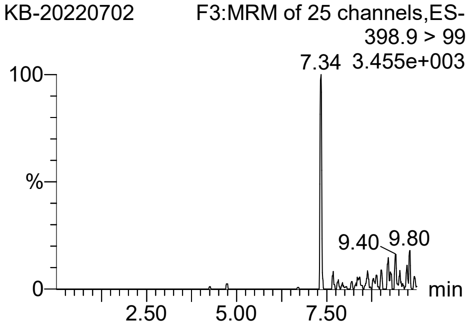

HPFHpA

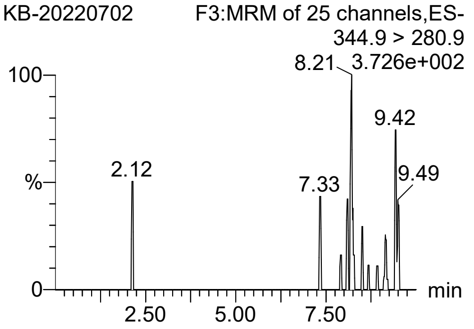

PFBS

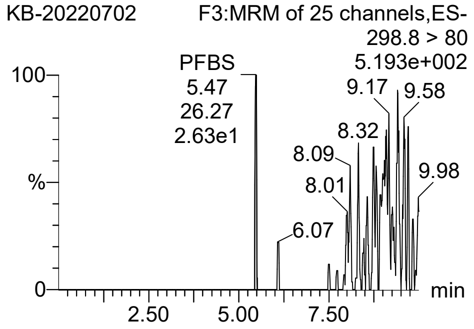

EtFOSE

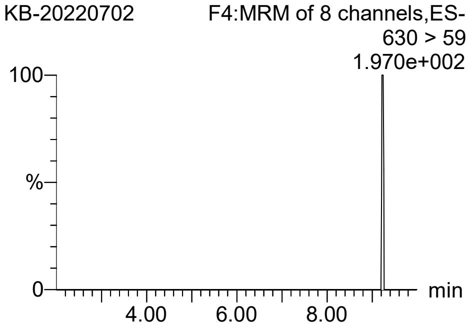

MeFOSE

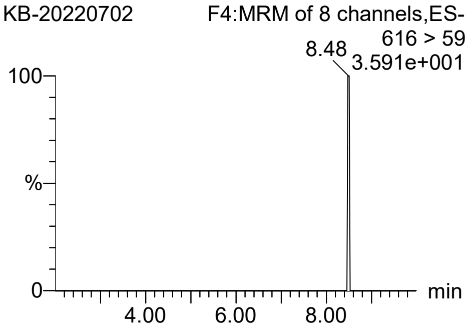

EtFOSA

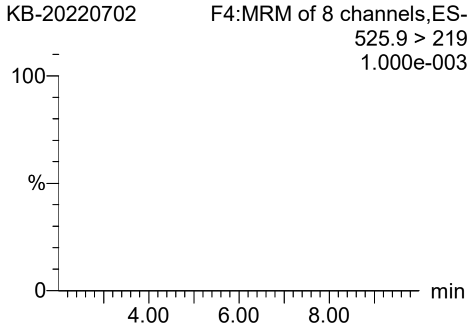

MeFOSA

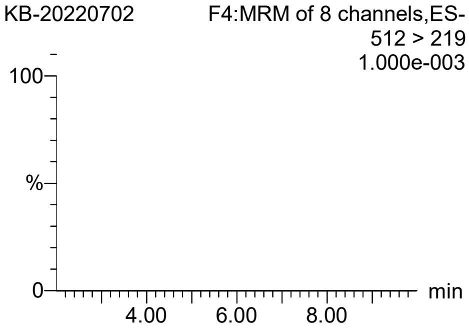

Reviewer:

Dataset: D:\PFCs.PRO\20220704-1.qld

Last Altered: Friday, July 08, 2022 14:11:53 China Standard Time
Printed: Friday, July 08, 2022 14:12:14 China Standard Time

Name: WTF22F06132107C-1, Date: 02-Jul-2022, Time: 13:16:12, ID: , Description: WTF22F06132107C-1

PFBA

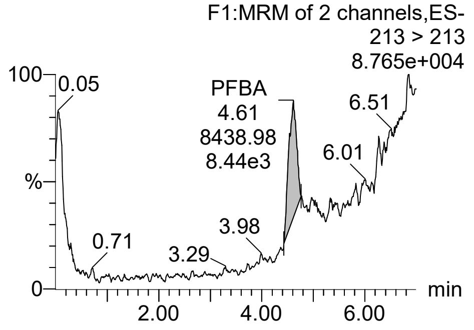

PFTeDA

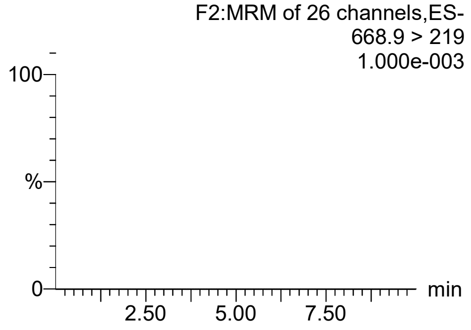

PFTrDA

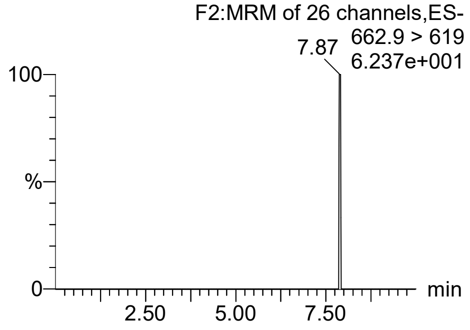

PFDoDA

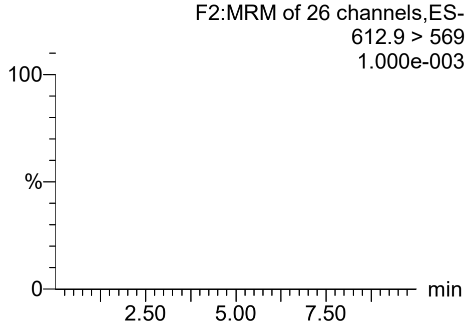

PFUNA

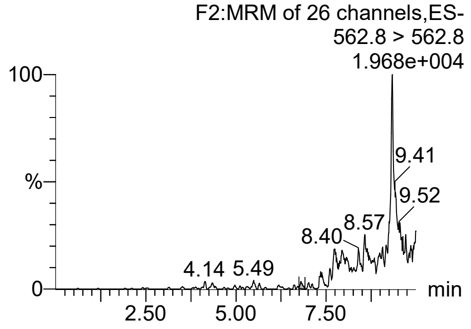

PFOS

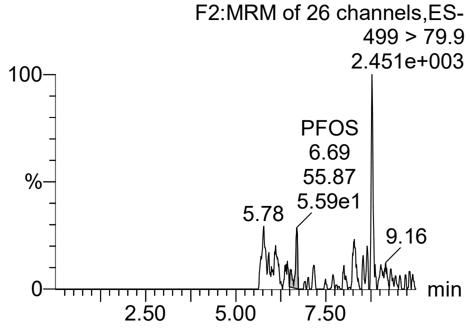

PFNA

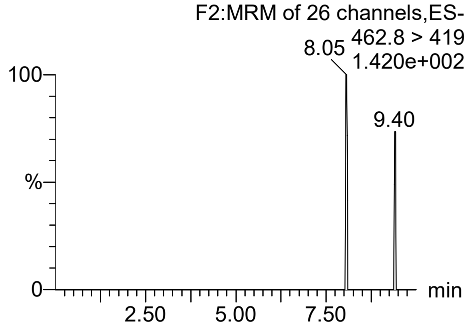

H4PFOS(6:2)

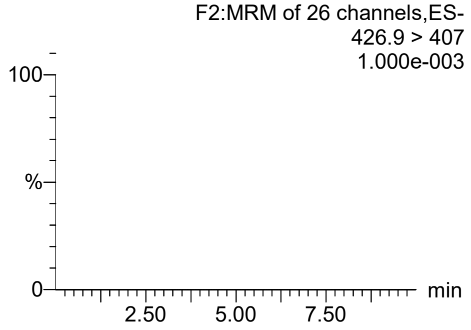

PFOA

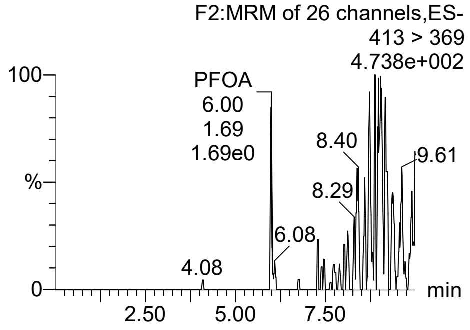

PFHPA

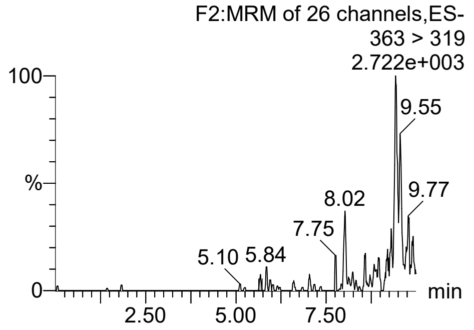

PFHxA

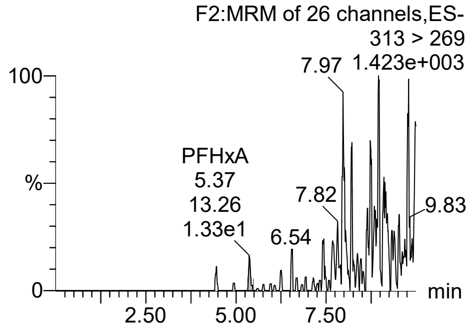

PFPA

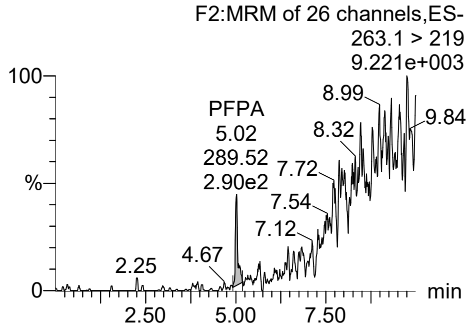

Dataset: D:\PFCs.PRO\20220704-1.qld

Last Altered: Friday, July 08, 2022 14:11:53 China Standard Time
Printed: Friday, July 08, 2022 14:12:14 China Standard Time

Name: WTF22F06132107C-1, Date: 02-Jul-2022, Time: 13:16:12, ID: , Description: WTF22F06132107C-1

PFDS

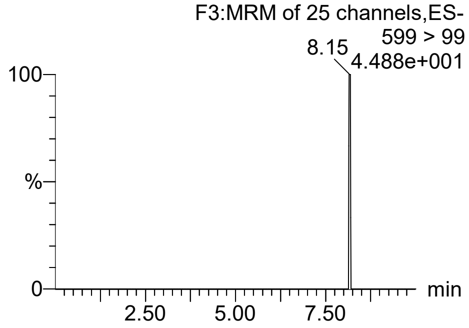

PFDA

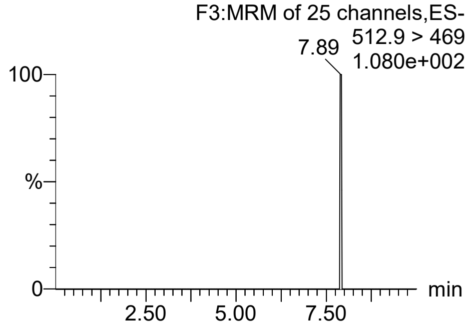

4HPFUnA

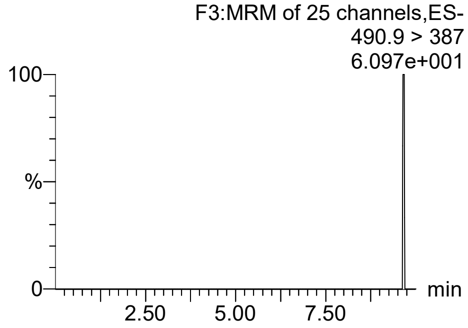

PF-3

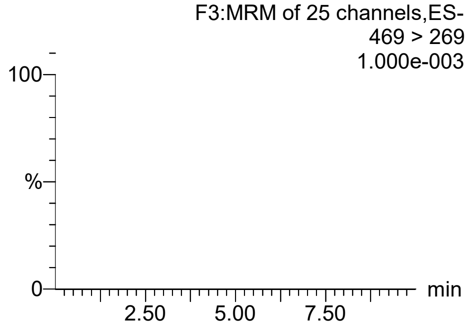

PFHpS

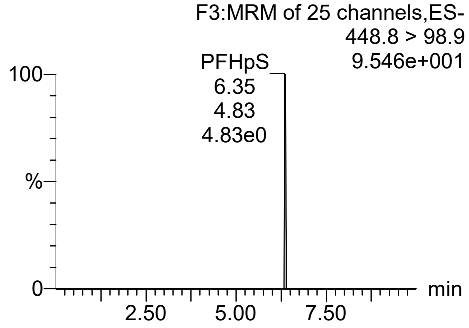

PFHxS

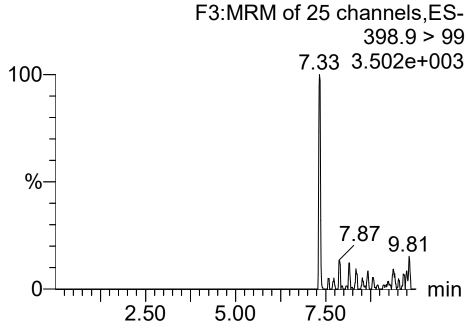

HPFHpA

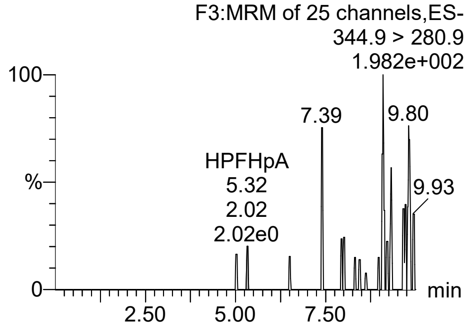

PFBS

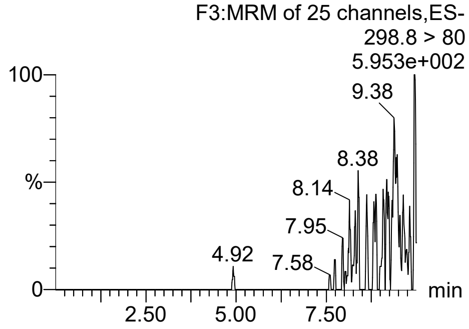

EtFOSE

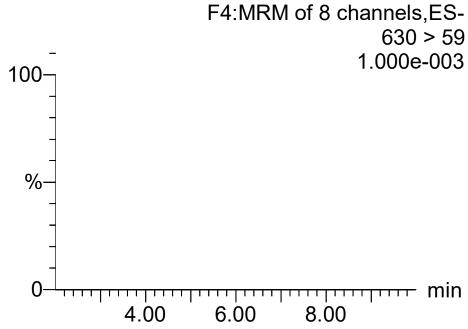

MeFOSE

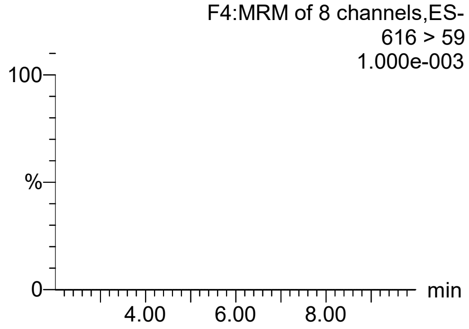

EtFOSA

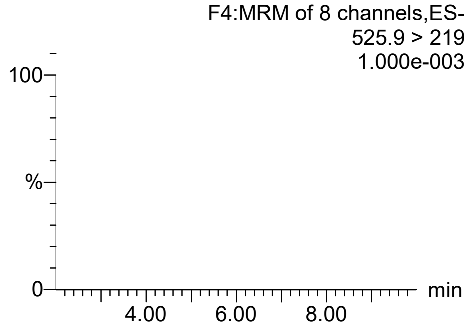

MeFOSA

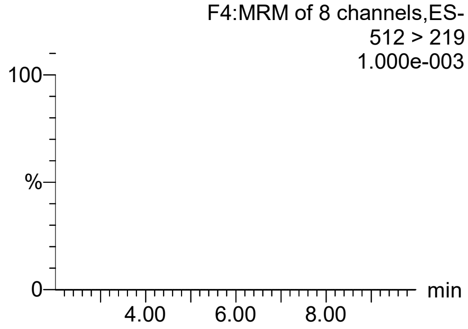

 Reviewer:

Dataset: D:\PFCs.PRO\20220704-1.qld

Last Altered: Friday, July 08, 2022 14:11:53 China Standard Time
Printed: Friday, July 08, 2022 14:12:14 China Standard Time

Name: WTF22F06132107C-2, Date: 02-Jul-2022, Time: 13:29:11, ID: , Description: WTF22F06132107C-2

PFBA

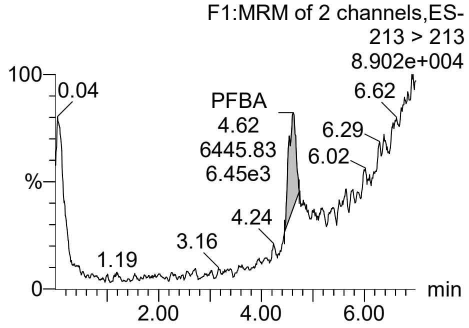

PFTeDA

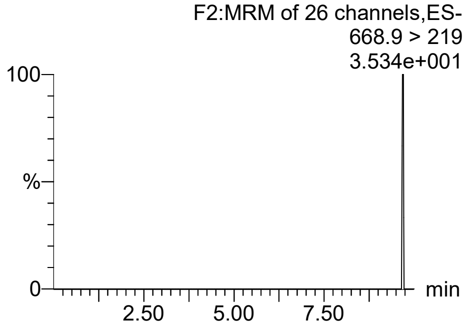

PFTrDA

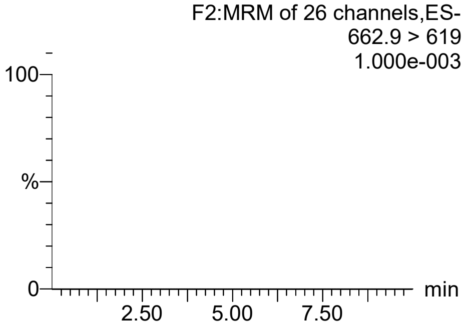

PFDoDA

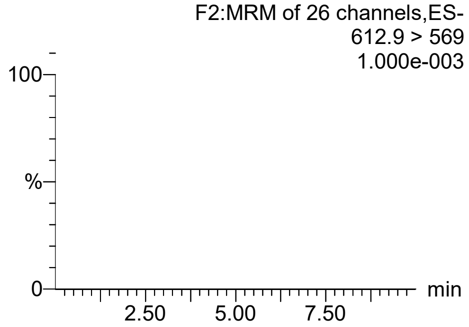

PFUNA

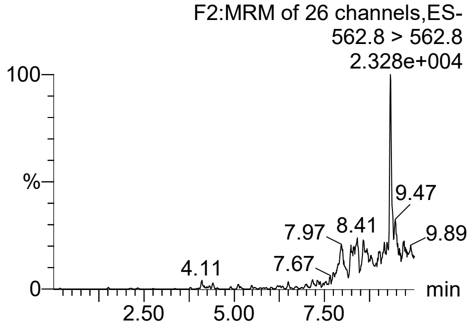

PFOS

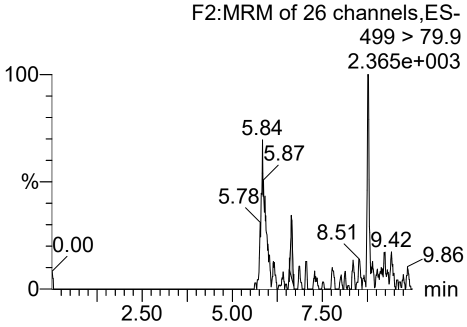

PFNA

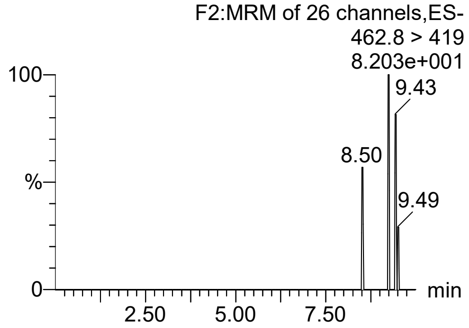

H4PFOS(6:2)

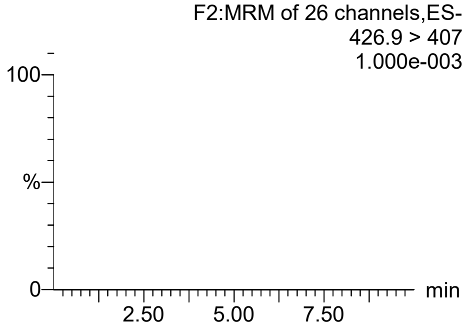

PFOA

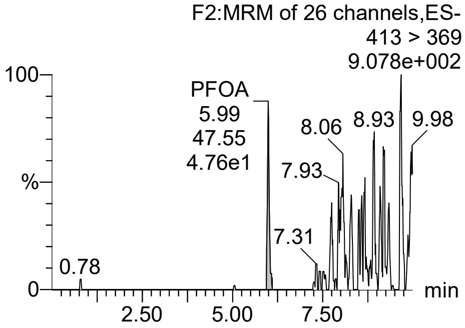

PFHPA

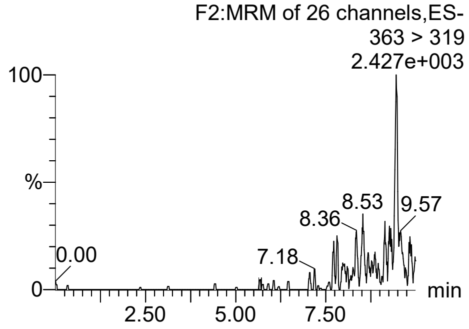

PFHxA

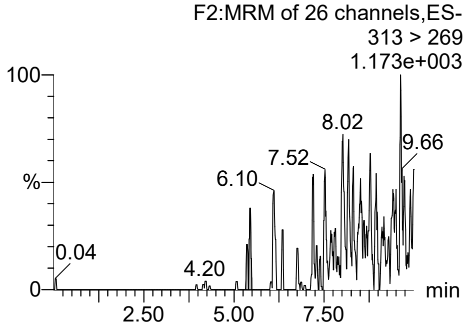

PFPA

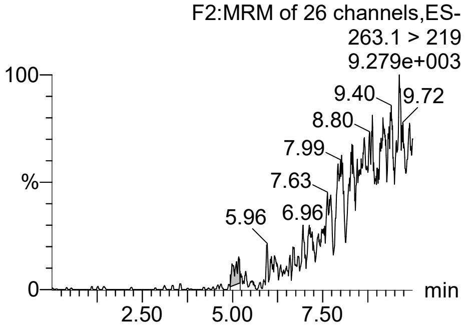

Dataset: D:\PFCs.PRO\20220704-1.qld

Last Altered: Friday, July 08, 2022 14:11:53 China Standard Time
Printed: Friday, July 08, 2022 14:12:14 China Standard Time

Name: WTF22F06132107C-2, Date: 02-Jul-2022, Time: 13:29:11, ID: , Description: WTF22F06132107C-2

PFDS

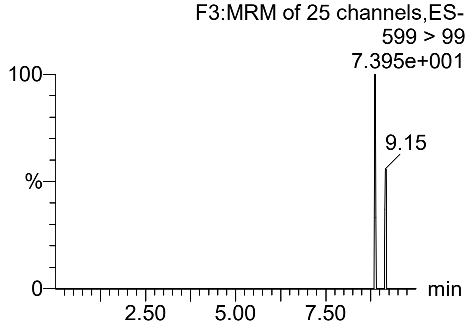

PFDA

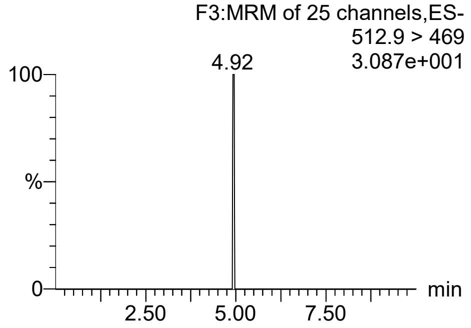

4HPFUnA

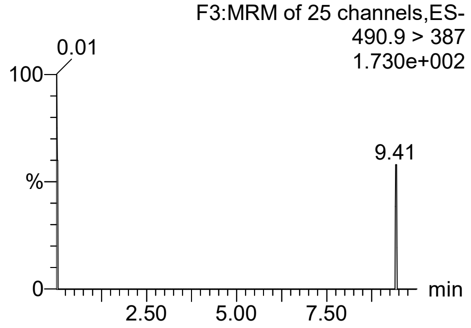

PF-3

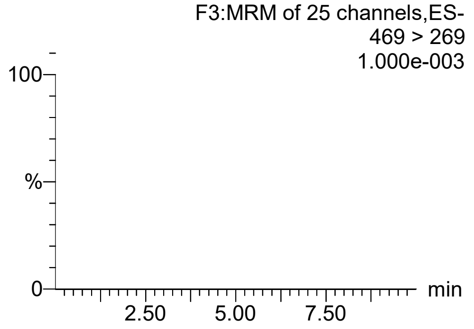

PFHpS

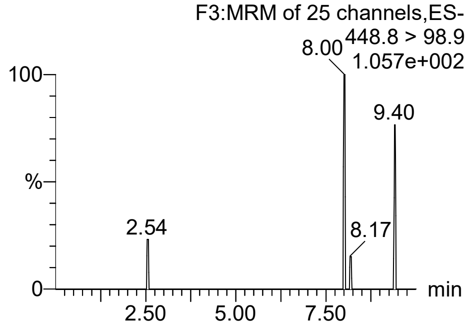

PFHxS

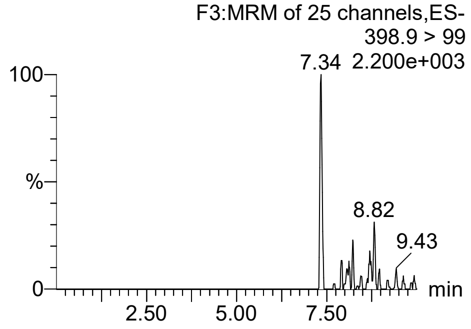

HPFHpA

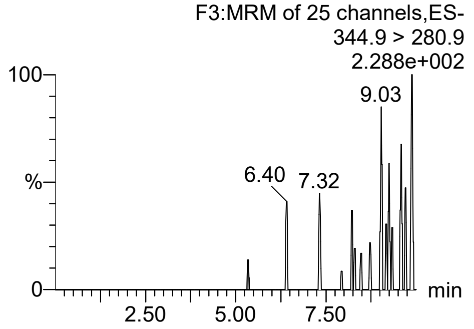

PFBS

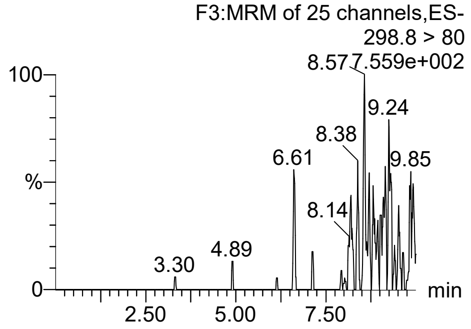

EtFOSE

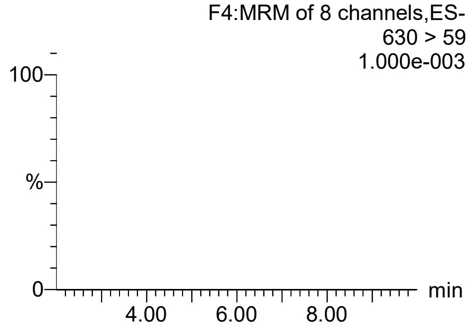

MeFOSE

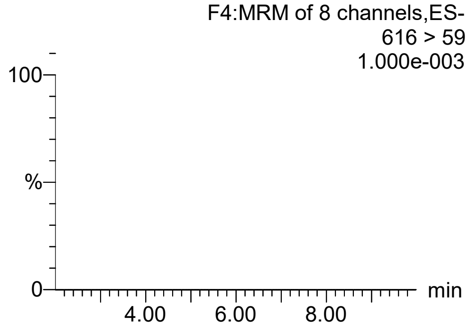

EtFOSA

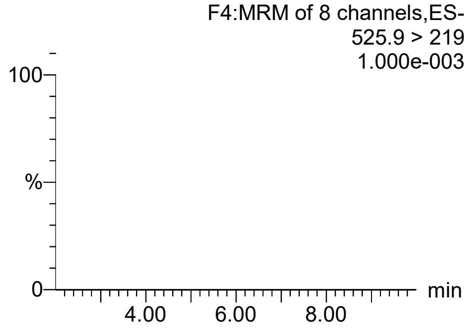

MeFOSA

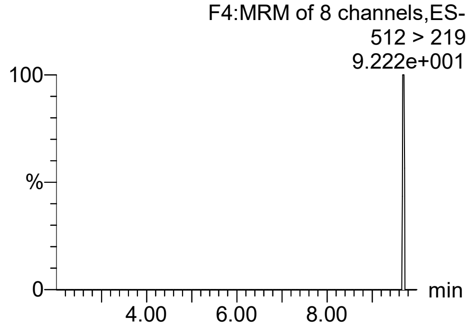

Dataset: D:\PFCs.PRO\20220704-1.qld

Last Altered: Friday, July 08, 2022 14:11:53 China Standard Time
Printed: Friday, July 08, 2022 14:12:14 China Standard Time

Name: WTF22F06132107C-3, Date: 02-Jul-2022, Time: 13:42:11, ID: , Description: WTF22F06132107C-3

PFBA

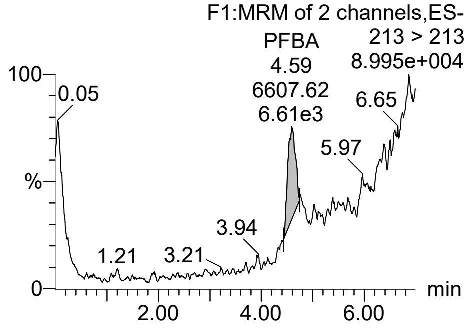

PFTeDA

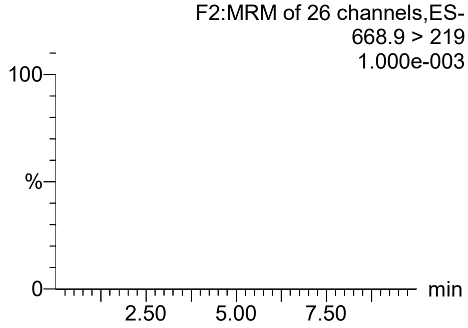

PFTrDA

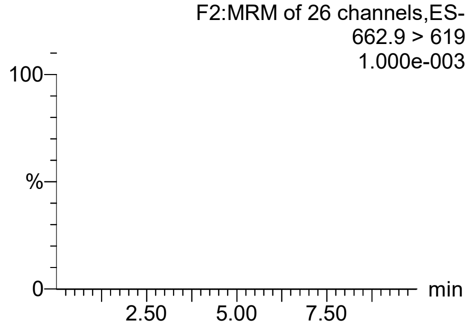

PFDoDA

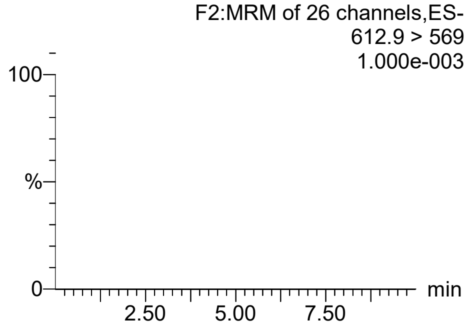

PFUNA

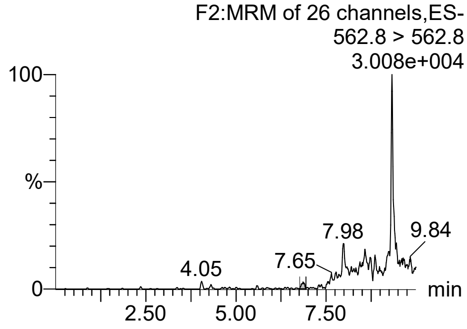

PFOS

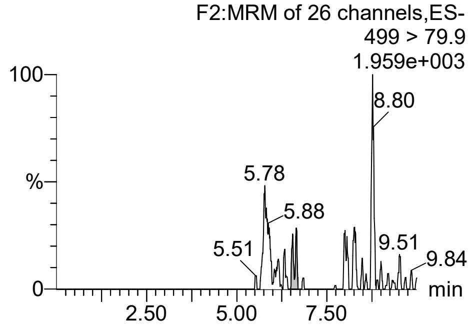

PFNA

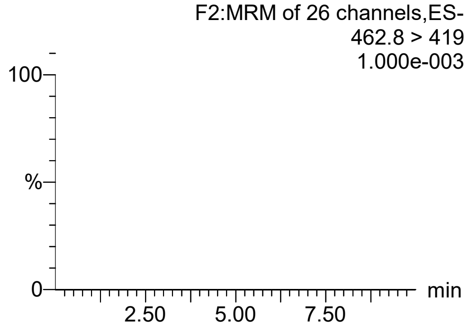

H4PFOS(6:2)

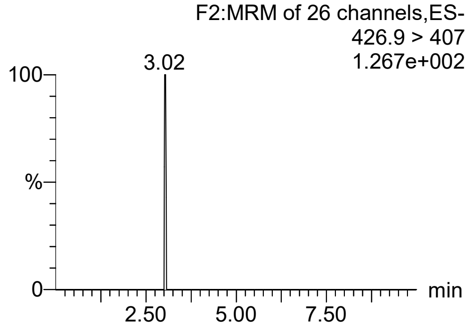

PFOA

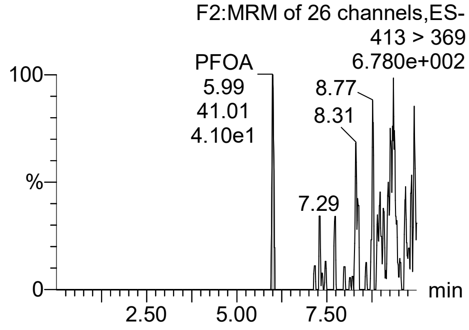

PFHPA

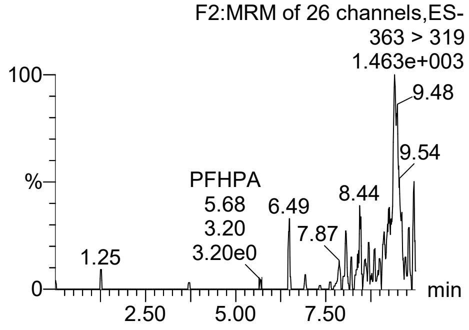

PFHxA

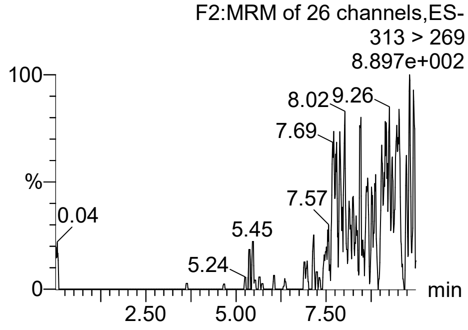

PFPA

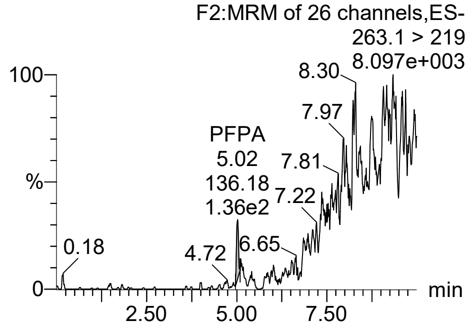

 Reviewer:

Dataset: D:\PFCs.PRO\20220704-1.qld

Last Altered: Friday, July 08, 2022 14:11:53 China Standard Time
Printed: Friday, July 08, 2022 14:12:14 China Standard Time

Name: WTF22F06132107C-3, Date: 02-Jul-2022, Time: 13:42:11, ID: , Description: WTF22F06132107C-3

PFDS

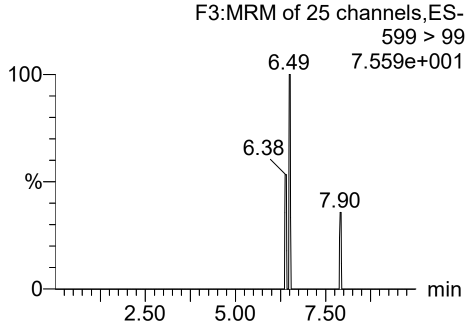

PFDA

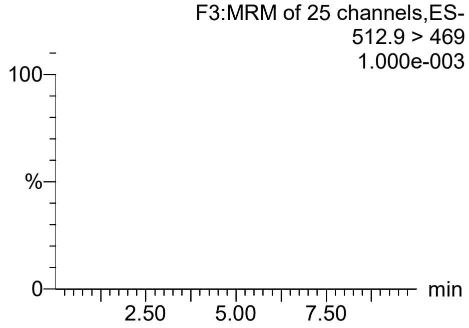

4HPFUnA

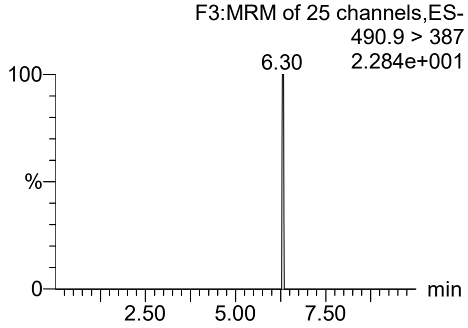

PF-3

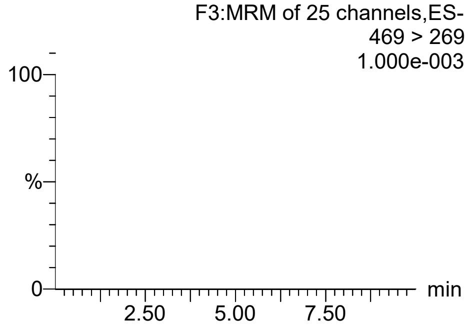

PFHpS

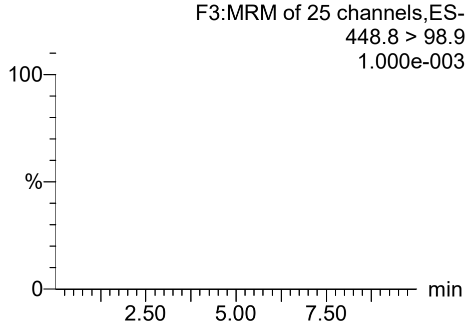

PFHxS

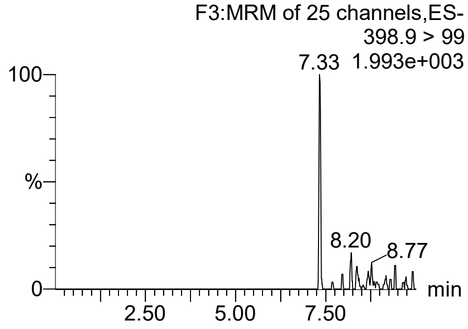

HPFHpA

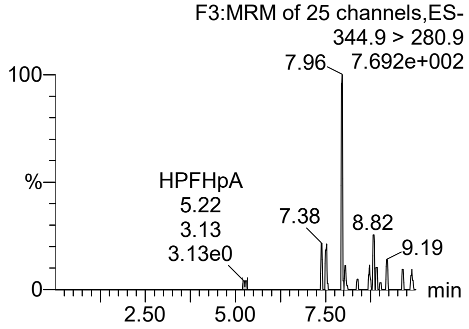

PFBS

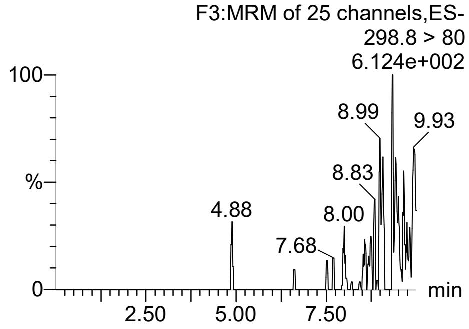

EtFOSE

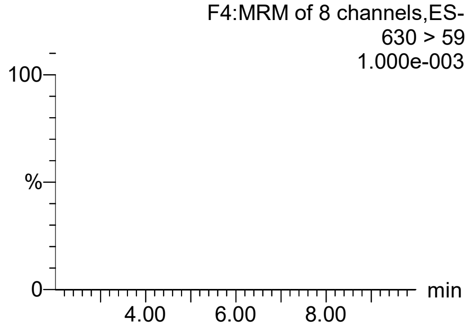

MeFOSE

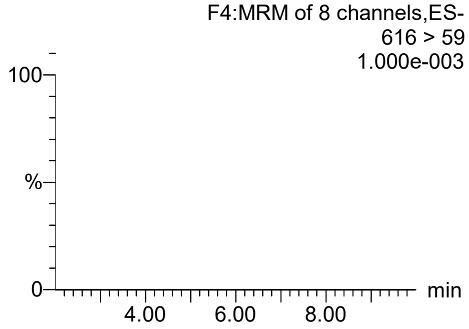

EtFOSA

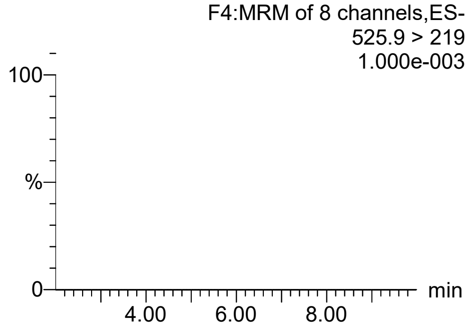

MeFOSA

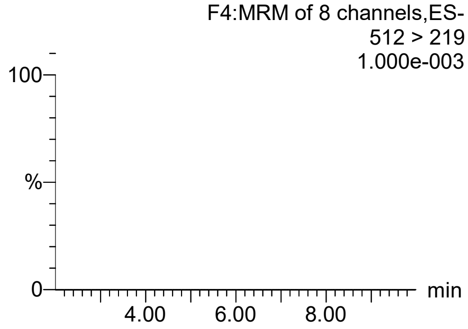

Reviewer:

Dataset: D:\PFCs.PRO\20220704-1.qld

Last Altered: Friday, July 08, 2022 14:11:53 China Standard Time
Printed: Friday, July 08, 2022 14:12:14 China Standard Time

Name: WTF22F06132107C-4, Date: 02-Jul-2022, Time: 13:55:11, ID: , Description: WTF22F06132107C-4

PFBA

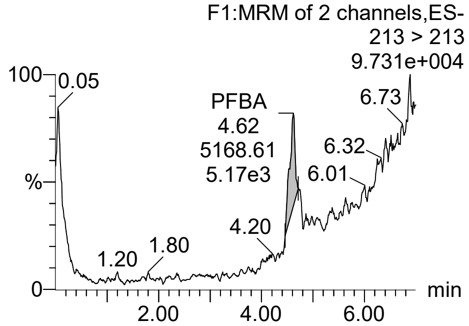

PFTeDA

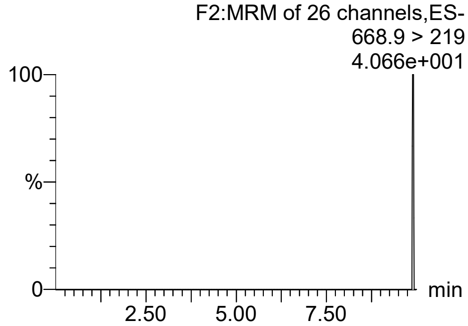

PFTrDA

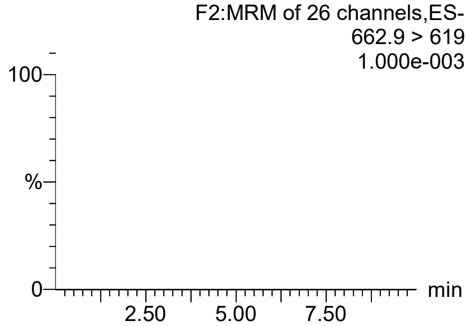

PFDoDA

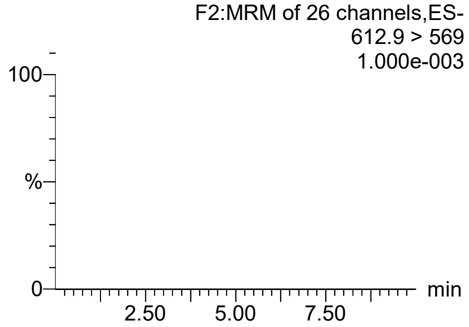

PFUNA

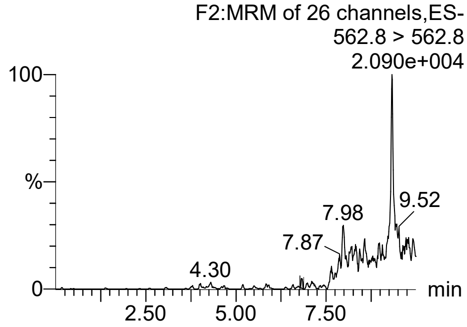

PFOS

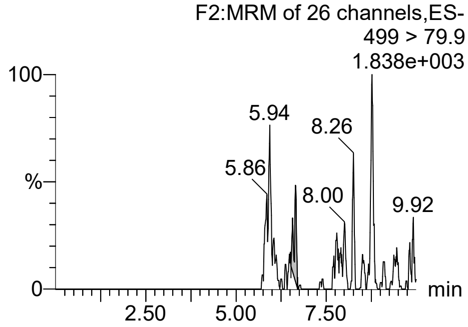

PFNA

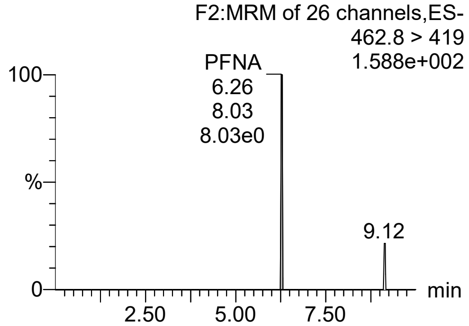

H4PFOS(6:2)

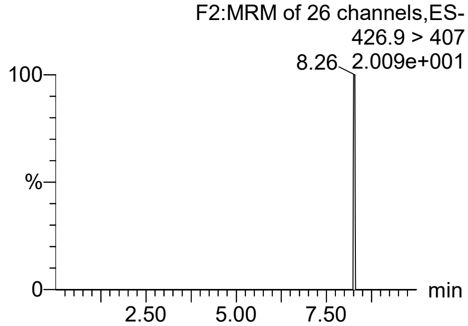

PFOA

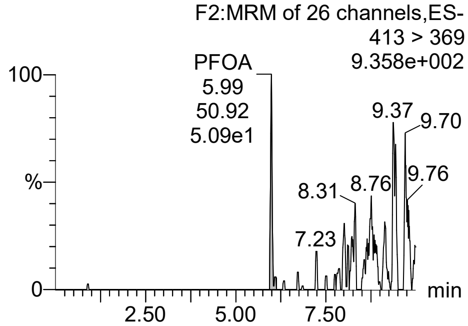

PFHPA

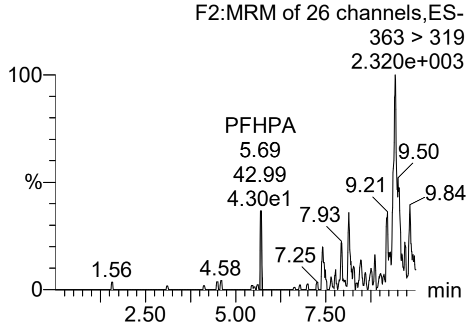

PFHxA

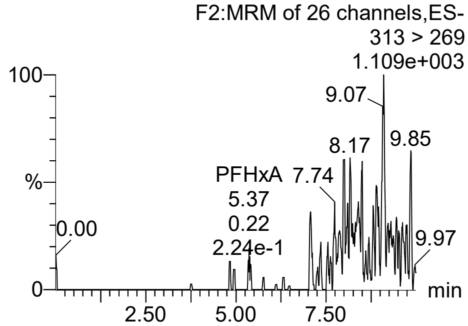

PFPA

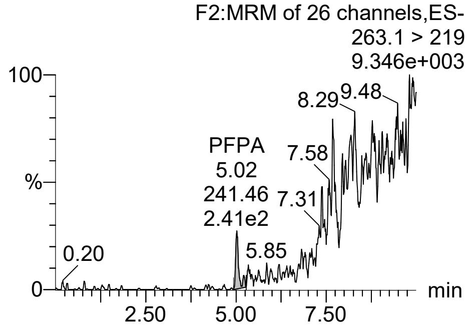

 Reviewer:

Dataset: D:\PFCs.PRO\20220704-1.qld

Last Altered: Friday, July 08, 2022 14:11:53 China Standard Time
Printed: Friday, July 08, 2022 14:12:14 China Standard Time

Name: WTF22F06132107C-4, Date: 02-Jul-2022, Time: 13:55:11, ID: , Description: WTF22F06132107C-4

PFDS

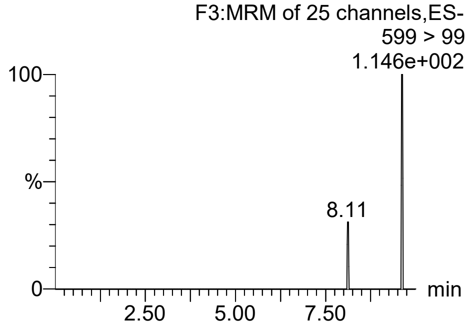

PFDA

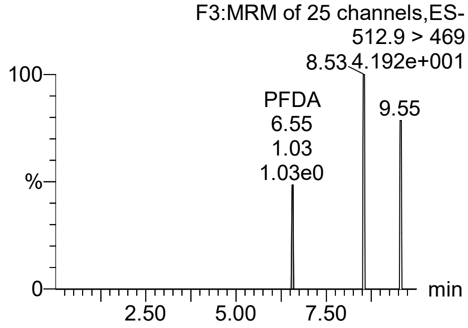

4HPFUnA

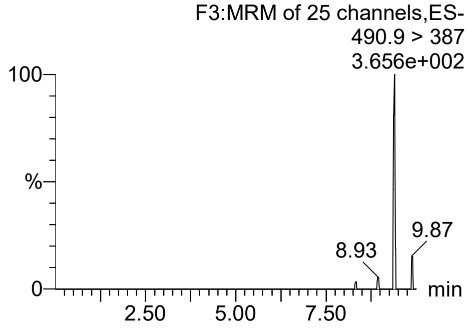

PF-3

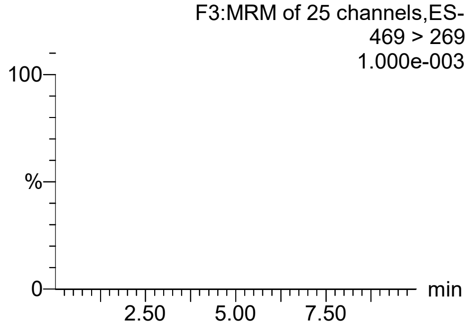

PFHpS

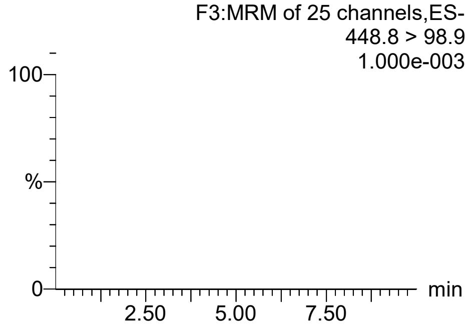

PFHxS

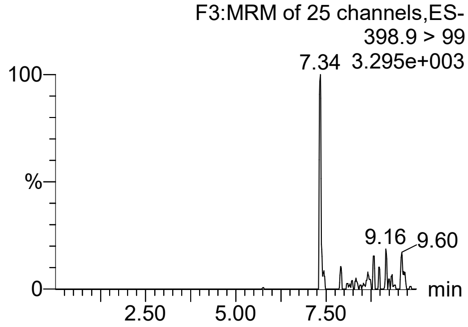

HPFHpA

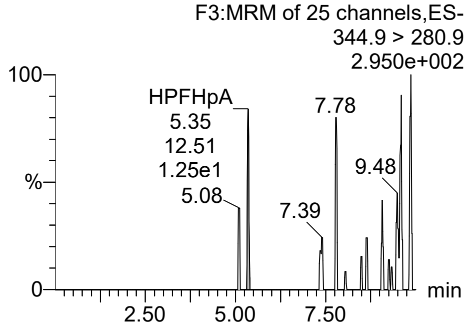

PFBS

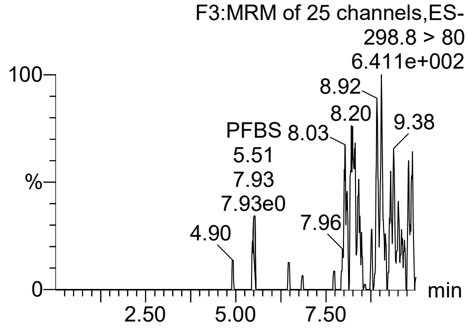

EtFOSE

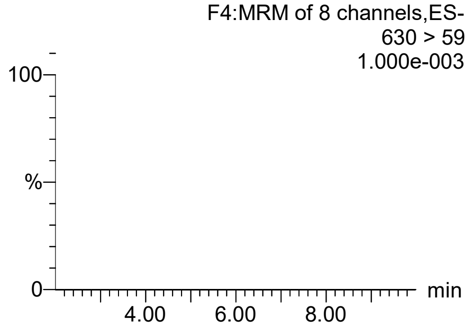

MeFOSE

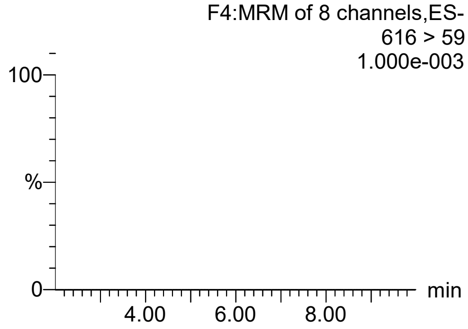

EtFOSA

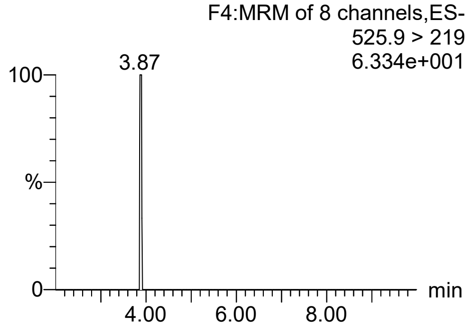

MeFOSA

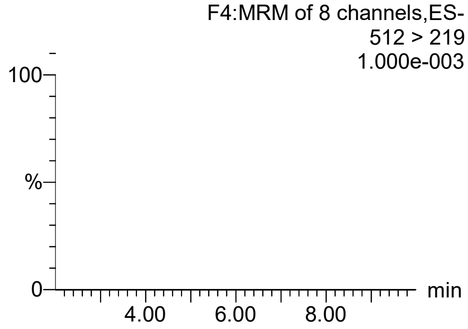

Dataset: D:\PFCs.PRO\20220704-1.qld

Last Altered: Friday, July 08, 2022 14:11:53 China Standard Time
Printed: Friday, July 08, 2022 14:12:14 China Standard Time

Name: WTF22F06132107C-5, Date: 02-Jul-2022, Time: 14:08:10, ID: , Description: WTF22F06132107C-5

PFBA

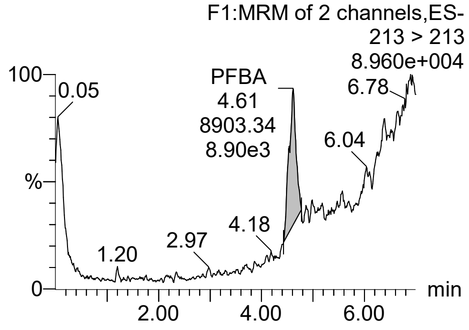

PFTeDA

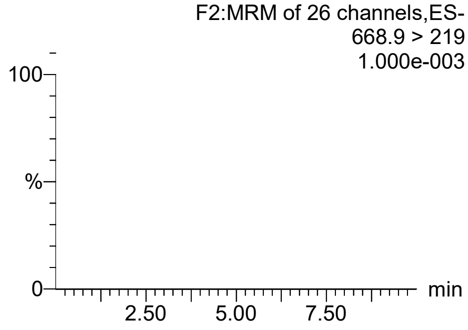

PFTrDA

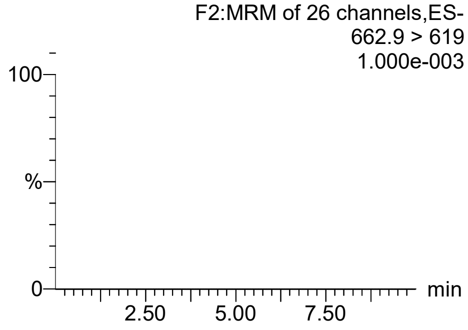

PFDoDA

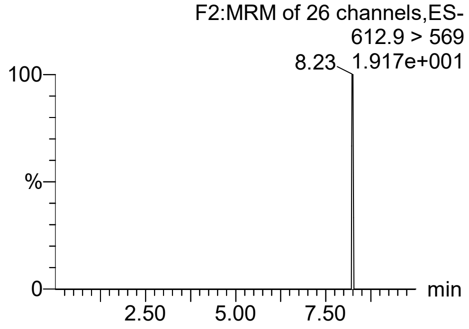

PFUNA

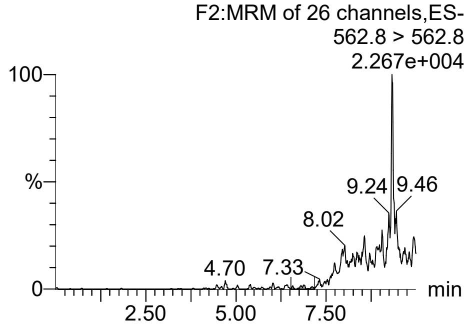

PFOS

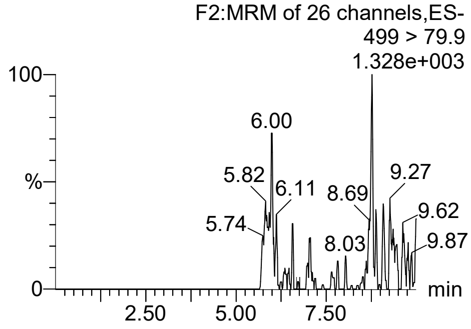

PFNA

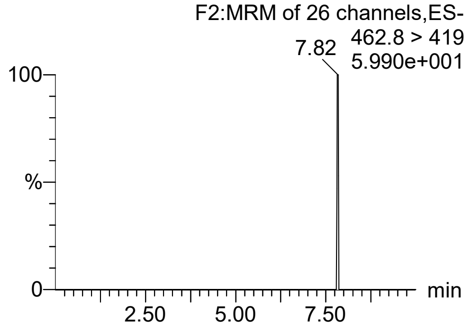

H4PFOS(6:2)

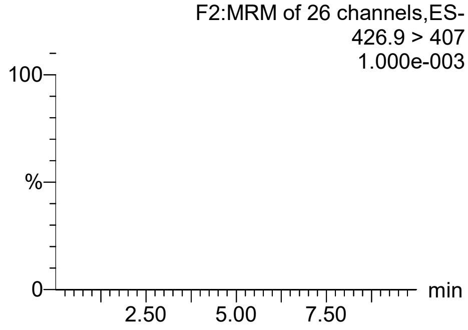

PFOA

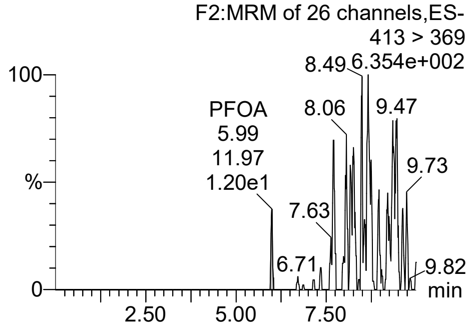

PFHPA

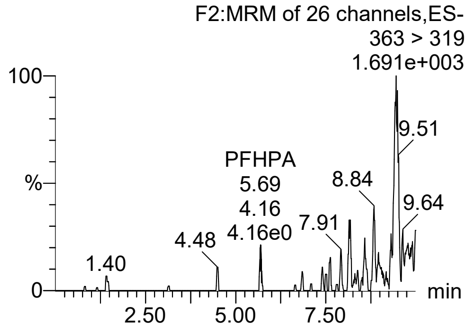

PFHxA

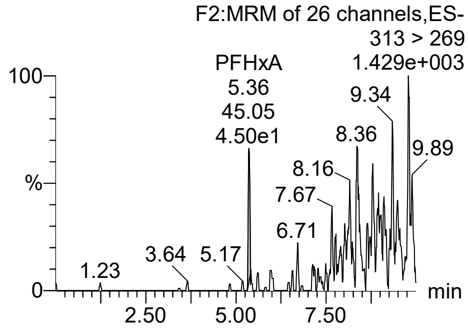

PFPA

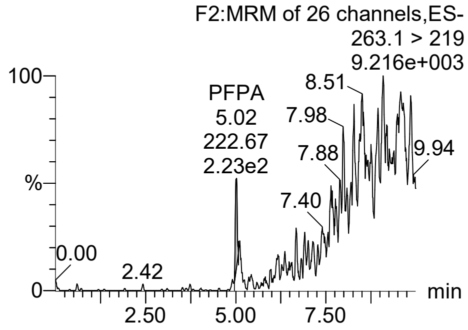

Reviewer:

Dataset: D:\PFCs.PRO\20220704-1.qld

Last Altered: Friday, July 08, 2022 14:11:53 China Standard Time
Printed: Friday, July 08, 2022 14:12:14 China Standard Time

Name: WTF22F06132107C-5, Date: 02-Jul-2022, Time: 14:08:10, ID: , Description: WTF22F06132107C-5

PFDS

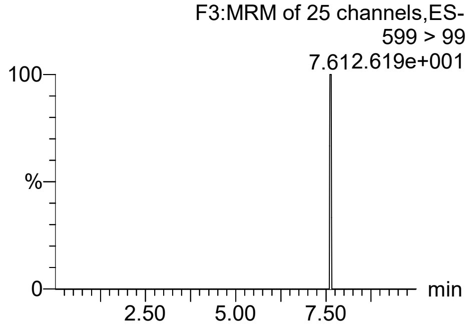

PFDA

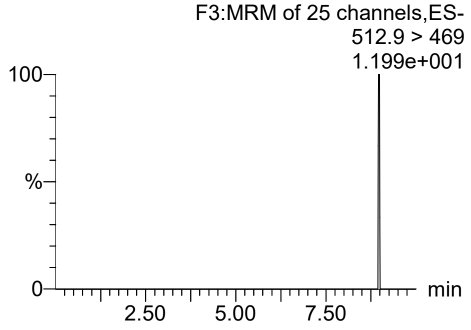

4HPFUnA

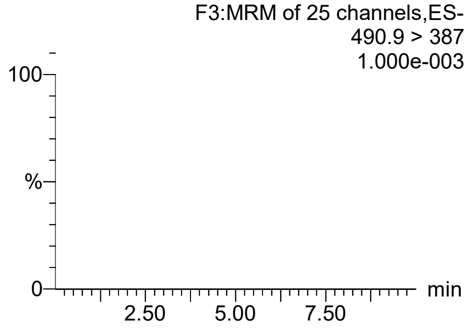

PF-3

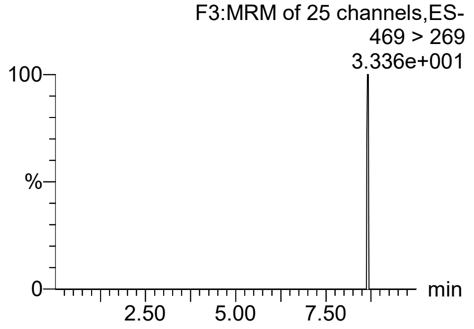

PFHpS

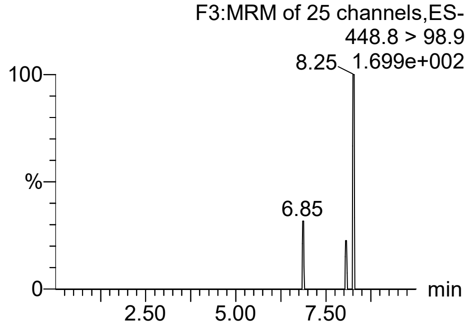

PFHxS

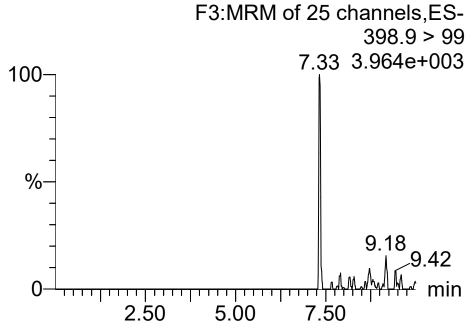

HPFHpA

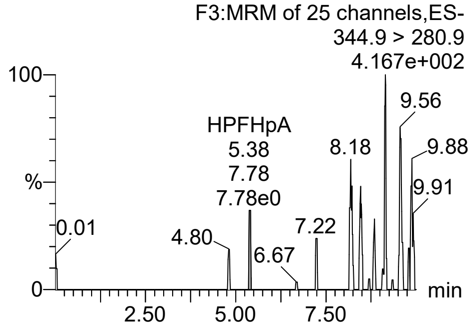

PFBS

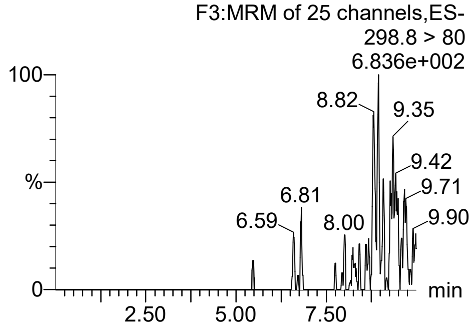

EtFOSE

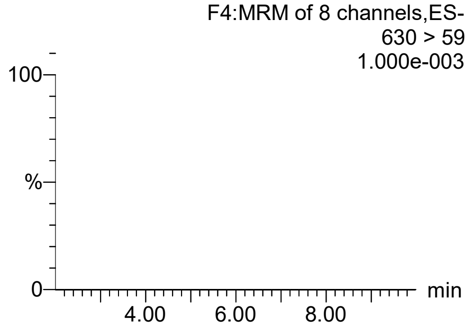

MeFOSE

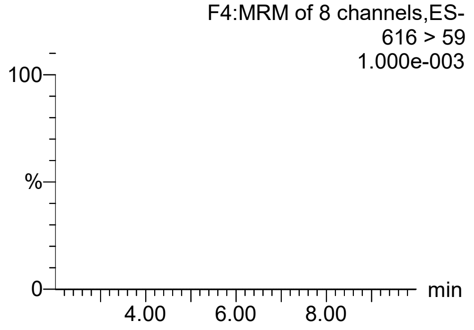

EtFOSA

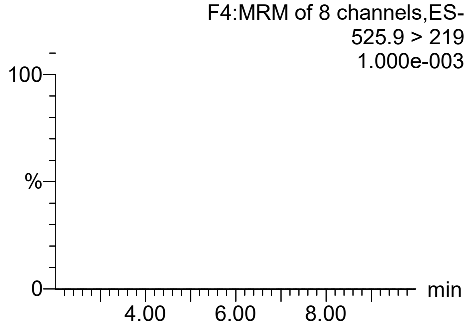

MeFOSA

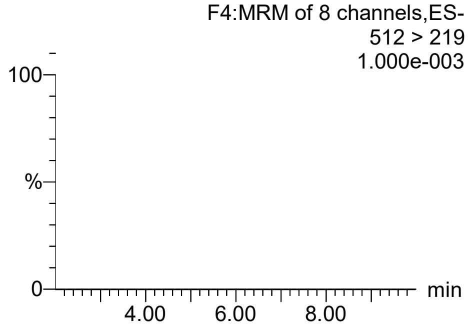

Dataset: D:\PFCs.PRO\20220704-1.qld

Last Altered: Friday, July 08, 2022 14:11:53 China Standard Time
Printed: Friday, July 08, 2022 14:12:14 China Standard Time

Name: WTF22F06132107C-6, Date: 02-Jul-2022, Time: 14:21:19, ID: , Description: WTF22F06132107C-6

PFBA

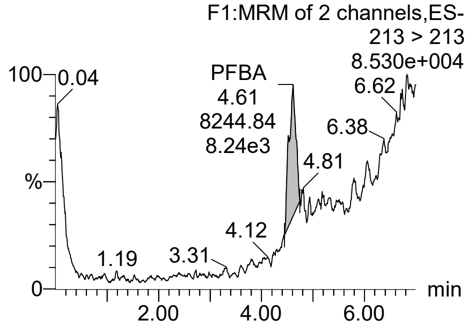

PFTeDA

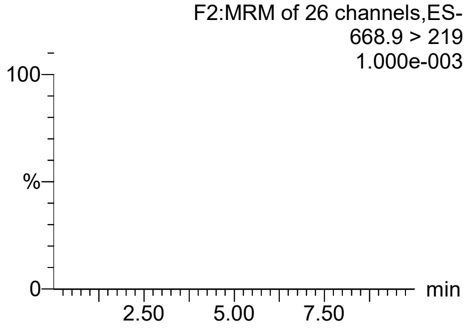

PFTrDA

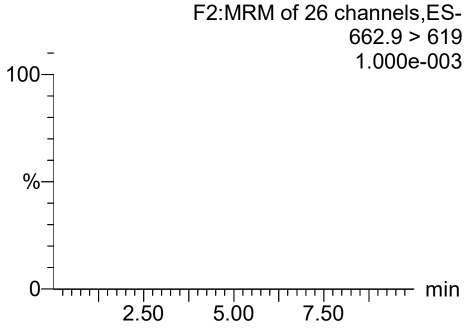

PFDoDA

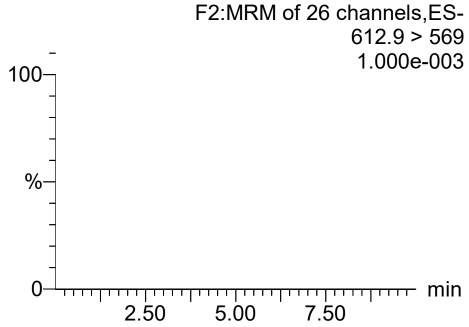

PFUNA

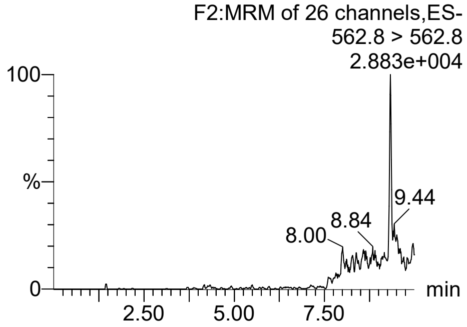

PFOS

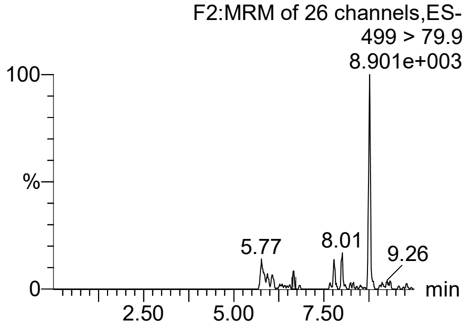

PFNA

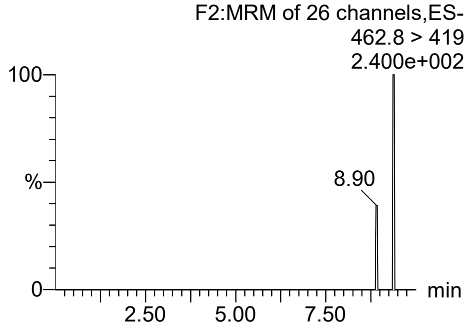

H4PFOS(6:2)

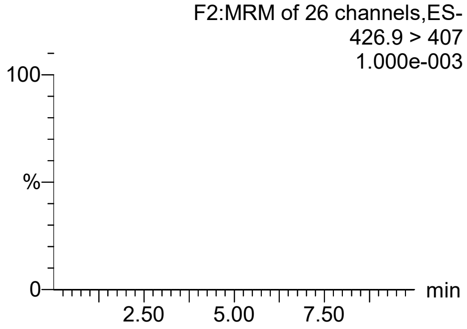

PFOA

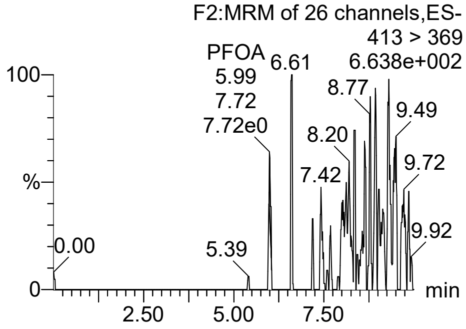

PFHPA

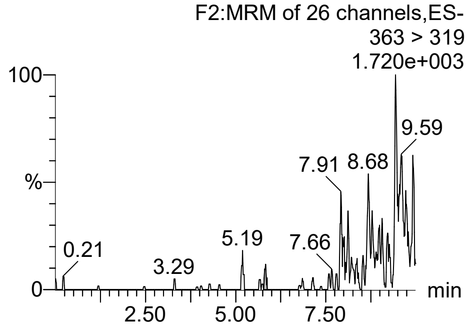

PFHxA

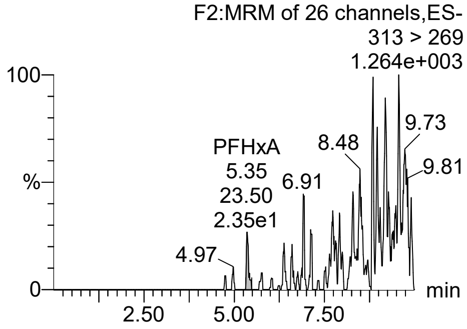

PFPA

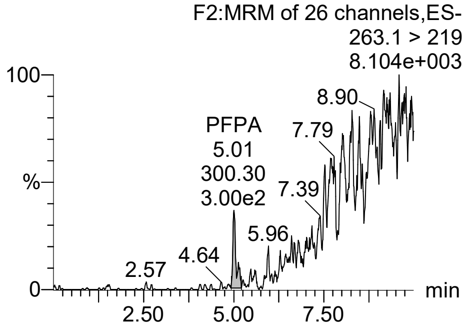

Dataset: D:\PFCs.PRO\20220704-1.qld

Last Altered: Friday, July 08, 2022 14:11:53 China Standard Time
Printed: Friday, July 08, 2022 14:12:14 China Standard Time

Name: WTF22F06132107C-6, Date: 02-Jul-2022, Time: 14:21:19, ID: , Description: WTF22F06132107C-6

PFDS

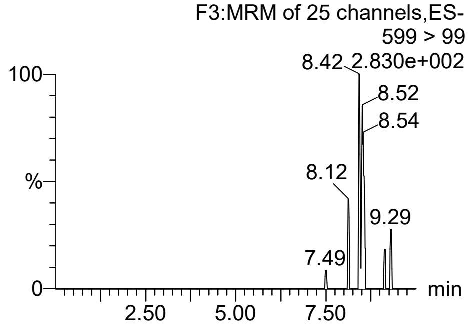

PFDA

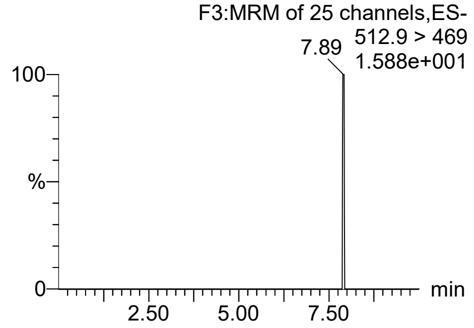

4HPFUnA

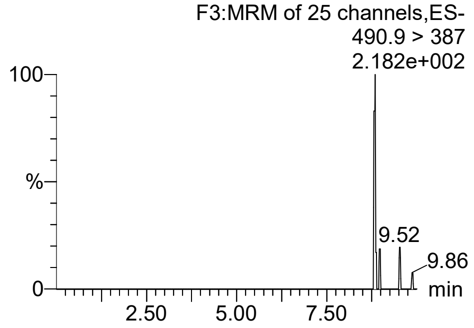

PF-3

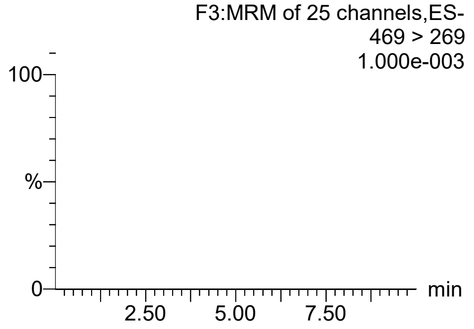

PFHpS

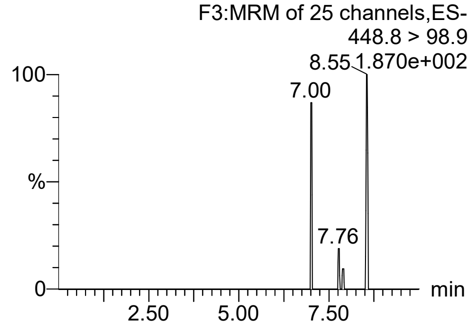

PFHxS

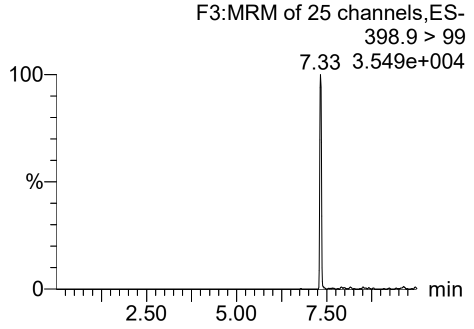

HPFHpA

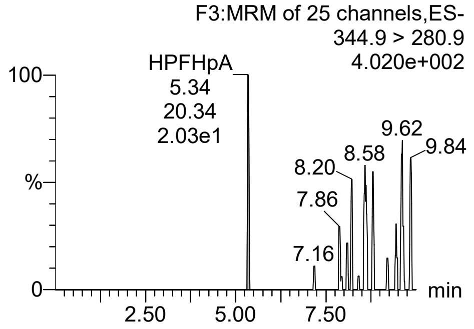

PFBS

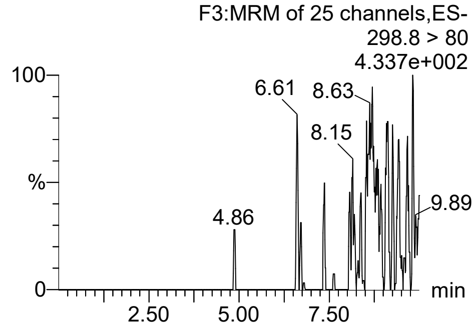

EtFOSE

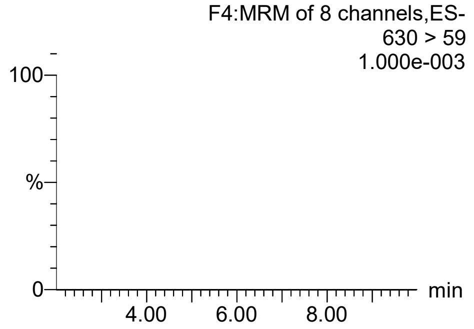

MeFOSE

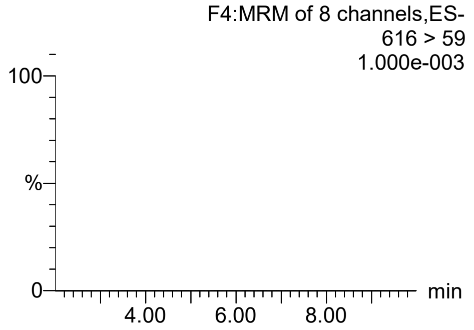

EtFOSA

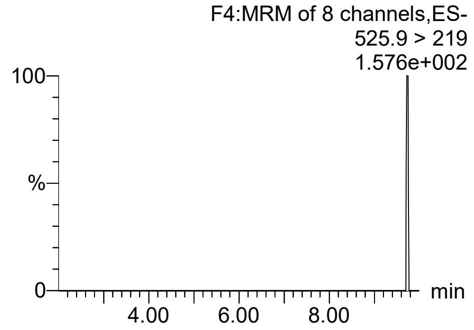

MeFOSA

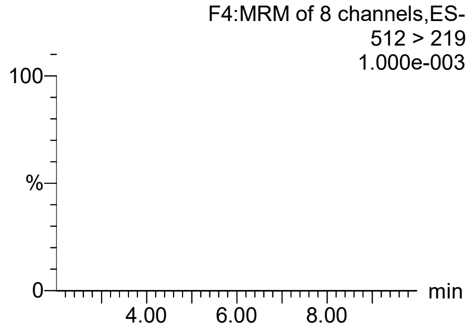

Dataset: D:\PFCs.PRO\20221126.qld

Last Altered: Saturday, November 26, 2022 18:11:13 China Standard Time
Printed: Saturday, November 26, 2022 18:16:05 China Standard Time

Name: WTF22F11234969C-1, Date: 26-Nov-2022, Time: 10:57:25, ID: , Description: WTF22F11234969C-1

PFBA

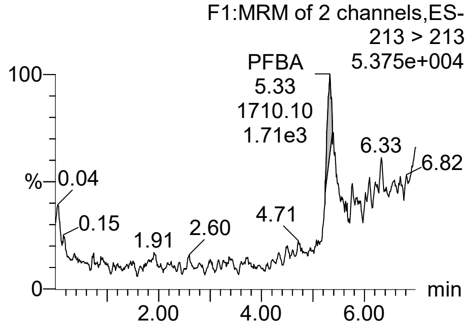

PFTeDA

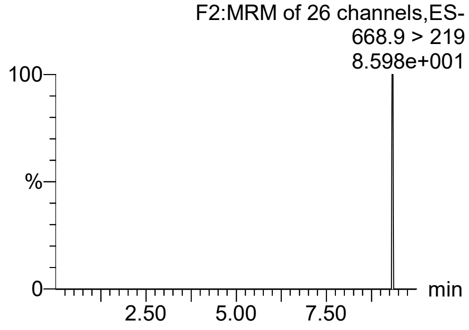

PFTrDA

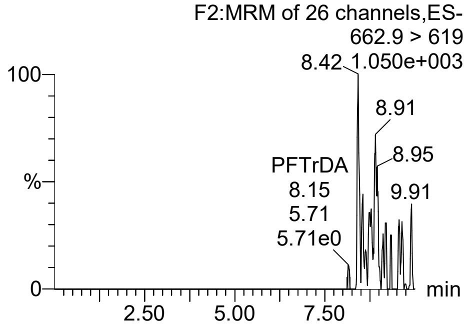

PFDoDA

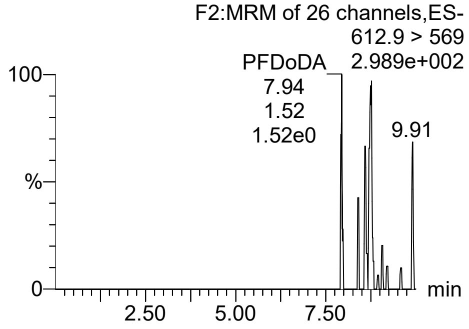

PFUNA

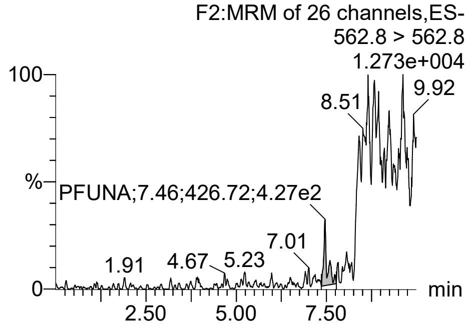

PFOS

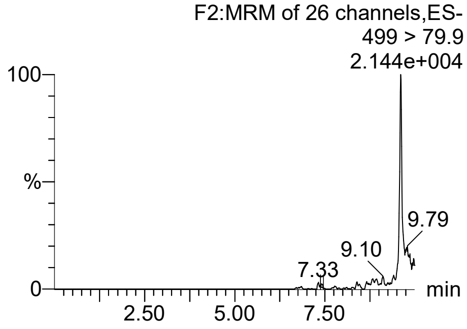

PFNA

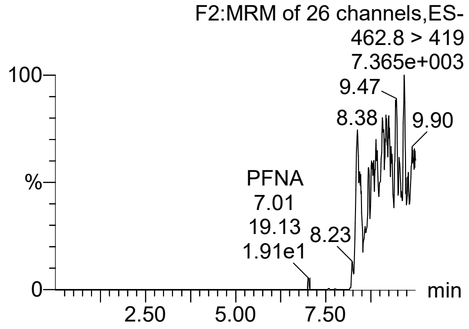

H4PFOS(6:2)

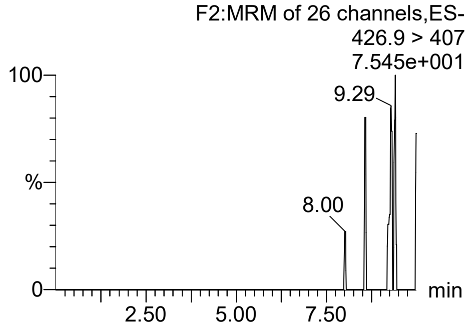

PFOA

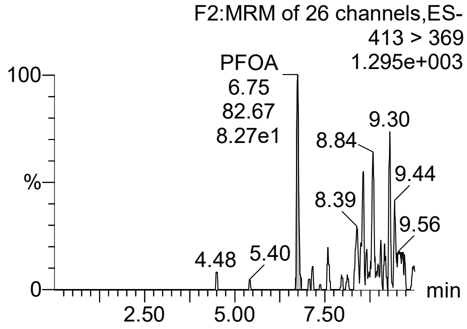

PFHPA

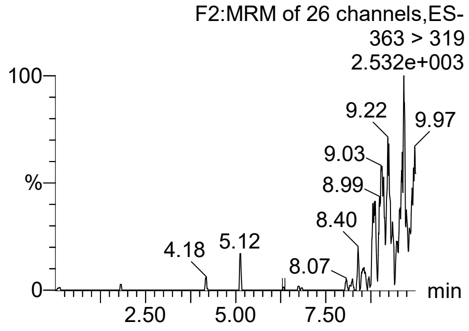

PFHxA

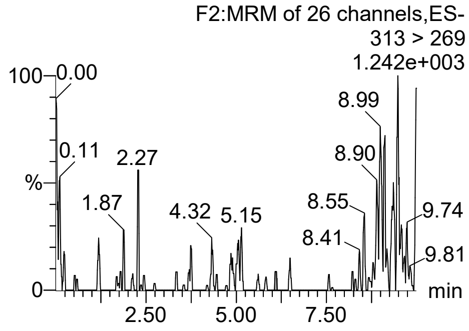

PFPA

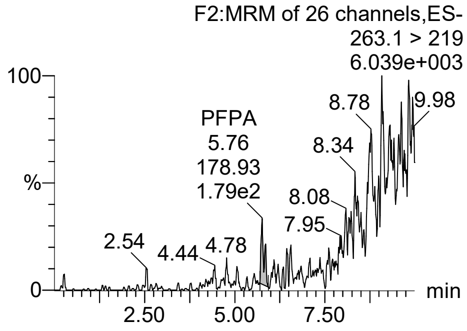

Dataset: D:\PFCs.PRO\20221126.qld

Last Altered: Saturday, November 26, 2022 18:11:13 China Standard Time
Printed: Saturday, November 26, 2022 18:16:05 China Standard Time

Name: WTF22F11234969C-1, Date: 26-Nov-2022, Time: 10:57:25, ID: , Description: WTF22F11234969C-1

PFDS

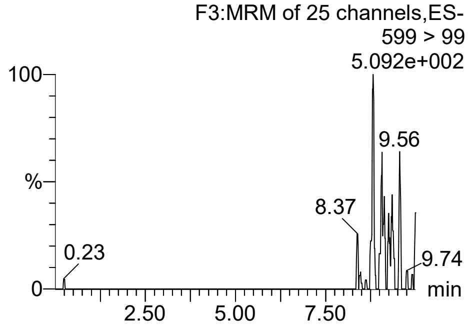

PFDA

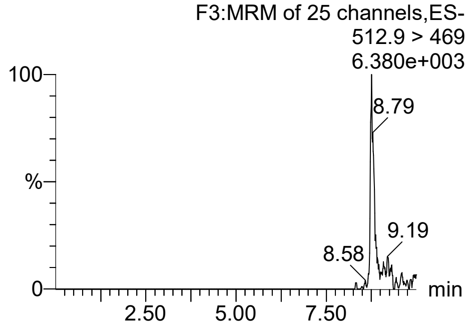

4HPFUnA

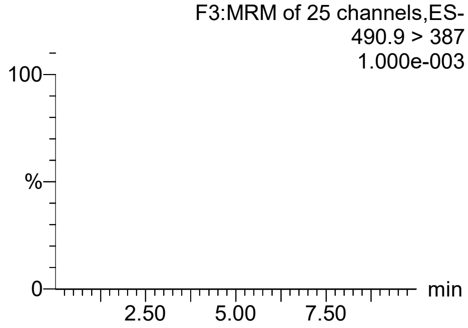

PF-3

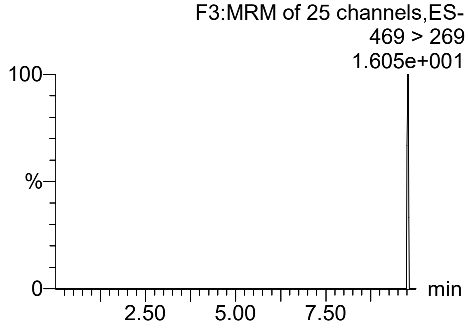

PFHpS

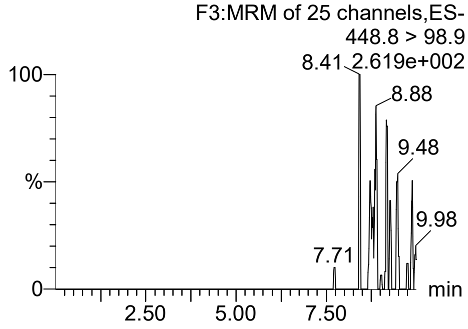

PFHxS

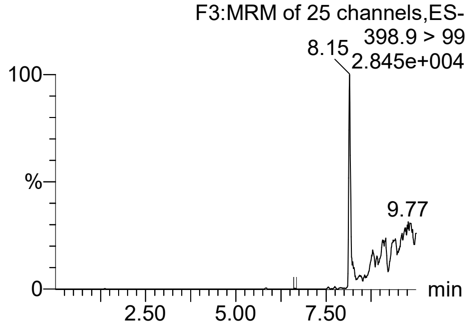

HPFHpA

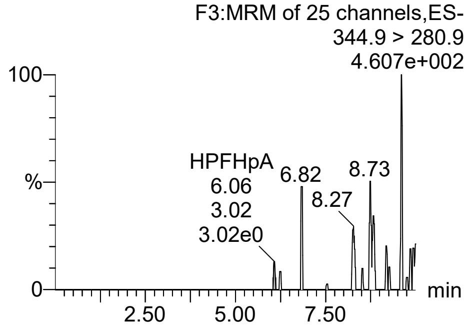

PFBS

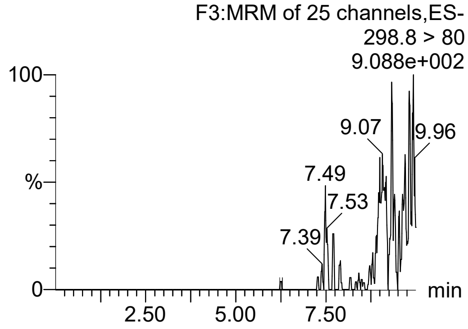

EtFOSE

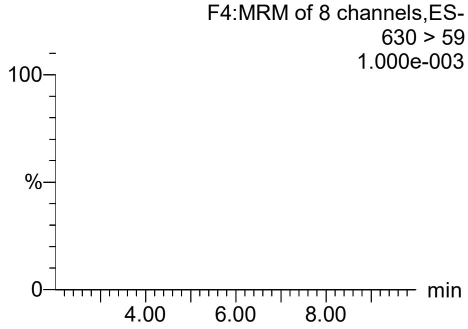

MeFOSE

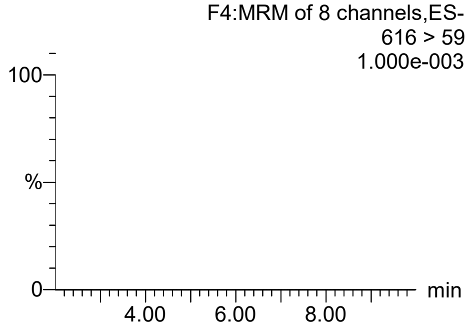

EtFOSA

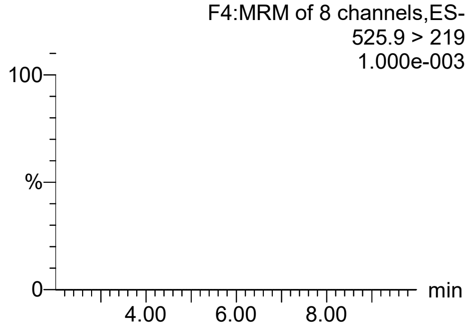

MeFOSA

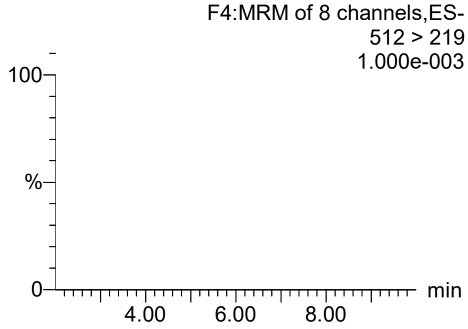

Dataset: D:\PFCs.PRO\20221126.qld

Last Altered: Saturday, November 26, 2022 18:11:13 China Standard Time
Printed: Saturday, November 26, 2022 18:16:05 China Standard Time

Name: WTF22F11234969C-2, Date: 26-Nov-2022, Time: 11:10:42, ID: , Description: WTF22F11234969C-2

PFBA

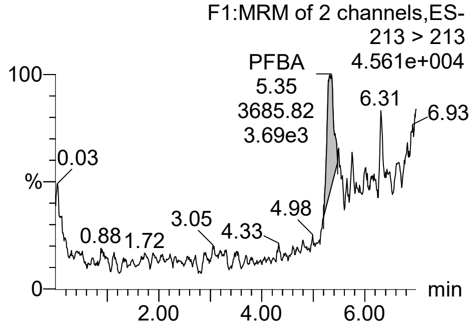

PFTeDA

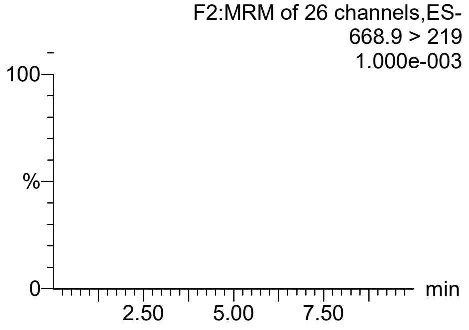

PFTrDA

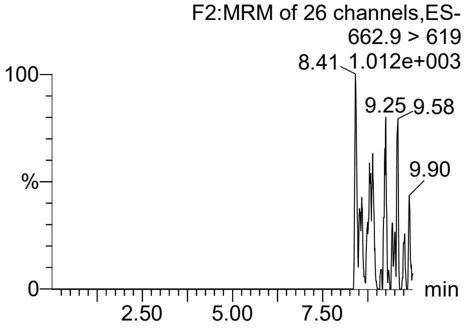

PFDoDA

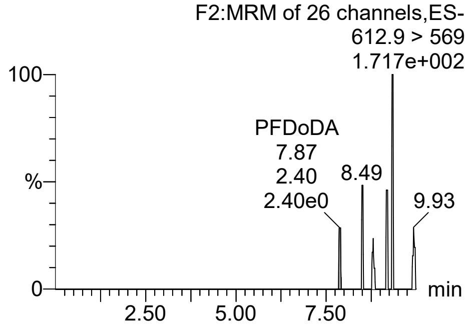

PFUNA

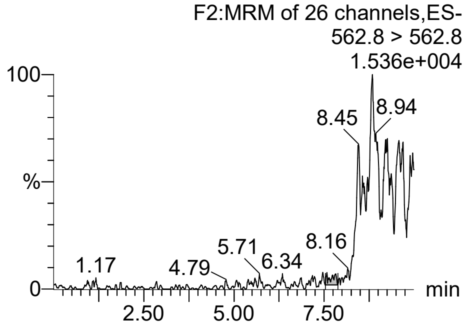

PFOS

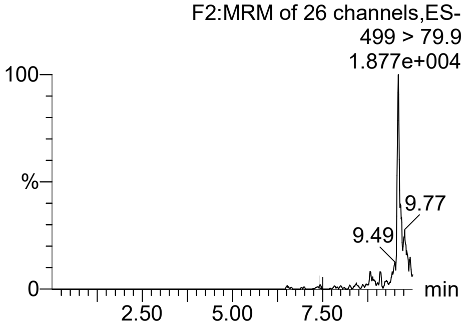

PFNA

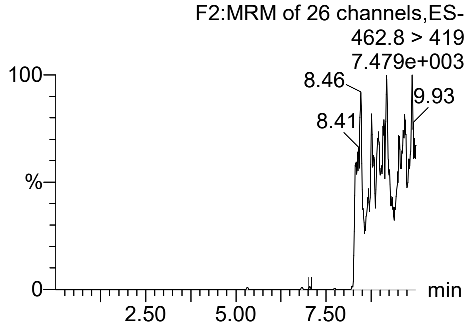

H4PFOS(6:2)

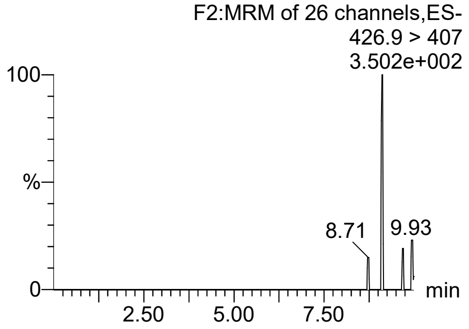

PFOA

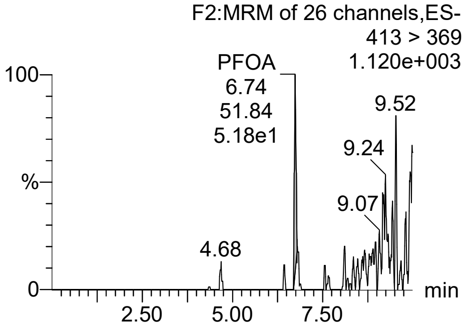

PFHPA

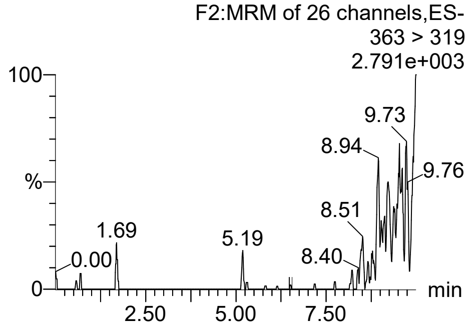

PFHxA

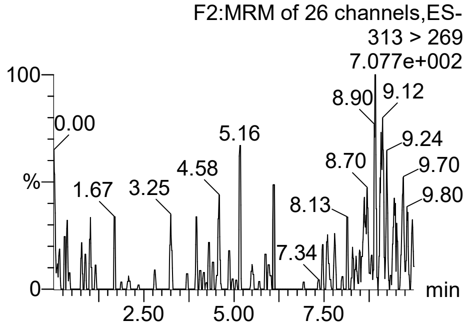

PFPA

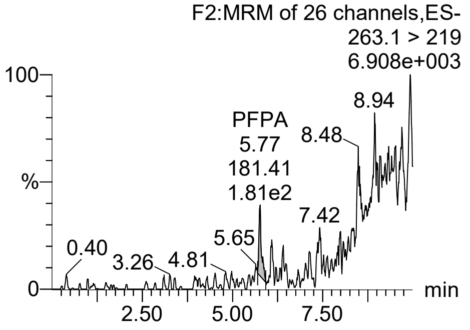

Dataset: D:\PFCs.PRO\20221126.qld

Last Altered: Saturday, November 26, 2022 18:11:13 China Standard Time
Printed: Saturday, November 26, 2022 18:16:05 China Standard Time

Name: WTF22F11234969C-2, Date: 26-Nov-2022, Time: 11:10:42, ID: , Description: WTF22F11234969C-2

PFDS

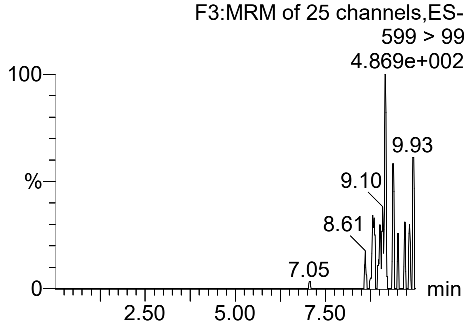

PFDA

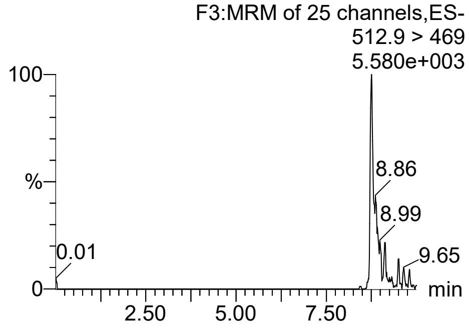

4HPFUnA

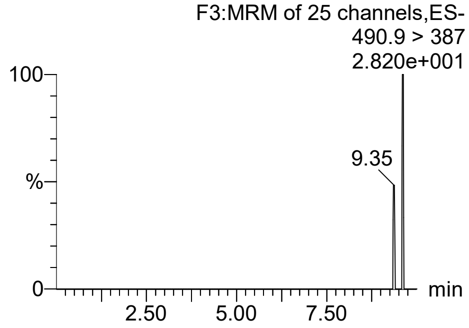

PF-3

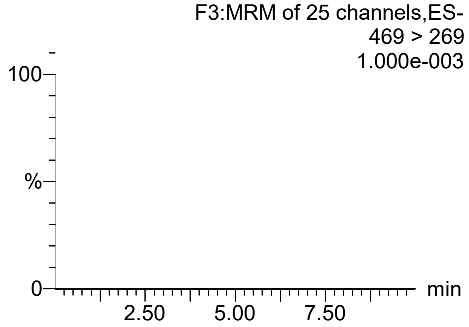

PFHpS

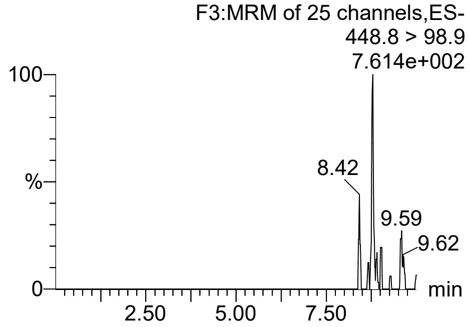

PFHxS

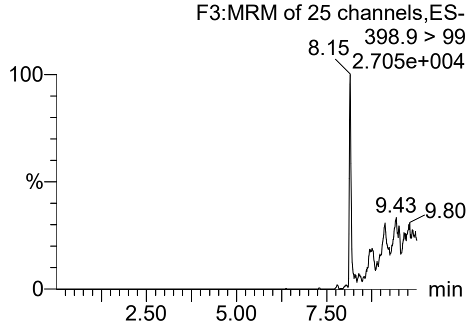

HPFHpA

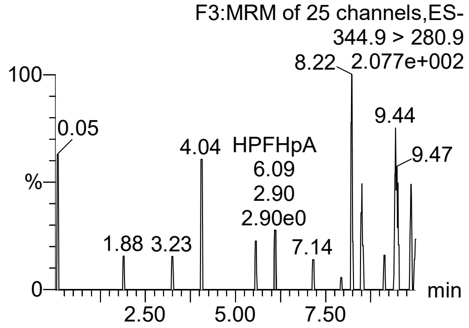

PFBS

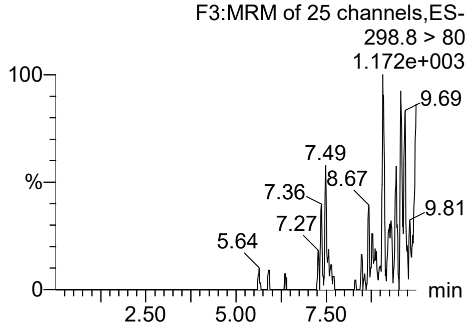

EtFOSE

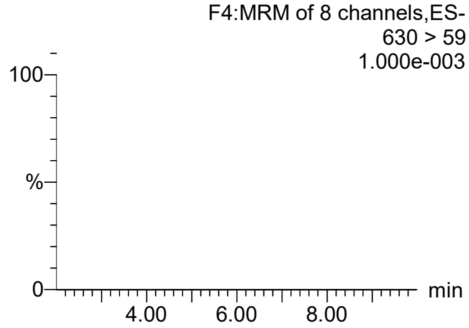

MeFOSE

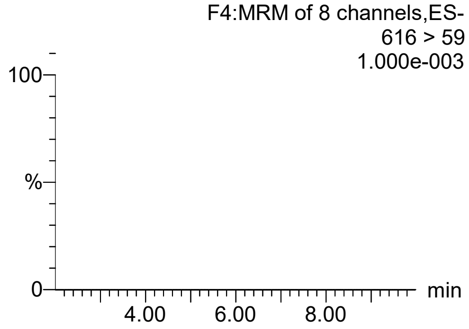

EtFOSA

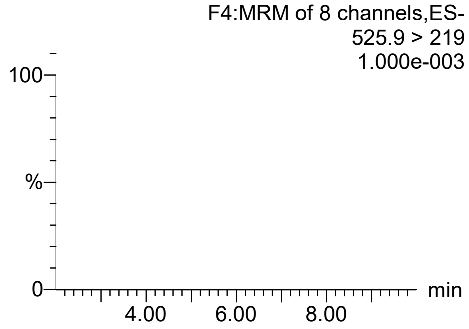

MeFOSA

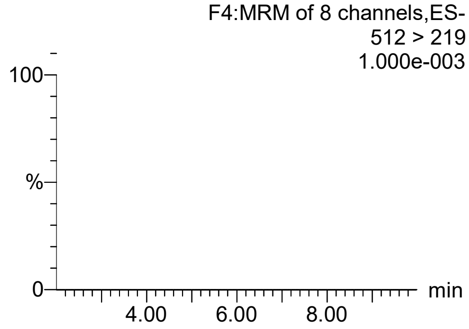

Dataset: D:\PFCs.PRO\20221126.qld

Last Altered: Saturday, November 26, 2022 18:11:13 China Standard Time
Printed: Saturday, November 26, 2022 18:16:05 China Standard Time

Name: WTF22F11234969C-3, Date: 26-Nov-2022, Time: 11:23:58, ID: , Description: WTF22F11234969C-3

PFBA

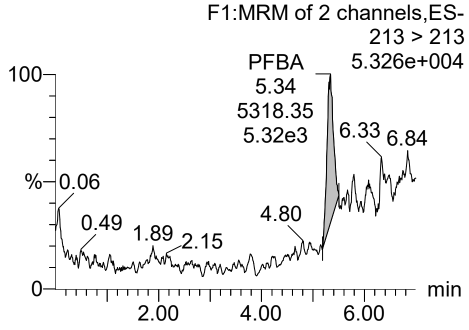

PFTeDA

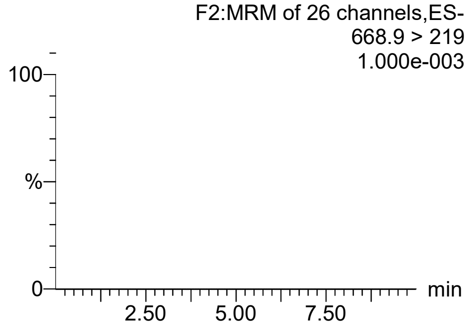

PFTrDA

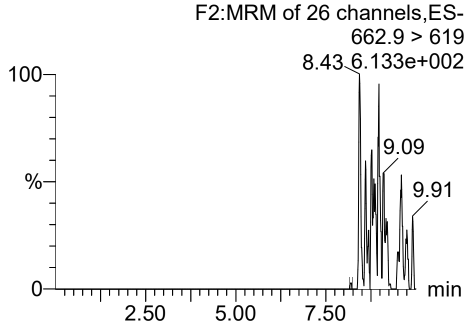

PFDoDA

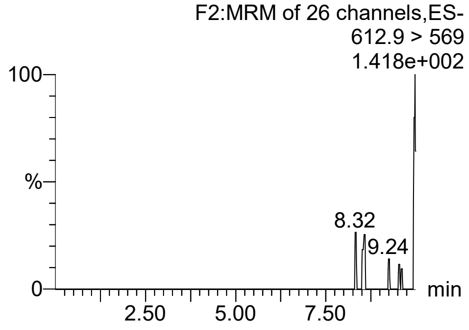

PFUNA

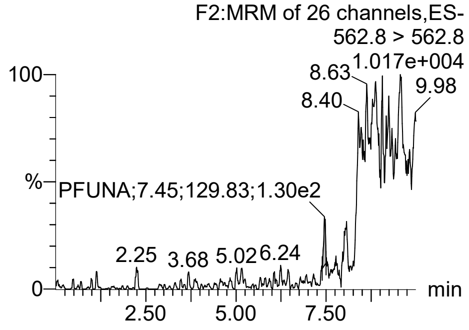

PFOS

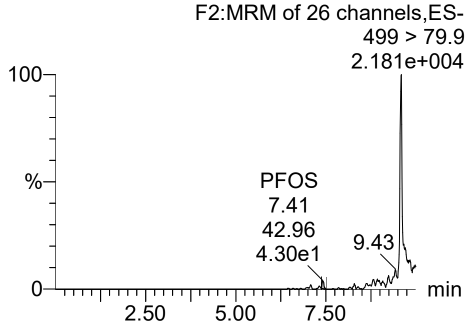

PFNA

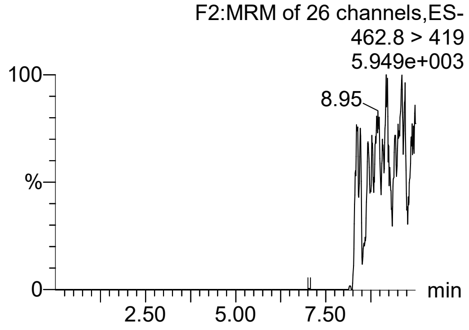

H4PFOS(6:2)

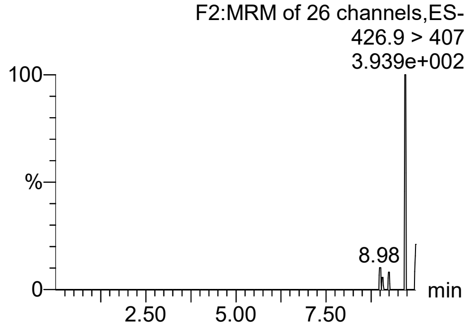

PFOA

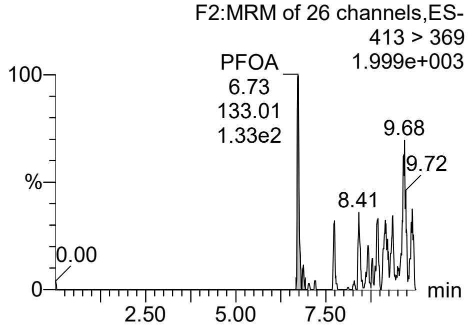

PFHPA

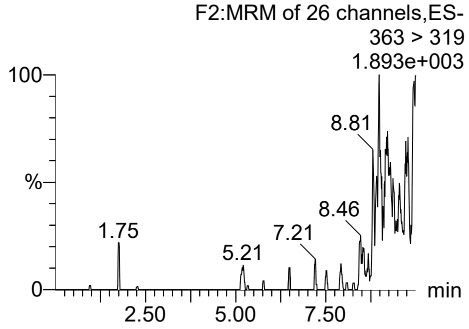

PFHxA

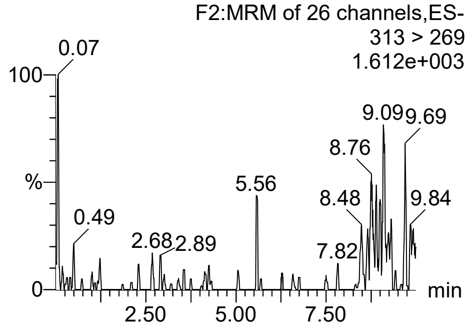

PFPA

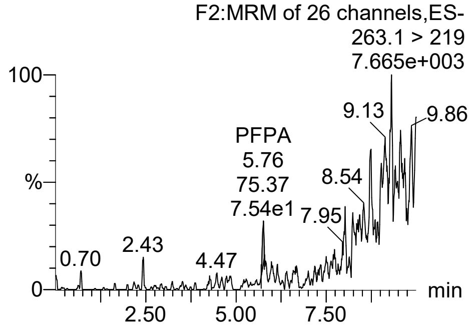

Dataset: D:\PFCs.PRO\20221126.qld

Last Altered: Saturday, November 26, 2022 18:11:13 China Standard Time
Printed: Saturday, November 26, 2022 18:16:05 China Standard Time

Name: WTF22F11234969C-3, Date: 26-Nov-2022, Time: 11:23:58, ID: , Description: WTF22F11234969C-3

PFDS

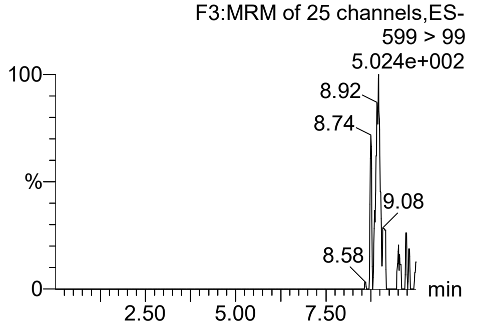

PFDA

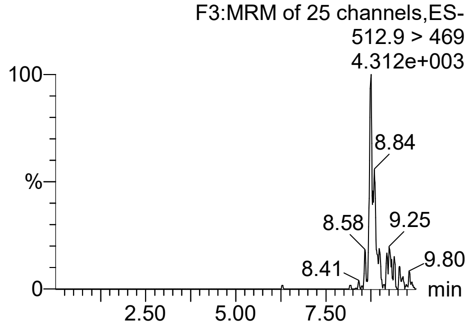

4HPFUnA

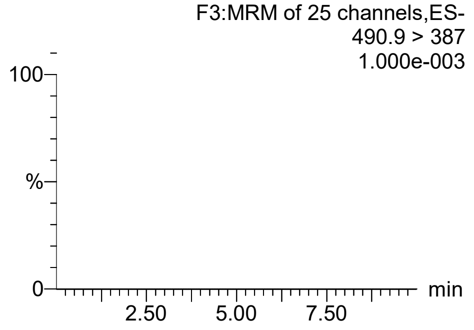

PF-3

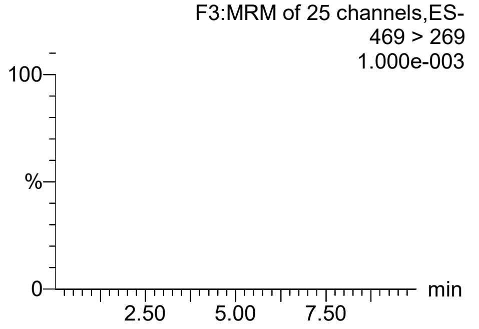

PFHpS

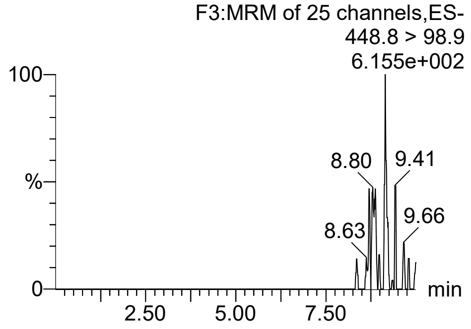

PFHxS

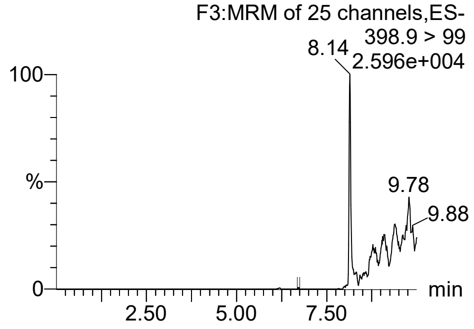

HPFHpA

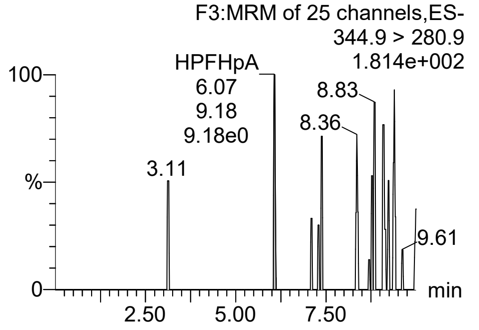

PFBS

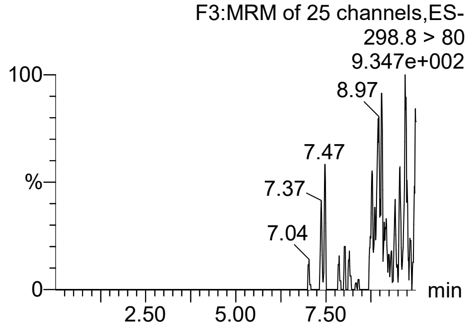

EtFOSE

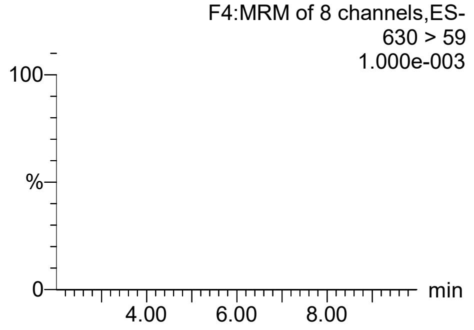

MeFOSE

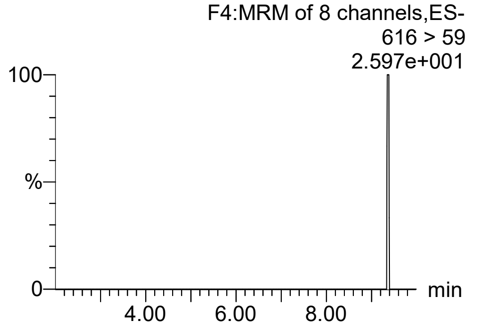

EtFOSA

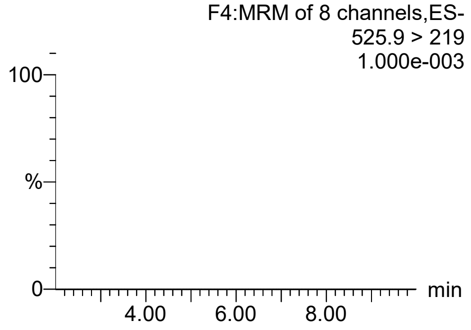

MeFOSA

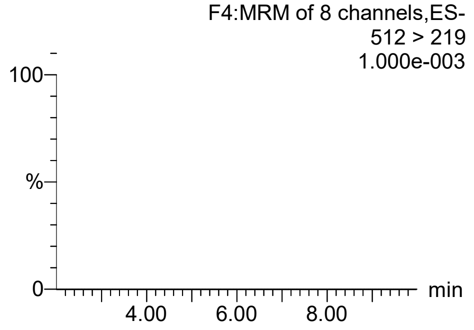

Dataset: D:\PFCs.PRO\20221126.qld

Last Altered: Saturday, November 26, 2022 18:11:13 China Standard Time
Printed: Saturday, November 26, 2022 18:16:05 China Standard Time

Name: WTF22F11234969C-4, Date: 26-Nov-2022, Time: 11:37:14, ID: , Description: WTF22F11234969C-4

PFBA

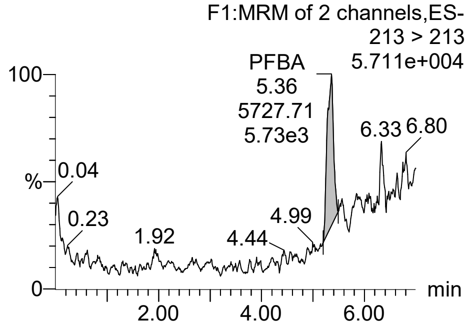

PFTeDA

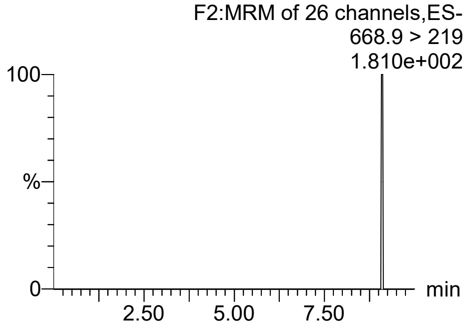

PFTrDA

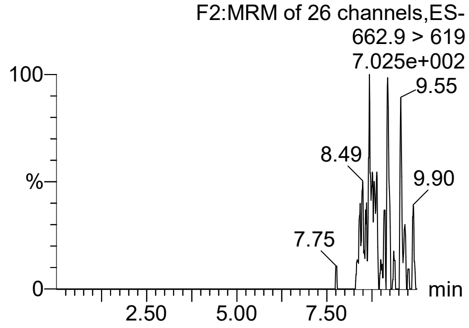

PFDoDA

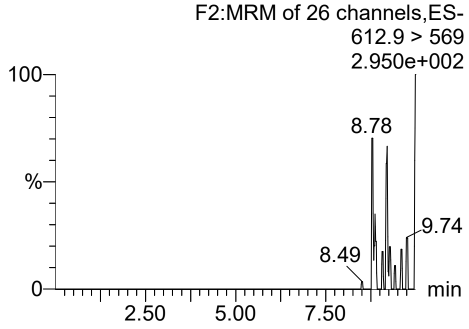

PFUNA

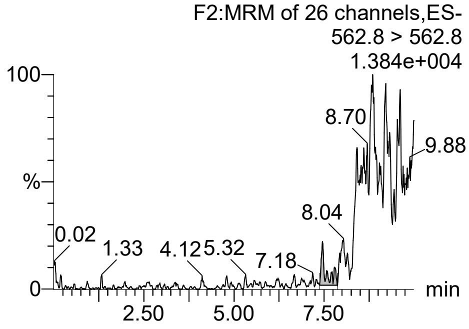

PFOS

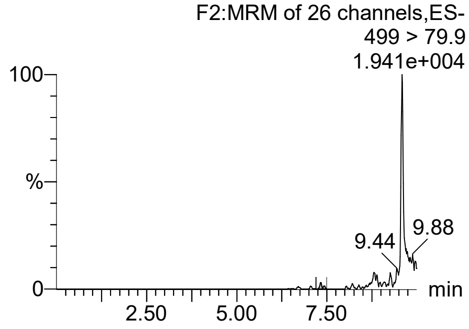

PFNA

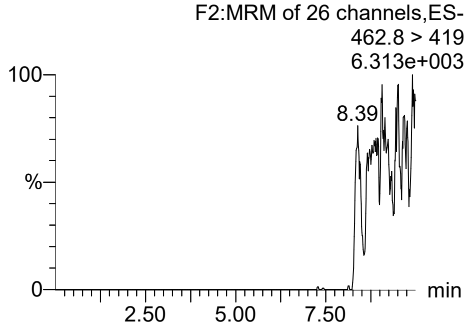

H4PFOS(6:2)

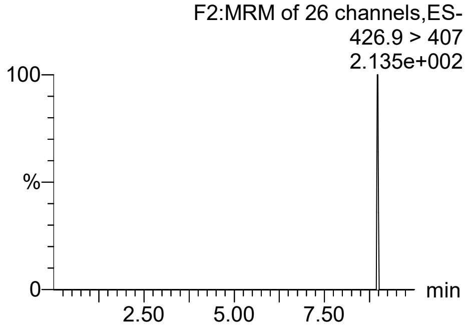

PFOA

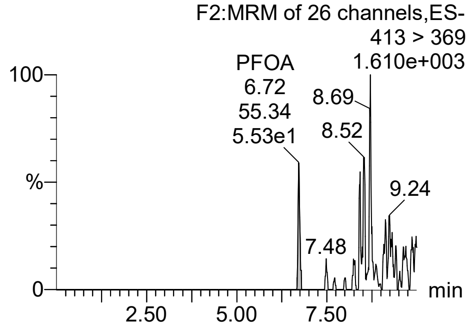

PFHPA

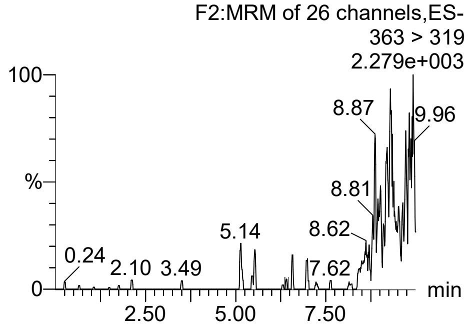

PFHxA

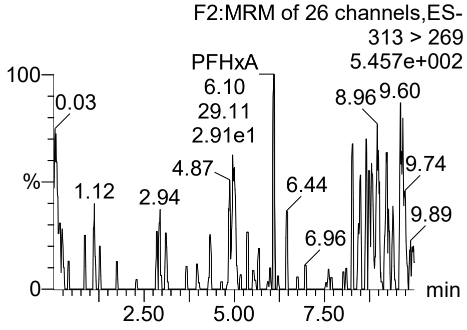

PFPA

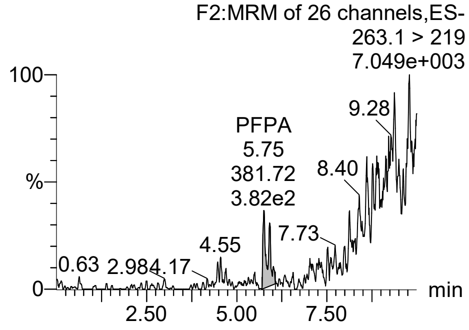

Dataset: D:\PFCs.PRO\20221126.qld

Last Altered: Saturday, November 26, 2022 18:11:13 China Standard Time
Printed: Saturday, November 26, 2022 18:16:05 China Standard Time

Name: WTF22F11234969C-4, Date: 26-Nov-2022, Time: 11:37:14, ID: , Description: WTF22F11234969C-4

PFDS

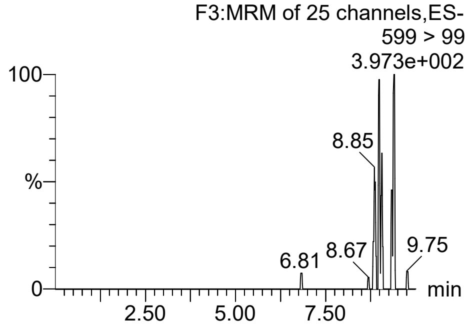

PFDA

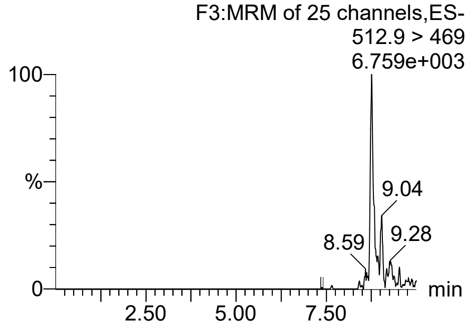

4HPFUnA

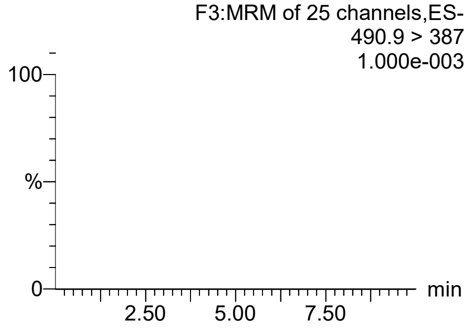

PF-3

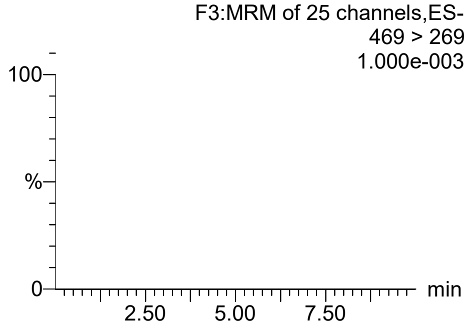

PFHpS

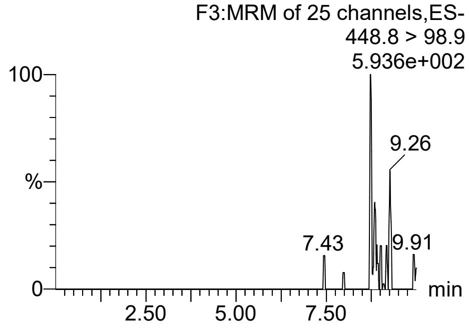

PFHxS

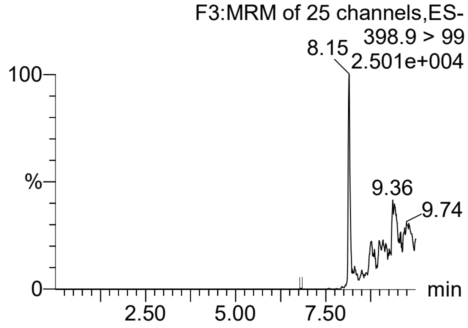

HPFHpA

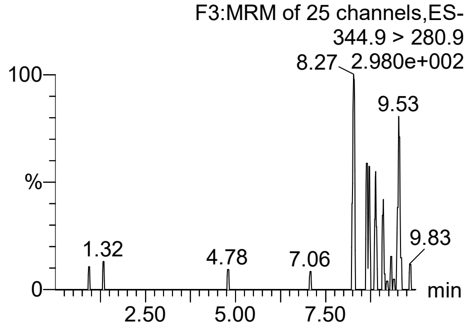

PFBS

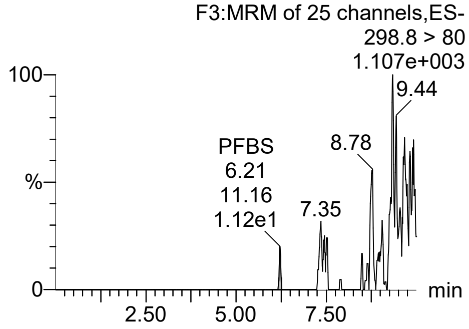

EtFOSE

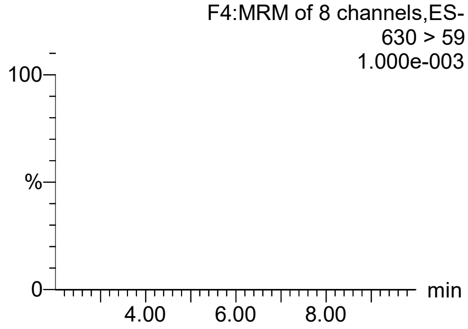

MeFOSE

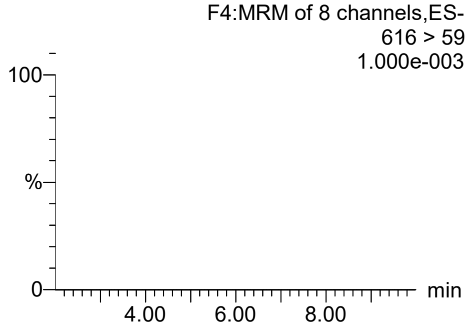

EtFOSA

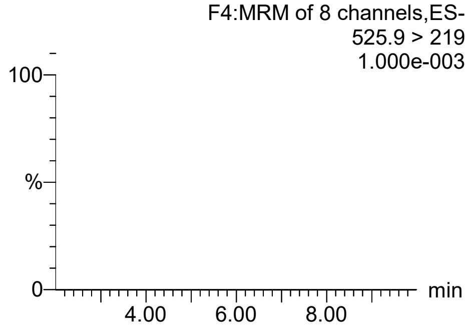

MeFOSA

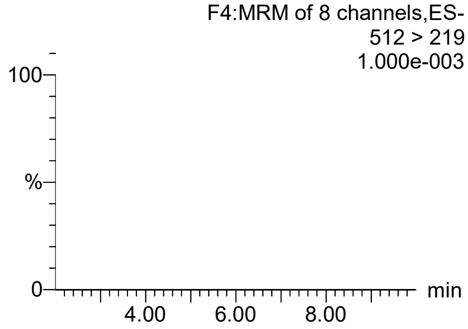

Dataset: D:\PFCs.PRO\20221126.qld

Last Altered: Saturday, November 26, 2022 18:11:13 China Standard Time
Printed: Saturday, November 26, 2022 18:16:05 China Standard Time

Name: WTF22F11234969C-5, Date: 26-Nov-2022, Time: 11:50:13, ID: , Description: WTF22F11234969C-5

PFBA

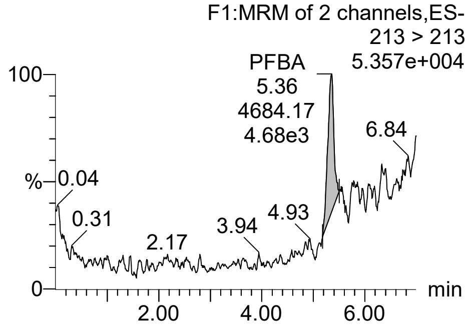

PFTeDA

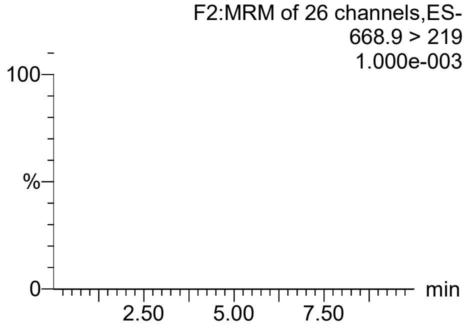

PFTrDA

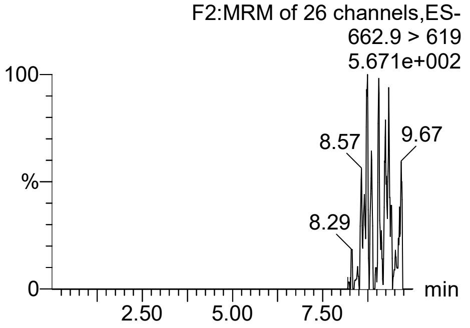

PFDoDA

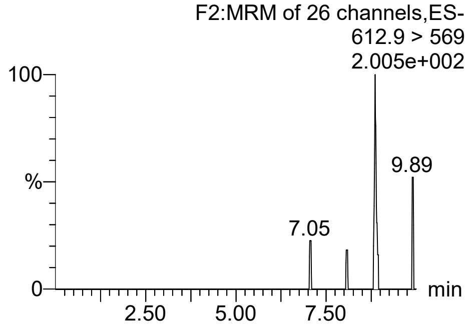

PFUNA

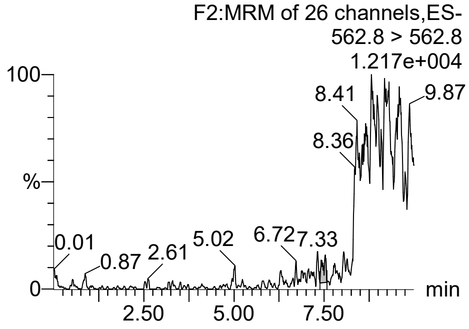

PFOS

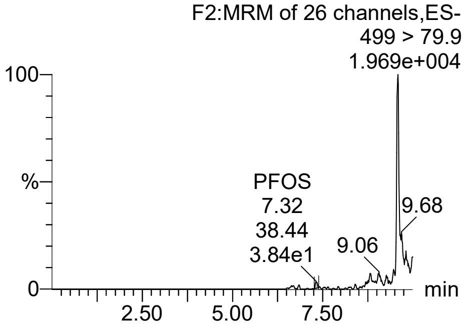

PFNA

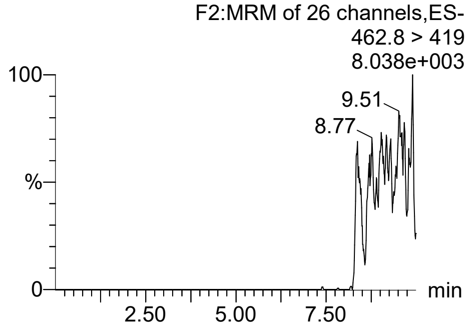

H4PFOS(6:2)

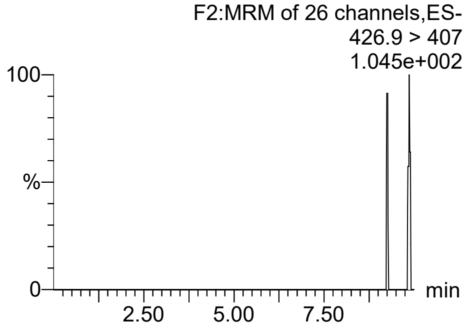

PFOA

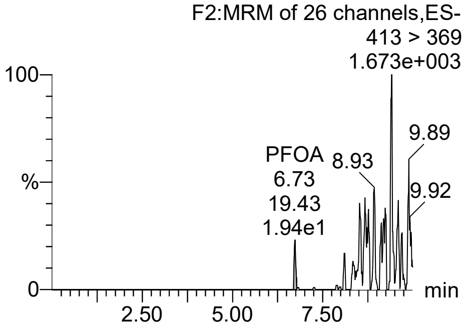

PFHPA

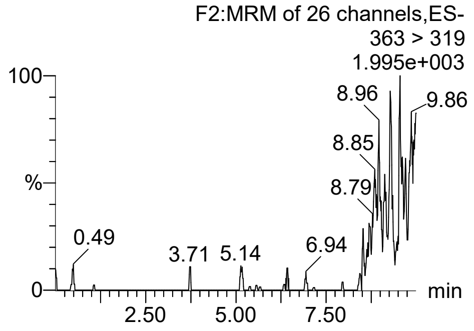

PFHxA

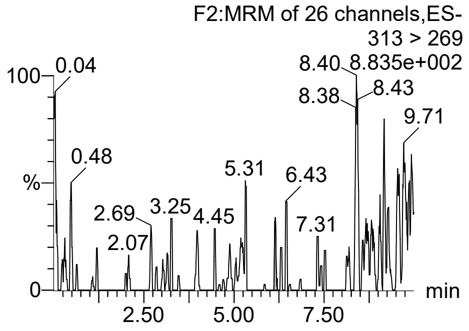

PFPA

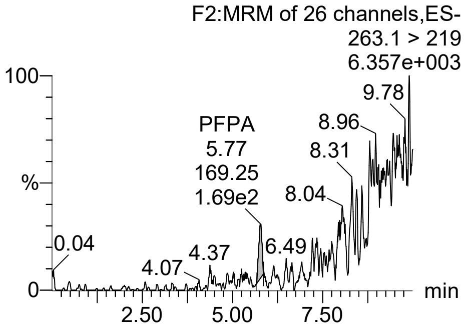

Dataset: D:\PFCs.PRO\20221126.qld

Last Altered: Saturday, November 26, 2022 18:11:13 China Standard Time
Printed: Saturday, November 26, 2022 18:16:05 China Standard Time

Name: WTF22F11234969C-5, Date: 26-Nov-2022, Time: 11:50:13, ID: , Description: WTF22F11234969C-5

PFDS

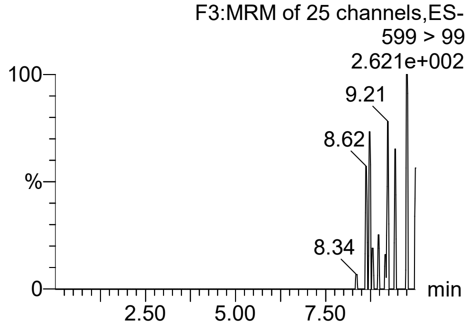

PFDA

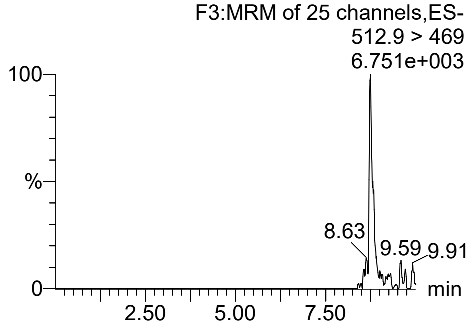

4HPFUnA

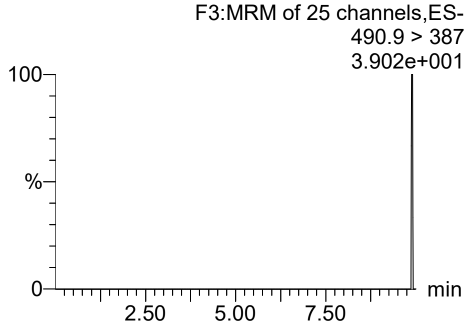

PF-3

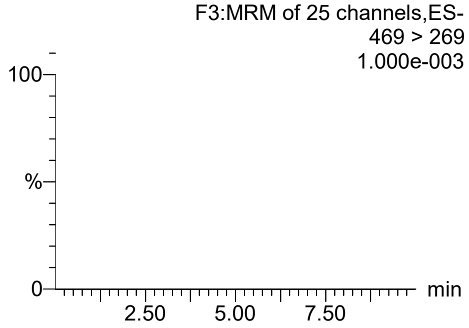

PFHpS

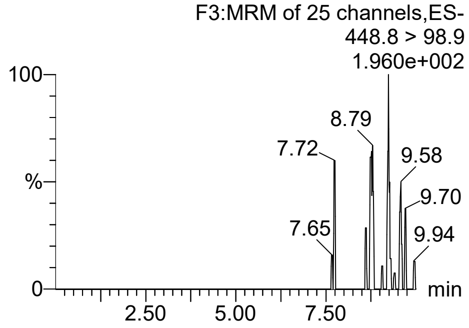

PFHxS

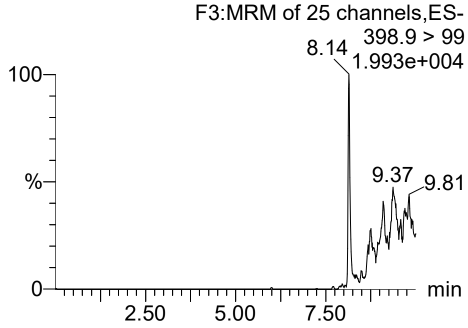

HPFHpA

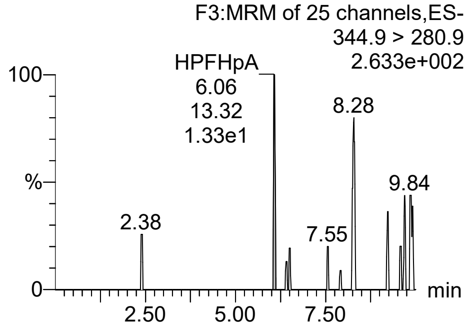

PFBS

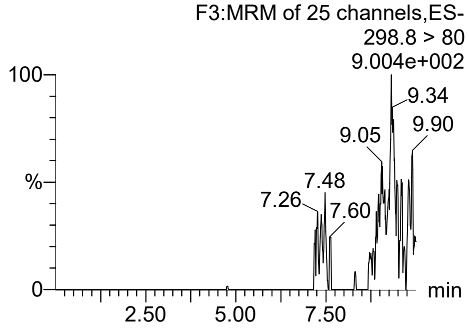

EtFOSE

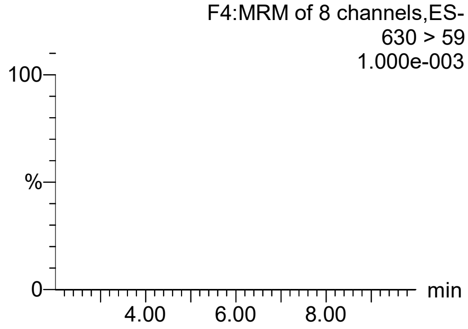

MeFOSE

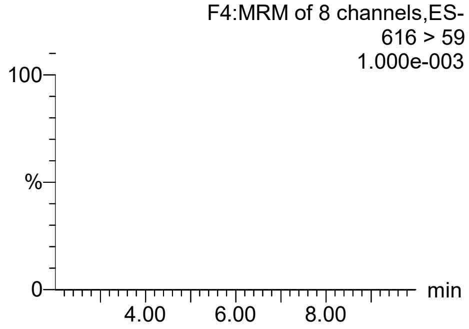

EtFOSA

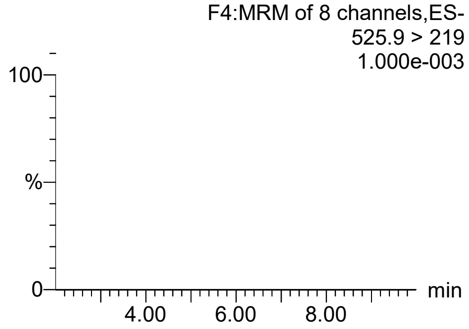

MeFOSA

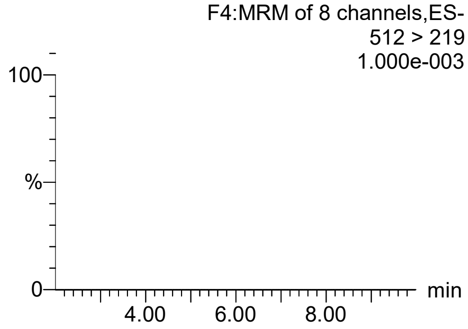

Supplement: Supplementary file 4 — Supplementary Data 1 [file 41467_2024_45077_MOESM4_ESM.pdf]
